# Genome-wide analysis of cork oak NLRs reveals RNL-associated duplications and immune receptor candidates associated with biotic and abiotic stress response

**DOI:** 10.3389/fgene.2026.1894037

**Published:** 2026-10-07

**Authors:** L. M. Gonçalves, M. M. Oliveira, P. M. Barros

**Affiliations:** Instituto de Tecnologia Química e Biológica António Xavier, Oeiras, Portugal

**Keywords:** balancing selection, biotic stress, drought, InterNLR, NLR genes, plant immunity, positive selection, Quercus suber

## Abstract

The Nucleotide-Binding Domain Leucine-Rich Repeat (NLR) gene family is a central component of plant immune systems, yet its diversity and evolutionary dynamics remain poorly characterized in long-lived tree species. Here, we performed a genome-wide analysis of the NLR gene repertoire in *Quercus suber* (cork oak) using a newly developed annotation tool–InterNLR–which identifies and classifies NLRs as canonical and non-canonical. We identified a total of 374 canonical NLRs, and 544 non-canonical NLRs–which may represent pseudogenes, gene fragments or evolutionary intermediates. Phylogenetic analyses based on the NB-ARC domain highlighted the distinct evolutionary trajectory of RNL proteins, which function as “helper” NLRs and show evidence of clade-specific gene duplication. Transcriptomic analyses revealed significant differences in gene-level expression among NLR classes in cork oak stem tissues, mainly driven by contrasts involving the non-canonical N class. Seven cork oak NLR genes, sharing putative orthology with abiotic stress-responsive NLRs found in other species, were also differentially expressed in stems under drought conditions. From these, four candidate NLRs arise as candidate components of shared or convergent responses to drought and *P. cinnamomi* infection. Population-genetic analyses of RNA-seq-derived variants from expressed NLR loci in two Iberian populations further revealed heterogeneous patterns among expressed NLR loci, with a predominance of positive Tajima’s D values. Together, these results provide new insights into the evolution, expression, and functional diversification of NLRs in cork oak. This work advances our understanding of immune gene architecture in an ecologically and economically important forest tree species.

## Introduction

Rising temperatures, altered precipitation patterns and the increasing frequency of extreme weather events are examples of environmental shifts that bring significant challenges to ecosystems worldwide and therefore implications for plant survival (Travis, 2003). Cork oak (*Quercus suber* L.), a sclerophyllous evergreen tree species from the Fagaceae family, is particularly affected, mostly due to its long lifespan (Pina-Martins et al., 2019). This species is mostly distributed in the Western Mediterranean region, where it plays an ecologically important role by maintaining the biodiversity, supporting soil stability and contributing to carbon sequestration (Hidalgo et al., 2008; Tenhunen et al., 2009). Cork oak has also an important role by sustaining traditional agroforestry systems, such as *montado* and *dehesa*, which are crucial for local economies and cultural heritage (Besson et al., 2014).

Drought is one of the greatest challenges for the survival of cork oak stands since water stress events impose a significant threat to tree vitality (Cook et al., 2018). During drought adaptation, water uptake from the roots and transport capacity in the xylem is reduced and affects the overall hydraulic function of the tree (Chaves et al., 2003). This results in decreased tree growth, wilting, and even mortality of cork oak trees. Some studies have shown that drought conditions can alter the anatomy and physiology of xylem vessels–reducing vessel size and increasing embolism formation–therefore compromising water transport efficiency (Camilo-Alves et al., 2017; Besson et al. 2014). Drought can also reduce tree defences, increasing susceptibility to pathogens, as observed in other tree species (McDowell et al., 2008). In the case of cork oak, drought has been shown to enhance the vulnerability of the trees to pathogens induced by stress such as the fungus *Hypoxylon mediterraneum* (Vannini et al., 1996) and the oomycete *Phytophthora cinnamomi* (Moreira et al., 2006). Therefore, it is important to understand the interplay between drought and biotic stress for effective forest management and conservation strategies.

In response to biotic stimuli, plants rely on their innate immune system to defend themselves from pathogen infection. Many pathogens deliver effector proteins to the intracellular environment to manipulate host metabolism. In turn, host plants have developed a second level of surveillance and effector recognition, the effector-triggered immunity, which is mediated by intracellular immune receptor proteins named Nucleotide-binding Leucine-rich-repeats (NLRs). These receptors are related to specific innate immunity receptors in animals. After detecting specific effectors, NLRs trigger a hypersensitive response that may lead to cell death at the infection site, thus preventing the expansion of the pathogen (Wu et al., 2017).

NLRs are multi-domain proteins with a conserved modular architecture. Canonically, they comprise a variable N-terminal domain, which often functions as a signalling domain, a central nucleotide-binding (NB) domain that contains two ARC domains, and a C-terminal leucine-rich repeat (LRR) domain that plays a role in sensing ligands and potentially also in activity regulation (Jones et al., 2016). NLRs detect effector proteins secreted by pathogens through direct or indirect interactions. For example, they can directly bind effector proteins or indirectly interact with them through effector-targeted host proteins. An emerging model suggests that some “sensor” NLRs, responsible for detecting pathogen effectors, require “helper” NLRs to initiate immune signalling. “Sensor” NLRs detect pathogen effectors either directly or indirectly by monitoring effector-induced modifications of host proteins. In contrast, “helper” NLRs function downstream of sensor activation to transduce and amplify immune signalling (Cesari, 2018; Saile et al., 2020). Recent studies have shown that activated TIR domains possess enzymatic activity and generate signalling molecules that activate EDS1-containing complexes. These complexes subsequently recruit the helper RNL families ADR1 and NRG1, which act downstream of TNL activation to promote immune signalling and, in some cases, cell death (Sun et al., 2021)

Depending on the N-terminal domain, NLRs are classically classified as TNLs (with a Toll-interleukin receptor, or TIR domain), CNLs (with a Coiled coil) or RNLs (with a RPW8, Resistance to Powdery Mildew 8). However, in recent years, numerous studies have revealed that NLRs are more diverse than previously thought and variable in both their structure and their mode of action and there is evidence of truncated versions of NLRs playing a functional role in biotic stress response. For example, the TIR-only protein RBA1 recognizes a *Pseudomonas syringae* effector and regulates cell death in *Arabidopsis* (Nishimura et al., 2017). It is still unknown how truncated NLRs remain inactive in the absence of a pathogen, considering that they lack the canonical domains usually involved in the control of NLR activity - the NB and/or the LRR (Cesari, 2018).

While NLRs are primarily associated with biotic stress responses, emerging evidence suggests their involvement in abiotic stress tolerance (Ariga et al., 2017; Rizwan et al., 2023). Van Ghelder et al. (2019) explored the response of conifer NLR genes to drought stress. Their analysis revealed that different groups of RNLs exhibited varying responsiveness to drought conditions. Notably, certain RNLs demonstrated significant overexpression in response to drought. In other studies, some RNL proteins like Activated Disease Resistance 1 (ADR1) were required for the signaling of three different Arabidopsis NLRs recognizing effectors. Interestingly, constitutive or conditional enhanced expression of ADR1 conferred significant drought resistance in *Arabidopsis* (Chini et al., 2004). ADR1 is therefore not only associated with drought-responsive phenotypes but is also a well-established “helper” RNL acting downstream of TNL-mediated immunity. Its expression under abiotic stress may reflect crosstalk or constitutive immune priming rather than a direct drought-specific recognition mechanism (Chini et al., 2004).

The objective of this study was to identify and characterize the predicted repertoire of NLR sequences in the cork oak genome, and investigate their potential roles in the response to environmental stress. Both canonical and non-canonical NLRs were identified, encompassing diverse domain architectures containing at least the NB-ARC domain. To infer their potential function we examined gene expression patterns across different cork oak tissues from plants exposed to drought conditions or infected with *Phytophthora cinnamomi*. Finally, using RNA-seq data, we explored the evolutionary dynamics of the NLR repertoire by analysing gene structure and the genetic diversity in two Iberian cork oak populations.

## Results

### Benchmarking InterNLR

The development of InterNLR was motivated by the need for a transparent and reproducible tool to identify canonical and non-canonical NLRs, while considering both domain composition and domain order. To elucidate about its performance and rigor, we compared it directly with DRAGO3 (Calle García et al., 2022), an already well-established tool to identify and classify plant-resistance genes, including NLRs, based on protein sequences. InterNLR was evaluated using the predicted proteome of *Quercus robur* (GCA_932294415.1; Plomion et al., 2018), to minimize fragmentation-induced annotation bias, as its genome assembly has achieved high contiguity at the scaffold level. We identified and classified a total of 358 sequences as canonical NLRs (CNLs, TNLs and RNLs), while with DRAGO3 this number raised to 410 (Supplementary Tables S1 and S2). A total of 343 amino acid sequences were shared between InterNLR and DRAGO3 classifications. From these, 339 were exact agreements and 4 were discordant, corresponding to an exact agreement rate of 98.8%. The 4 discordant sequences were classified as RNLs by InterNLR and CNLs by DRAGO3. DRAGO3 required 6:33.59 (mm:ss) time while InterNLR required 1:27:36 (h:mm:ss) for the same 10,548 KiB protein file (Supplementary Table S3). InterNLR required 4.34 logical CPU cores/threads (percent of CPU the job got = 434%) with a peak RAM usage of 4.75 GB while DRAGO3 required one CPU core for data uploading to PRGDB (percent of CPU the job got = 0%) and reached a peak RAM usage of 0.0234 GiB.

InterNLR detected 16 of the 19 Arabidopsis PRGdb-classified NLRs, corresponding to a sensitivity of 84.21%, while all 42 non-NLR proteins were correctly rejected, resulting in 100% specificity. Precision and overall accuracy were 100% and 95.08%, respectively. Among the 16 detected NLRs, 11 (68.75%) showed exact architecture agreement with the PRGDB classification. (Supplementary Table S5). InterNLR appears as an alternative candidate for (1) not only identifying but also detecting the exact order of the conserved domains, (2) detecting RNLs and (3) identifying non-canonical NLRs.

### Identification and characterization of NLRs in cork oak and related species

Understanding the diversity of nucleotide-binding leucine-rich repeat receptors (NLRs) is essential to further explore their roles in plant immune response. For the identification of the cork oak NLR gene family we ran InterNLR, identifying a total of 374 canonical NLRs in cork oak - 163 TNLs, 193 CNLs, and 18 RNLs. The ratio “number of RNLs” to “number of TNLs” stood at 0.1104. The same procedure was repeated for translated CDS sequences from *Quercus lobata* and *Quercus robur* to further explore the applicability of InterNLR. A total of 408 canonical NLRs were identified for *Q. lobata* - 199 TNLs, 197 CNLs, and 12 RNLs - and 358 canonical NLRs for *Q. robur* - 165 TNLs, 177 CNLs and 16 RNLs. The ratio “number of RNLs” to “number of TNLs” was 0.0603 and 0.0969 for *Q. lobata* and *Q. robur*, respectively.

We conducted a maximum likelihood phylogenetic analysis based on the NB-ARC domain of all cork oak canonical NLRs (Figure 1). We then expanded this analysis to include NLRs from all three *Quercus* species (Supplementary Figure S1). Specifically looking at cork oak NLRs, the phylogenetic study revealed a clear and distinct separation of CNL, TNL, and RNL clades. However, we did observe two NLRs that showed a discordant placement according to their predicted family, namely, LOC111989151 (CNL in the RNL cluster) and LOC112034384 (TNL in the CNL cluster). This result was consistent in the phylogenetic analysis of NLRs from the three *Quercus* species, using the conserved NB-ARC domain (Supplementary Figure S1).

**FIGURE 1:**
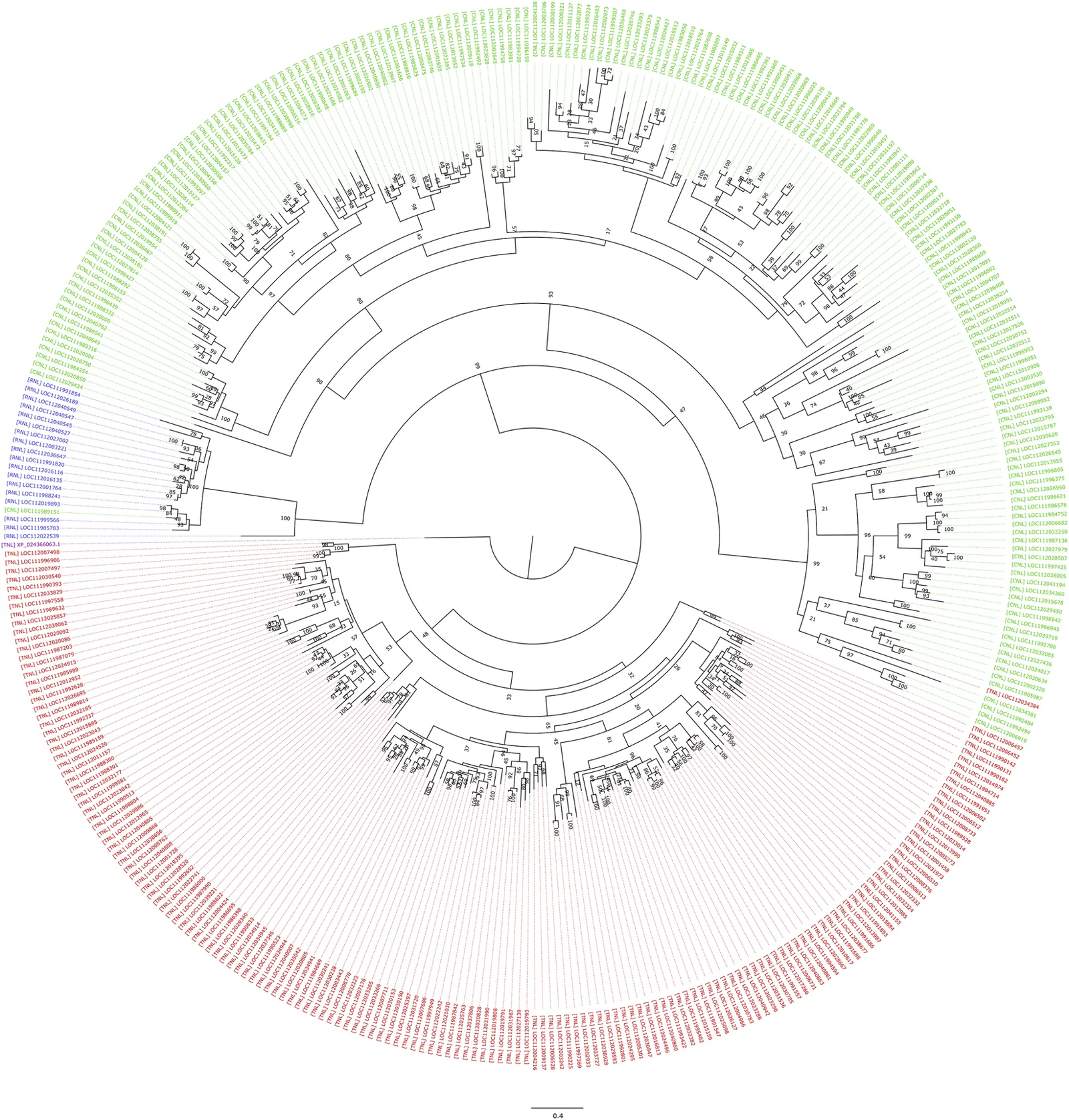
Maximum Likelihood Phylogeny of cork oak NLRs based on NB-ARC domain. Colors indicate NLR sub-groups: Red–TNLs; Green–CNLs; Blue–RNLs. The NB-ARC domain of *Physcomitrium patens* PRG gene was used as outgroup (purple). It is possible to distinct the polarization of each of CNL, TNL and RNL clades. The phylogenetic tree scale represents substitutions per site. Branch support values were generated from bootstrap analysis (1000 replications).

To elucidate the gene duplication patterns within cork oak NLRs as compared to those of *Q. lobata* and *Q. robur*, we employed OrthoFinder (Emms and Kelly, 2019) to conduct a separate analysis of the RNL clade (Supplementary Table S6), as RNLs form a well-supported clade in the canonical NLR phylogeny (Supplementary Figure S1). Phylogenetic analysis based on full-length amino acid sequences recovered a clade consistent with the duplication of RNL sequences in cork oak (Figure 2). This orthogroup (OG0000000) comprised nine *Q. suber* sequences, compared with six in *Q. lobata* and one in *Q. robur* (Supplementary Table S6). Within this orthogroup, sequences such as LOC112040545, LOC112016135 and LOC112016116 (NRG1) clustered together, supporting gene duplication within the NRG1 clade.

**FIGURE 2:**
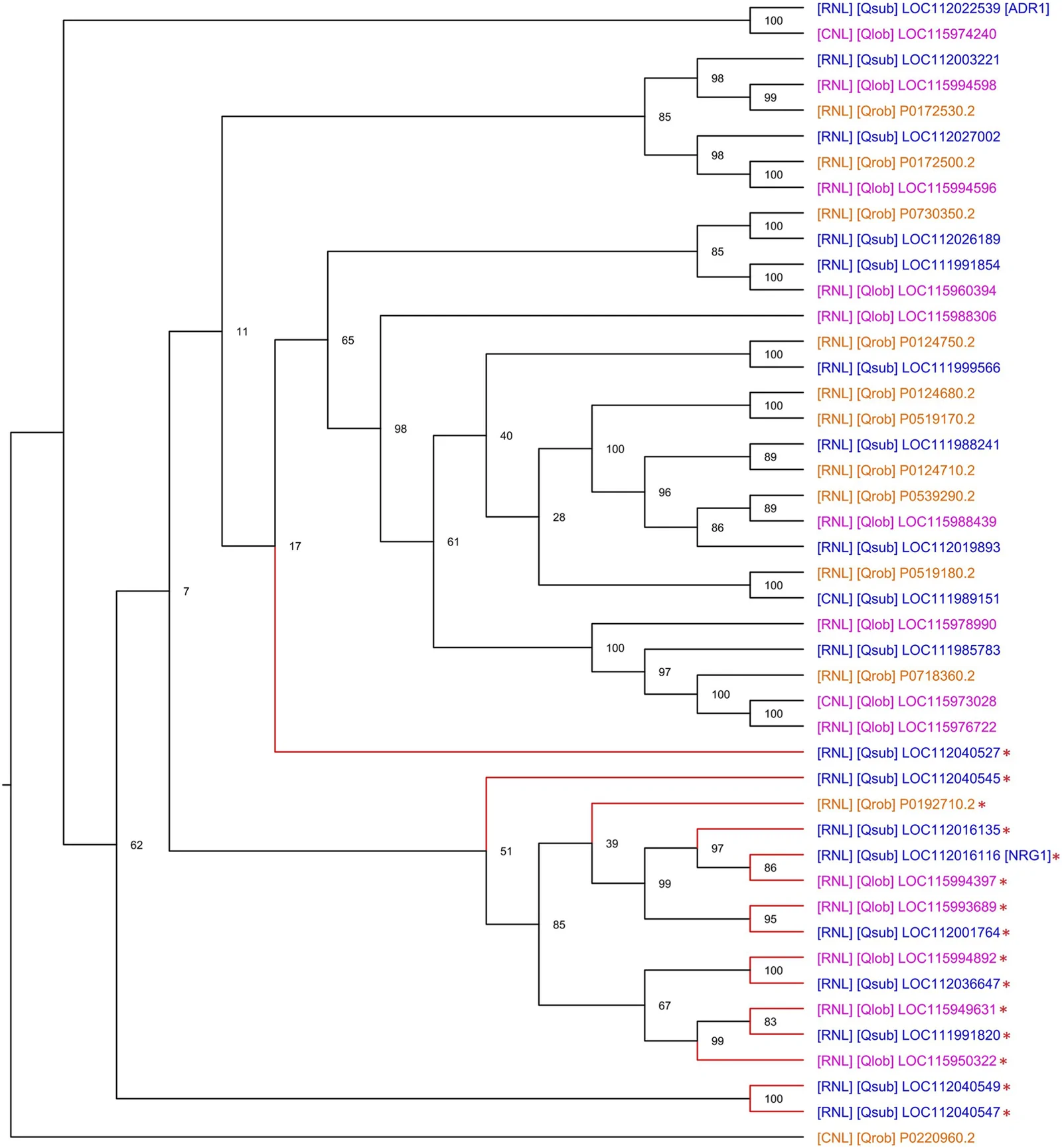
Maximum Likelihood phylogeny of *Q. suber, Q. lobata* and *Q. robur* NLRs from the RNL clade identified in Figure 1, based on their amino acid sequence. Tree leaves are coloured according to species: blue, *Q. suber*; magenta, *Q. lobata*; orange, *Q. robur*. The cladogram was rooted at midpoint. Red branches and red asterisks indicate genes belonging to the orthogroup (OG0000000, Supplementary Table S6) indentified in this study. Branch support values result from bootstrap analysis (1000 replications).

In addition to the traditional NLRs previously identified, our approach also uncovered NLRs with every possible domain combination and with at least the NB-ARC, broadening the spectrum of NLR gene diversity (Supplementary Table S7). This raised the total number of NLRs to 918 in cork oak. Alongside the canonical NLRs identified, we found 230 NLs (lacking an N-terminal domain), 143 Ns (lacking both N-terminal and C-terminal domains), 10 TCNLs (with TIR and CC domains), 1 TRNL (with TNL and RPW8 domains), 60 TNs (only with TIR and NB-ARC domains), 93 CNs (with CC and NB-ARC domains) and 7 RNs (RPW8 and NB-ARC domains only) (Supplementary Figure S2, panel a). These nomenclatures were attributed based on domain-architecture labels rather than as established phylogenetic subclasses. Phylogenetic analysis including all cork oak NLRs (canonical and non-canonical) again grouped the sequences in three main clades, identified as TNL-like, CNL-like, and RNL-like (Supplementary Figure S2, panel b). Ratios “number of TNLs” to “TNL-like sequences”, “CNLs” to “CNL-like sequences” and “RNLs” to “RNL-like sequences” were, respectively, 0.5, 0.350 and 0.45. A total of 780 and 867 canonical and non-canonical NLRs containing NB-ARC domain were identified with InterNLR in *Quercus robur* and *Quercus lobata* predicted proteomes, respectively, without considering splicing variants.

### Drought-responsive NLR expression profiles in cork oak stems

To identify candidate NLR genes with a potential role in abiotic stress response in cork oak stems, we used a publicly available RNA-seq dataset obtained from outer bark (phellem), phloem and cortex (inner bark) and xylem from 1.5 year-old plants (Barros et al., 2024). This dataset included plants grown under well-watered (WW) and water-deficit (WD, controlled dehydration for 2 months followed by 4 months at 10% relative water content) conditions, with three replicates each, which allowed us to assess transcriptional dynamics of NLRs in response to drought.

We first compared the average expression levels of the three canonical NLR gene classes with active expression in stem tissues. Kruskal–Wallis test revealed statistically significant differences between canonical and non-canonical NLR classes for all three tissues. Dunn’s post-hoc comparisons revealed a consistent pattern across stem tissues, with the N class showing significantly lower expression values than CN, CNL, NL, and TNL, while CNL also differed significantly from NL (Figure 3; Supplementary Table S8). The N class additionally showed lower expression than TCNL and TN in phellem and inner bark, and lower expression than RNL in xylem. A further significant difference between CNL and TN was detected in xylem (Figure 3; Supplementary Table S8). No significant differences were detected between RNLs and either CNLs or TNLs.

**FIGURE 3:**
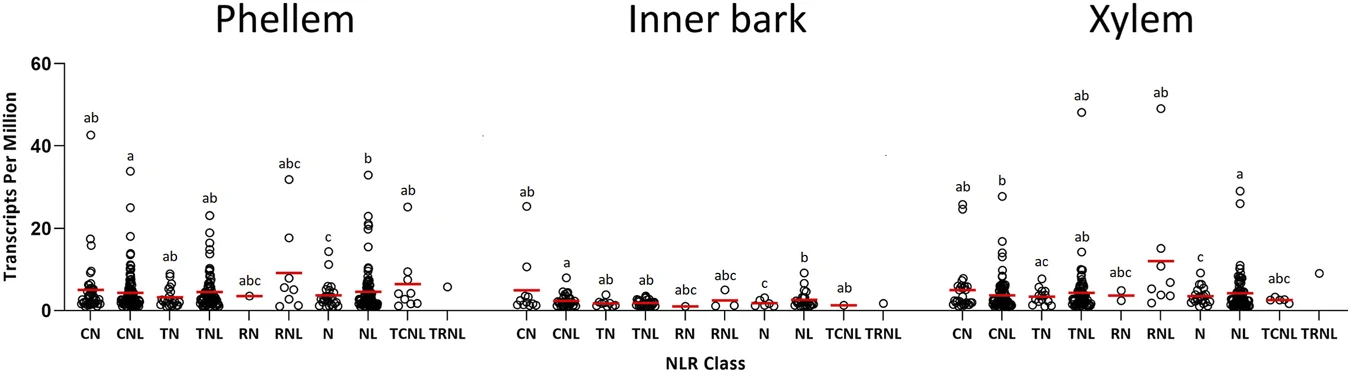
Dot plot representing the gene expression values (TPM) grouped according to NLR class and stem tissue. Red lines represent the mean TPM value. Different lowercase letters indicate a statistically significant difference (p < 0.05) as determined by Kruskal–Wallis test followed by a Dunn’s post-hoc test with Bonferroni correction for log_2_(TPM+1) transformed values. Groups that share at least one letter are not significantly different.

Additionally, there was a higher proportion of transcribed canonical NLRs compared to non-canonical NLRs (Supplementary Table S9). The top five NLRs with the highest expression values are indicated in Table 1, with LOC112003710 (RPP13) showing the highest representation in phellem, while LOC112022539 (ADR1-L1) showed high expression levels in inner bark and xylem. To further assess tissue specificity of NLR genes we analyzed the diversity of differentially expressed NLRs between the three tissue layers, independently of the growth conditions. From the hierarchical clustering based on mean expression per biological replicates (Figure 4), we found: 39 canonical and non-canonical NLRs enriched in phellem (including 7 TNLs, 12 CNLs and no RNLs), with some of these also represented in inner bark; 26 canonical and non-canonical NLRs enriched in inner bark (including 5 TNLs, 5 CNLs and no RNLs); and 52 canonical and non-canonical NLRs more expressed in xylem (including 15 TNLs, 15 CNLs and 1 RNL).

**TABLE 1:** Top five *NLRs* with higher mean expression in phellem, inner bark and xylem collected from 1.5 year-old plants.

Gene name	NLR class	Mean expression*			*Arabidopsis* orthologue	E-value
		Phellem	Inner bark	Xylem		
LOC112003710	CN	(1) 42.62	31.91	25.75	AT3G46530 (RPP13)	1.14E-33
LOC112033293	CNL	(2) 33.82	19.65	13.05	AT3G14470	1.81E-84
LOC112022566	NL	(3) 32.92	3.23	1.06	AT5G17680	3.17E-113
LOC112022539	RNL	(4) 31.83	(1) 73.39	(1) 49.05	AT4G33300 (ADR1-L1)	0
LOC112002846	TCNL	(5) 25.11	5.24	2.53	AT4G11170	1.07E-130
LOC112036463	NL	19.81	(2) 41.32	(3) 29.01	AT3G14470	1.22E-114
LOC112036467	CNL	18.01	(3) 40.29	(4) 27.72	AT3G14470	1.53E-133
LOC111988332	CNL	10.01	(4) 36.47	16.80	AT3G14470	1.51E-127
LOC112029450	CNL	7.58	(5) 32.22	5.97	AT4G27190	1.52E-71
LOC112012965	TNL	18.88	24.21	(2) 48.11	AT5G17680	1.36E-154
LOC112001962	NL	22.90	28.10	(5) 25.94	AT1G50180	3.03E-170

* Numbers in parentheses indicate the ranking order for the top five for each tissue.

Expression values are represented in Transcripts Per Million. the corresponding *arabidopsis* orthologue gene obtained using blastp is also indicated.

**FIGURE 4:**
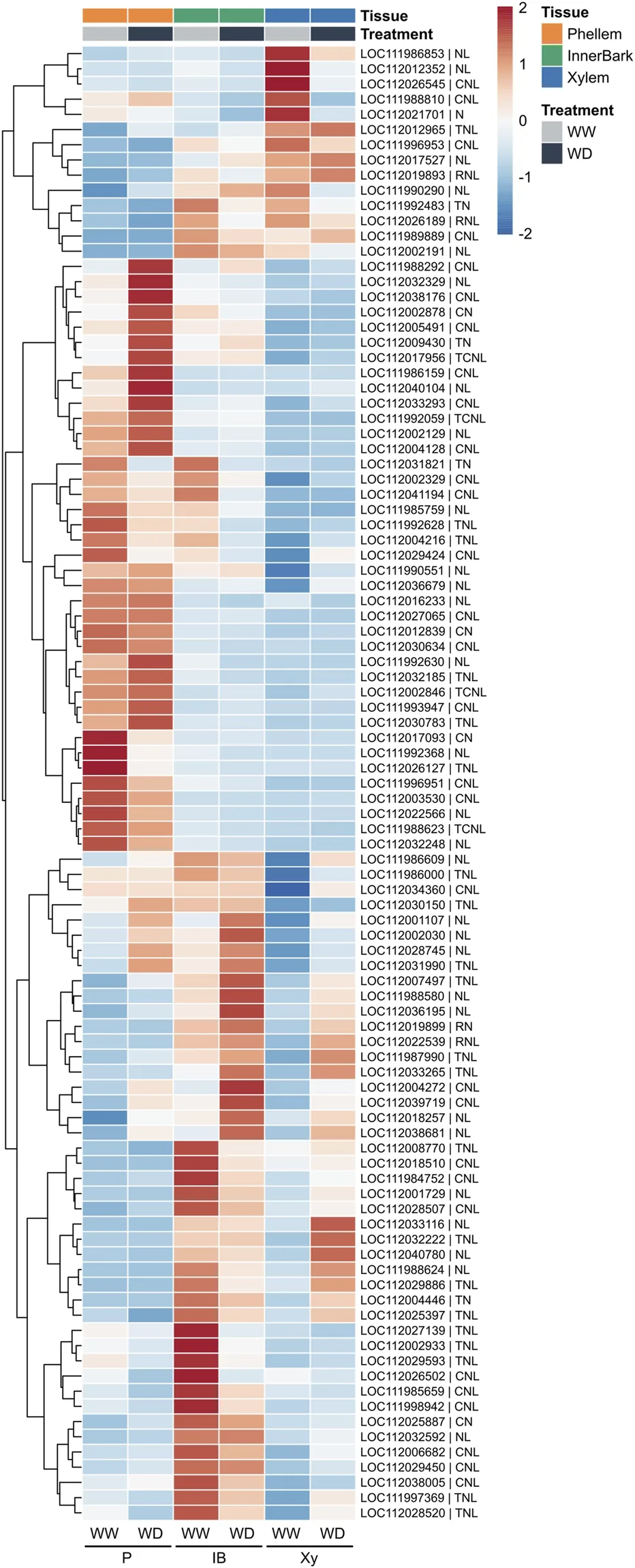
Gene expression heatmap for NLR genes differentially expressed between stem tissues from 1.5 year-old plants (Likelihood Ratio Test, adjusted p-value <0.001), independently of growth condition. Gene expression is represented by Z-scores of the TPM values to enable comparison of relative expression across genes and samples. WW, Well Watered; WD, Water Deficit; P, Phellem; IB, Inner Bark; Xy, Xylem. Genes were identified using the DESeq2 likelihood-ratio test, with Benjamini-Hochberg-adjusted p < 0.001. n = 97; 3 replicates per tissue.

To explore the relationship between NLR expression and adaptation to drought, we looked for NLR genes in the list of differentially expressed genes found in WD vs. WW comparison per tissue (Barros et al., 2024). We identified 35 differentially expressed (DE) NLRs in the phellem (Supplementary Figure S2, panel d1), 29 in the inner bark (Supplementary Figure S2, panel d2), and 97 in the xylem (Supplementary Figure S2, panel d3) (Supplementary Table S10). Among these DE NLRs, 9 genes found in both phellem and inner bark, 2 genes in both phellem and xylem, and one differentially expressed in both inner bark and xylem (Figure 5A). To gain deeper insights, we selected the differentially expressed NLRs for further analysis and TPMs were calculated for each tissue and condition (Figure 5B). A total of 25, 6 and 71 canonical and non-canonical NLRs were upregulated on drought stress, respectively, in phellem, inner bark and xylem tissues. A total of 10, 23 and 25 were downregulated, respectively, in the same three tissues.

**FIGURE 5:**
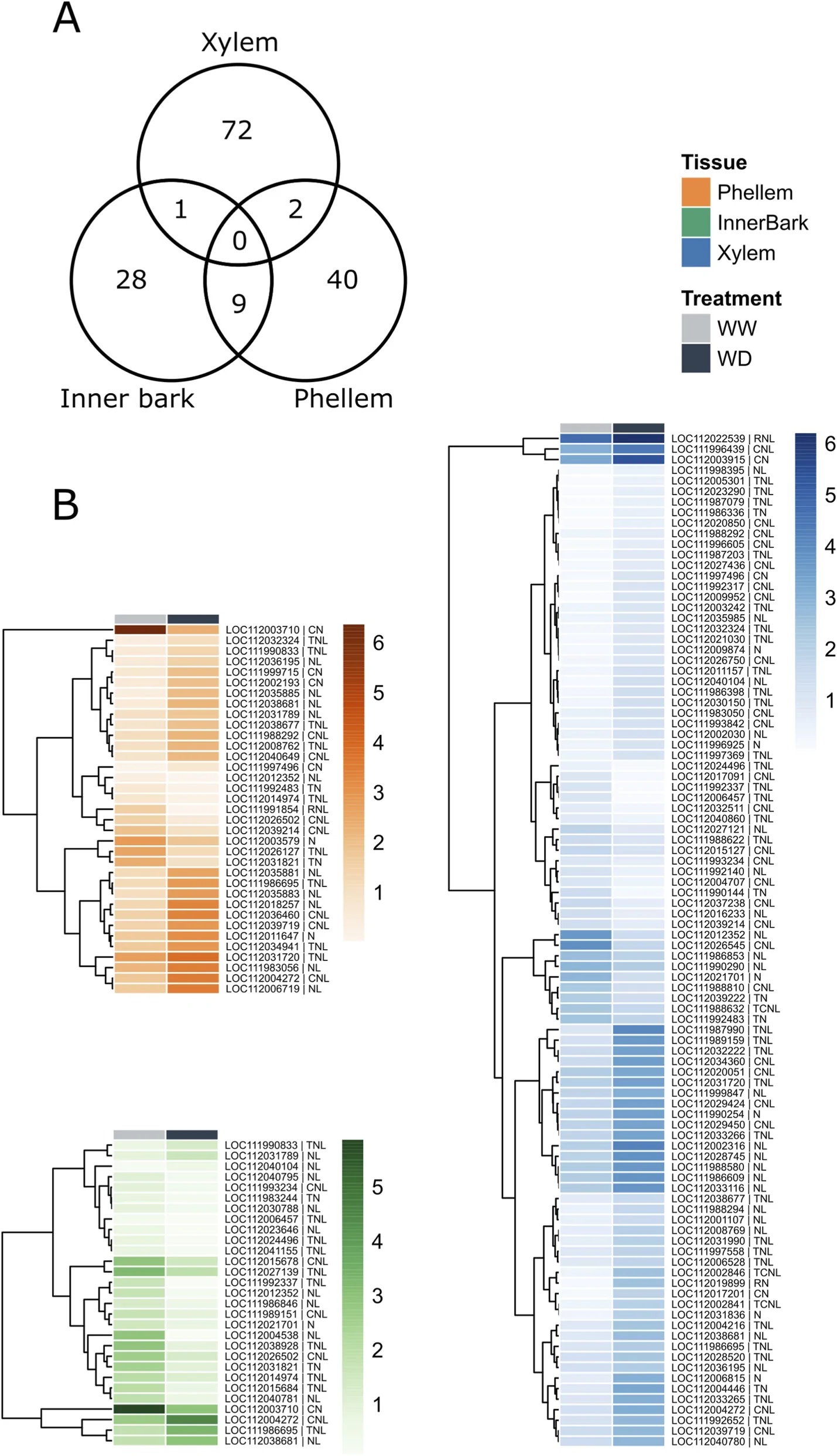
Differentially expressed canonical and non-canonical NLRs identified comparing WD vs. WW conditions in phellem, inner bark and xylem from cork oak 1.5-year-old stems. **(A)** Venn diagram of the intersection of the differentially expressed NLRs in phellem, inner bark, and xylem tissues. **(B)** Gene expression heatmaps for DE NLRs identified for phellem (n = 35), inner bark (n = 29) and xylem (n = 97); WW–Well watered, WD–Water Deficit; Gene expression is represented by log_2_(TPM+1) values.

To further explore the list of DE NLRs we analyzed their homology with NLRs from other plant species which have been reported as drought responsive. A total of 27 cork oak NLRs (from canonical and non-canonical groups) showed homology to NLRs from *Arabidopsis* (Ariga et al., 2017; Chini et al., 2004; Yang et al., 2021), *Arachis hypogea* (Rizwan et al., 2023), *Polygonatum kingianum* (Qian et al., 2021), *Oryza sativa* (Yang et al., 2022), *Nicotiana benthamiana* (Li et al., 2017) and *Picea glauca* (Van Ghelder et al., 2019), based on both best Blastp hit and OrthoFinder (Table 2; Supplementary Table S6). From these, 7 were differentially expressed genes in at least one of the three stem tissues (Supplementary Figure S2, panel c; Table 3). These were LOC112031821 (TN), LOC112006457 (TNL), LOC112021701 (N), LOC111996925 (N), LOC111996439 (CNL), RPM1 (LOC112040104, NL) and ADR1-like (LOC112022539, RNL).

**TABLE 2:** Cork oak orthologues of NLR genes from other plant species, previously reported to be regulated under with abiotic stress.

Gene name*	NLR class	Mean expression (TPM)		*Arabidopsis* homolog (Best blastp hit)	
		Stems	Roots	Gene name	E-value
LOC111990393	TNL	0.254	0.412	AT1G27170	0
LOC111987799	TN	0.882	0.000	AT5G17680	1.11E-116
LOC112035764	N	0.050	0.000	AT5G17680	1.63E-70
LOC112035762	N	0.256	0.000	AT5G17680	1.37E-71
**LOC112031821**	TN	1.925	0.485	AT5G17680	5.39E-135
LOC112034944	TNL	0.850	0.000	AT5G17680	9.60E-169
LOC112035366	NL	0.173	0.000	AT4G12010	1.71E-129
LOC111995402	TNL	0.585	1.666	AT5G17680	0
**LOC112006457**	TNL	0.276	1.416	AT5G36930	0
LOC112006452	TNL	6.185	1.122	AT5G36930	0
LOC111996235	N	0.168	0.000	AT5G36930	8.09E-81
LOC111996063	N	1.058	0.000	AT5G36930	3.93E-71
LOC111988305	TN	4.848	0.000	AT5G17680	1.91E-99
**LOC112021701**	N	2.665	9.388	AT1G59620 (CW9)	3.02E-09
LOC111992775	N	0.951	0.000	AT5G43730	1.68E-55
LOC111992786	CNL	3.188	0.927	AT1G12220 (RPS5)	2.04E-141
LOC112030634	CNL	1.972	0.632	AT4G27220	4.84E-94
**LOC111996925**	N	1.869	5.285	AT4G19060	4.08E-51
LOC111993947	CNL	2.091	1.161	AT3G14470	0
LOC112040068	N	1.131	0.786	AT3G14470	8.87E-87
LOC111988289	NL	0.633	0.346	AT3G14460	1.08E-61
LOC112035352	CNL	3.186	0.372	AT3G14470	3.64E-133
**LOC111996439**	CNL	15.816	2.748	AT3G14470	1.05E-136
LOC111990551	NL	16.099	14.267	AT1G50180	0
LOC111987192	NL	0.014	0.000	AT3G07040 (RPM1)	2.15E-142
**LOC112040104**	NL	3.116	1.213	AT3G07040 (RPM1)	9.22E-149
**LOC112022539**	RNL	51.428	45.665	AT4G33300 (ADR1-L1)	0

* Genes in bold are also differentially expressed in drought stress conditions.

The mean gene expression (in TPM) determined for cork oak stems (Barros et al., 2024) and cork oak roots (CorkOakDB) is indicated, along with the corresponding best Blastp hit in Arabidopsis.

**TABLE 3:** Selected cork oak NLR genes differentially expressed under drought stress in at least one of the three cork oak stem tissue layers.

Gene name	NLR class	WD vs. WW log_2_(FC)		
		Phellem	Inner bark	Xylem
LOC112031821	TN	−2.246	−2.196	–
LOC111996439	CNL	–	–	1.192
LOC112022539	RNL	–	–	1.206
LOC112006457	TNL	–	−2.884	−4.395
LOC112021701	N	–	−1.568	−2.162
LOC112040104	NL	–	2.605	3.160
LOC111996925	N	–	–	1.770

The table shows the corresponding log_2_ fold change (WD, vs. WW) for each gene. Genes were selected based on their homology to reference genes, previously reported in the literature as drought-responsive or associated with abiotic stress responses.

### Expression analysis of NLRs in cork oak roots infected with *P*. *cinnamomi*

In order to gain insights into the potential functions of the identified cork oak NLRs, we made use of a publicly available transcriptomic dataset, retrieved from CorkOakDB (Arias-Baldrich et al., 2020), consisting of RNA-seq data of roots on different developmental stages–roots from germinated acorns (days old) and thin white roots (2 months old) – both infected and non-infected with *P*. *cinnamomi* zoospores.

We identified 168 genes displaying a minimum Transcript Per Million (TPM) of 1 across at least one tissue/condition (Figure 6). Additionally, we computed the average TPM of the transcriptomic assay, unveiling that LOC112022539 (ADR1-L1 orthologue), exhibited the highest value among them (Table 4). Included among the top 10 highest average TPM values were genes like LOC111992494, LOC112003710 and LOC112017093, which are orthologous to *Arabidopsis* RPS2, RPP13 and RPM1, respectively. The five NLRs with the highest TPM-based log2 expression ratios between infected and non-infected conditions in each root type are presented in Table 5. From the 7 differentially expressed genes in at least one of the three stem tissues under WD vs. WW conditions, 4 of them also exhibit increased expression in roots infected with *P. cinnamomi*, LOC112021701 (N), LOC111996925 (N), LOC111996439 (CNL) and LOC112022539 (RNL, ADR1) (Table 6).

**FIGURE 6:**
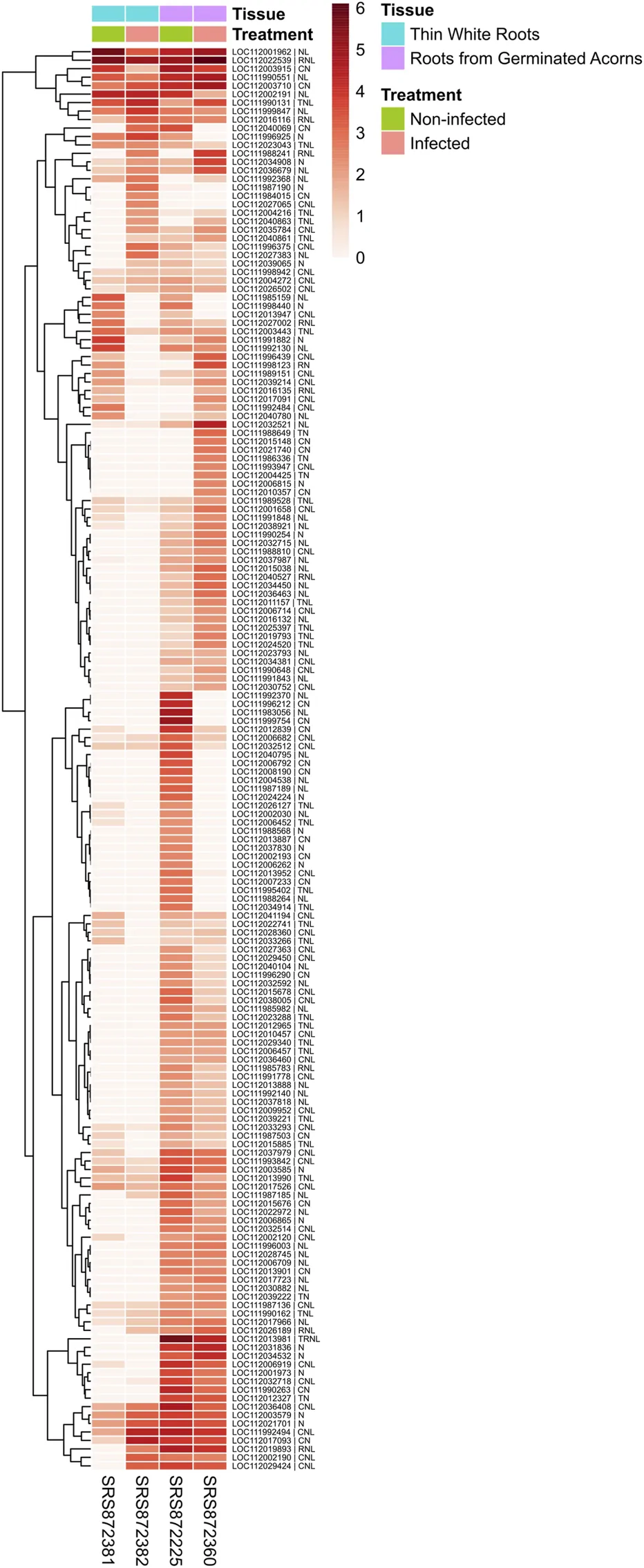
Gene expression heatmap for cork oak genes with strong expression in cork oak roots (TPM >1) in at least one development stage and in response to *P. cinnamomi* infection. The heatmap scale represents log_2_(TPM+1) values. n = 168.

**TABLE 4:** Top 10 NLRs with higher mean TPM values in cork oak roots infected with *P. cinnamomi* and control (non-infected) conditions.

Gene name	NLR class	Mean TPM	*Arabidopsis* homolog (best blastp hit)	
			Gene name	E-value
LOC112022539	RNL	45.665	AT4G33300 (ADR1-L1)	0
LOC112001962	NL	28.393	AT1G50180	3.03E-170
LOC112013981	TRNL	17.496	AT5G66900	4.15E-168
LOC111992494	CNL	15.214	AT4G26090 (RPS2)	1.01E-87
LOC112003710	CN	15.065	AT3G46530 (RPP13)	1.14E-33
LOC112002191	NL	14.517	AT3G14470	5.59E-111
LOC111990551	NL	14.267	AT1G50180	0
LOC112003915	CN	13.973	AT3G14470	5.89E-36
LOC112017093	CN	11.930	AT3G07040 (RPM1)	2.05E-158
LOC112019893	RNL	10.675	AT5G66900	0

**TABLE 5:** Cork oak NLR genes showing the top five highest fold-change–log_2_(FC) – in cork oak roots (from germinated acorns and thin white roots) infected with *P. cinnamomi* zoospores.

Gene name	NLR class	log_2_(FC)*		*Arabidopsis* homolog (best blastp hit)	
		Roots from germinated acorns	Thin white roots	Gene name	E-value
LOC111988241	RNL	(1) 3.841	2.686	AT5G66900	0
LOC111998123	RN	(2) 3.526	−2.109	AT5G66900	6.28E-39
LOC111988649	TN	(3) 3.093	0.000	AT5G36930	2.94E-66
LOC112021740	CN	(4) 2.781	0.000	AT3G14470	4.70E-75
LOC112015148	CN	(5) 2.733	0.000	AT3G14470	1.36E-84
LOC112017093	CN	−0.337	(1) 3.435	AT3G07040 (RPM1)	2.05E-158
LOC112002190	CNL	−0.172	(2) 3.269	AT3G14470	2.99E-103
LOC112029424	CNL	−0.413	(3) 3.220	AT3G50950 (ZAR1)	0
LOC112040069	CN	−3.495	(4) 2.997	AT3G14470	3.15E-85
LOC111996375	CNL	−0.876	(5) 2.978	AT4G27190	2.33E-76

* Log_2_((TPM_infected_+1)/(TPM_not-infected_+1)).

Fold change was calculated based on TPM, values from publicly available RNA-seq, data. Numbers enclosed in parentheses indicate the ranking order for the top five genes in each root types, based on log_2_(FC).

**TABLE 6:** Selected cork oak NLR genes differentially expressed under drought stress (in at least one of the three cork oak stem tissue layers) and with absolute log_2_(FC) > 0 for *P. cinnamomi* infection (in at least one root type).

Gene name	NLR class	WD vs. WW log_2_(FC)			Infected roots vs. non-infected log_2_(FC)	
		Phellem	Inner bark	Xylem	Roots from germinated acorns	Thin white roots
LOC112021701	N	–	−1.57	−2.16	−0.61	1.34
LOC111996925	N	–	–	1.77	−2.08	1.12
LOC111996439	CNL	–	–	1.19	2.33	−1.49
LOC112022539	RNL	–	–	1.21	1.04	−0.56

### Population diversity analysis of constitutively expressed NLRs

Addressing how NLRs behave in terms of genetic diversity is a step forward towards the understanding about their evolutionary dynamics. To evaluate genetic diversity of NLRs occurring in cork oak trees growing in the field, we used two independent RNASeq datasets obtained for individuals trees – 9 from Portugal (Population 1, Bioproject PRJEB33874, Lopes et al., 2020) and 11 from Spain (Population 2, Bioproject PRJNA650215, Fernández-Piñán et al., 2021). After read alignment against the reference genome, population diversity analysis was conducted using the information obtained for 564 NLRs (218 canonical and 346 non-canonical) displaying mean raw counts ≥10 reads across all the individuals. The final SNP filtering on the variant call file consisted on a depth of coverage ≥10 and a quality threshold ≥20. The weighted means for observed heterozygosity (H_O_), expected heterozygosity under Hardy-Weinberg Equilibrium (H_E_), nucleotide diversity (π) and Tajima’s D statistic were calculated for canonical and non-canonical NLRs for Portuguese (population 1) and Spanish (population 2) populations and having both populations into consideration (Table 7). Observed heterozygosity was lower than expected heterozygosity, and nucleotide diversity was low (order of magnitude of 10^–3^), for all cases. Tajima’s D weighted mean was positive, indicating a mean nucleotide diversity greater than the weight of the number of polymorphic sites (Watterson’s ϑ).

**TABLE 7:** Weighted mean of genetic diversity statistics across two Iberian cork oak populations, inferred based on commonly expressed genes from transcriptomic profiles of individual trees.

Statistics (weighted mean)	Population		
	Pop1 (PT)	Pop2 (ES)	Total (PT + ES)
H_O_	0.2737	0.2974	0.2368
H_E_	0.3788	0.3740	0.3115
π	0.0029	0.0016	0.0019
Tajima’s D	1.0253	1.0418	1.0875

Next, we identified the top 15 (expressed) NLR genes showing the highest and lowest Tajima’s D scores. The top genes with highest Tajima’s D ranged between 1.587 and 2.623 and included mostly NLRs containing CC domain (9 out of 15). The 15 NLRs with lowest Tajima’s D scores, ranging between −0.476 and −1.049 included canonical and non-canonical NLRs from different groups. The 15 genes with the highest Tajima’s D showed greater absolute values than the 15 with the lowest (Table 8). Also, we identified NLRs with positive and negative Tajima’s D values, showing heterogeneous allele frequency patterns among expressed NLR loci (Supplementary Table S12; Supplementary Figure S3). Furthermore, both groups of genes with top 15 highest and lowest Tajima’s D values include genes that are differentially expressed in response to drought - two being upregulated in the top 15 with highest values, and another four being two down- and two upregulated in the top 15 with lowest values.

**TABLE 8:** Top 15 NLRs with highest and lowest Tajima’s D values.

LOC ID	NLR type	Tajima's D
LOC111990646	CNL	2.623
LOC112027002	RNL	2.338
LOC111990643	CNL	2.179
LOC111992786	CNL	1.914
LOC112031742	N	1.907
LOC112017196	N	1.904
LOC112003221	RNL	1.867
LOC112011137	CNL	1.849
LOC112040625	CN	1.734
LOC111986853	NL	1.709
LOC111986155	CNL	1.677
LOC112018510	CNL	1.672
LOC112003746	CNL	1.611
LOC112017093	CN	1.606
LOC112004425	TN	1.587
LOC111993966	NL	-0.476
LOC112004336	NL	-0.476
LOC112005273	TNL	-0.488
LOC112033266	TNL	-0.531

LOC112040007	TNL	-0.588
LOC111993234	CNL	-0.601
LOC112004216	TNL	-0.605
LOC112017526	CNL	-0.652
LOC112026189	RNL	-0.690
LOC112040545	RNL	-0.698
LOC112006502	TNL	-0.757
LOC112004617	NL	-0.960
LOC112008190	CN	-0.967

LOC112020850	CNL	-1.007
LOC112002030	NL	-1.049

Blue entries with light grey shading represent upregulated genes in drought stress. Red entries with dark grey shading represent downregulated genes.

Principal Component Analysis (PCA) based on NLR data explained 9.86% of the variance in PC1 (Figure 7). This analysis revealed a clear separation of Population 2 accessions from Population 1 ones, although some degree of admixture was still evident between the two populations. Population 1 shows a more diffuse distribution in principal component space, indicating a weaker structure than Population 2, which forms a tighter cluster.

**FIGURE 7:**
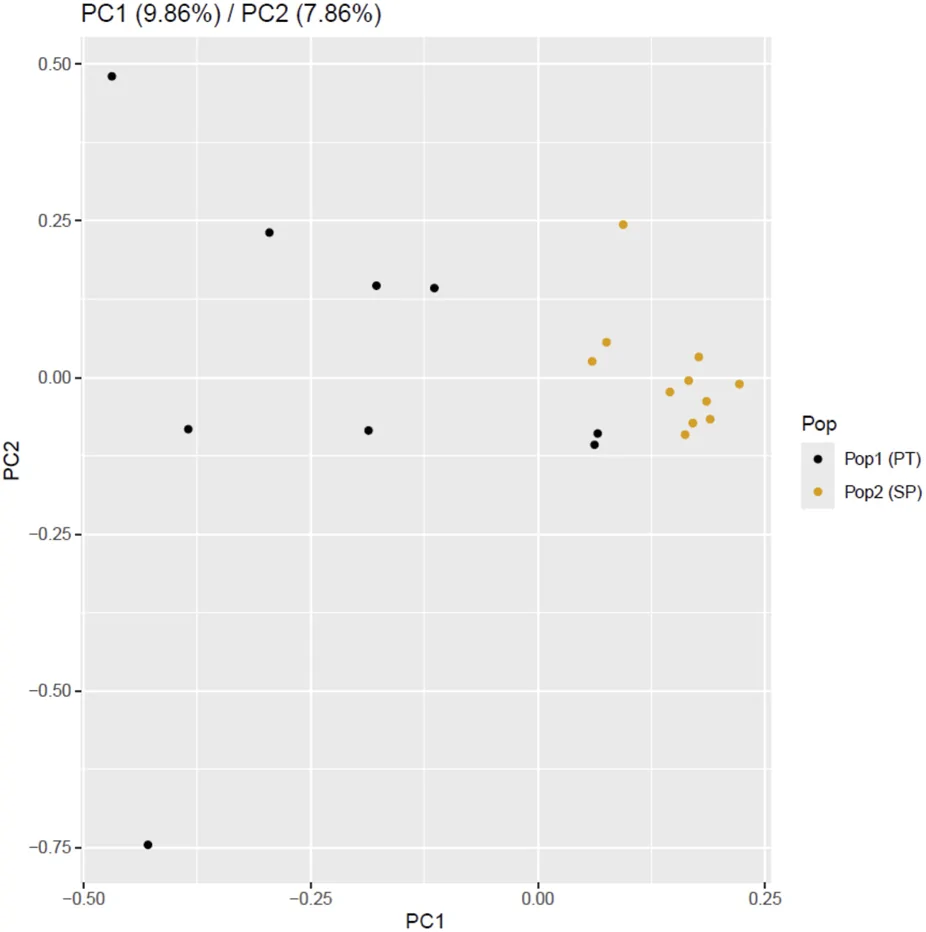
Principal Component Analysis based on genotype likelihood of the genomic regions corresponding to NLRs for the Iberian populations.

## Discussion

### Exploring NLR repertoire using InterNLR

In order to fully characterize the NLR-ome of cork oak we sought to identify both canonical and non-canonical NLRs. While DRAGO3 could provide this identification, this is limited to truncated forms and would not include RNLs. This prompted us to develop InterNLR, to guarantee a comprehensive assessment of all classes, considering also the ordering of the conserved domains. We further benchmarked InterNLR against DRAGO3 using the predicted proteome of *Quercus robur*, because its genome assembly has already achieved scaffold-level contiguity, providing a more complete dataset for evaluation. Although this comparison does not represent an exhaustive benchmark against all available NLR annotation tools, it provides an independent assessment of InterNLR performance and classification consistency. The two tools detected 343 shared candidates, of which 339 received the same classification, corresponding to an exact agreement rate of 98.8%. This high agreement indicates that InterNLR produces classifications that are largely consistent with those of an established resistance-gene annotation tool. The four discordant candidates were classified as RNLs by InterNLR and as CNLs by DRAGO3. This difference likely reflects the explicit distinction made by InterNLR between the RPW8-like coiled-coil (CC) domain characteristic of RNLs and the canonical CC domain of CNLs.

DRAGO3 identified more canonical NLR candidates than InterNLR, including 67 candidates not identified by InterNLR, whereas 15 candidates were identified exclusively by InterNLR. These differences may reflect: (i) the filtering performed by InterNLR to comply with the conserved domain order (N terminal > NB-ARC > LRR); and (ii) the different databases underlying each tool for domain annotation. Runtime, peak memory and processor usages were recorded on both workflows, but those could not be directly compared because DRAGO3 performs its main analysis remotely on the PRGDB server, while InterNLR runs locally.

Against the PRGdb reference set, InterNLR achieved 84.21% sensitivity, 100% specificity and precision, and 95.08% accuracy. Exact architecture agreement was lower (68.75% among detected NLRs), showing that NLR detection and classification are distinct performance levels. Together with the 98.8% concordance with DRAGO3 among shared hits, these results support the consistency of InterNLR.

### Cork oak immunity is anchored in RNL-related duplication and NLR diversification

The maximum likelihood phylogenetic analysis that was conducted with the NB-ARC sequences of both canonical and non-canonical NLRs revealed distinct clades, which emphasizes the high conservation of the NB-ARC domain, allowing to generate clade polarization based on the N-terminal region. The fact that two NLRs were misplaced outside their predicted family may reflect discordance between full domain-architecture classification and NB-ARC-based phylogenetic placement. The RNL clade displayed a well-conserved and polarized pattern compared to the other clades. This observation aligns with the proposal that RNLs primarily function as NLR “helpers”, exhibiting a higher degree of conservation, while “sensor” NLRs are subject to more mutational events driven by selection pressure from new pathogen adaptations (Cesari, 2018). This selective pressure contributes to “birth” and “death” events within the NLR clades, leading to the existence of truncated and non-canonical forms. Notably, within each clade, NLRs with intact N-terminal regions tend to be located adjacently, such as CNs next to CNLs. Comparing with the other two canonical NLR classes, the lower ratio of “number of CNLs” to “CNL-like genes” suggests a potentially more dynamic gene turnover process within this class, resembling a scenario consistent with birth and death evolutionary dynamics (Jacob et al., 2013). Both the phylogenetic analyses of the three *Quercus* species–considering all canonical NLRs and the RNL clade separately–and the OrthoFinder results support the hypothesis of gene duplication within the RNL clade. The ratios can vary significantly among plant species, ranging from as low as 0.04 in *Solanum tuberosum* to as high as 0.38 in *Vitis vinifera* (Shao et al., 2016). In conifers species, this ratio can reach 0.10 (Van Ghelder et al., 2019). Still, given the low absolute number of RNLs, we cannot exclude the possibility that the observed differences reflect uncertainties in the upstream annotation of the *Quercus* proteomes rather than true duplication events or meaningful variation in the number of RNLs” to “number of TNLs” ratio. Nevertheless, it is noteworthy that all RNL genes within the orthogroup displaying gene duplications share orthology with AT5G66900 (N Requirement Gene 1, NRG1) in *Arabidopsis*. NRG1, a subfamily of RNLs closely related to ADR1 in *Arabidopsis*, serves as a “helper”, as demonstrated by Castel et al. (2019). It plays an important role by facilitating the hypersensitive cell death response, ensuring complete resistance against oomycete pathogens in *Arabidopsis*. This study revealed that the involvement of NRG1 in bacterial pathogen resistance in *Arabidopsis* is not essential since its function is shared with ADR1 (Castel et al., 2019). Therefore, examining both the regulation and the expression patterns of NRG1 orthologues in response to biotic stresses will deepen our understanding of their roles in plant defence mechanisms.

### Expression profiles unveil functional and evolutionary layers of NLR diversity

Analyses of NLR gene expression in 1.5-year-old stems revealed distinct patterns, suggesting tissue-specific specialization. Differential analysis between tissues highlighted a greater diversity of DE NLRs with high expression in xylem compared with phellem and inner bark. The high diversity of NLRs expressed in the xylem may reflect the need for redundancy and collaboration among different receptor classes to secure this vital tissue. Since the xylem is essential for water and nutrient transport and is a primary target of vascular pathogens and environmental stresses, multiple NLR classes acting in concert could provide a broader recognition spectrum and a more robust immune response.

The significant global differences among NLR classes were therefore mainly driven by contrasts involving the N class, rather than by broad differentiation among all canonical and non-canonical classes. N proteins contain the NB-ARC domain but lack the additional domains that define the canonical CNL, TNL and RNL architectures. This structurally reduced, and potentially heterogeneous class may include partial genes, truncated proteins or independently retained proteins with NB-ARC domain, which could contribute to its distinct expression distribution. Nevertheless, expression differences alone do not establish whether these genes represent functional receptors, evolutionary intermediates or incomplete annotations.

In stems, LOC112003710, an orthologue of RPP13, stood out as the gene with highest expression gene in phellem. In *Arabidopsis*, this gene encodes a CNL. It is known for its ability to confer resistance to various strains of *Peronospora parasitica*, a biotrophic oomycete responsible for downy mildew (Bittner-Eddy et al., 2000). Interestingly, within cork oak, LOC112003710 lacks the typical C-terminal Leucine Rich Repeat (LRR) domain commonly associated with CNLs. However, previous research has suggested that an NLR without the LRR domain can still function as a guard and initiate defence response (Zhao et al., 2015). One gene that the highest expression in inner bark and xylem was LOC112022539, orthologue to *Arabidopsis* ADR1. Recent research suggests that ADR1 (and other RNLs) interact with the plasma membrane (PM) and activate hypersensitive response (Saile et al., 2021).

By analyzing the average percentage of expressed genes for each NLR class and the top five genes with higher mean expression values in each tissue, it becomes evident that the levels of expression of non-canonical NLRs cannot be negligible. While it may be difficult to ascertain specific functions based solely on expression assays, it is reasonable to infer that some of the NLRs may have or have had some function. As said before, these genes may be subjected to high selection pressure that comes from the pathogen adaptation, and this pressure result in a high degree of birth and death of NLRs (Monteiro and Nishimura, 2018). In this context, some non-canonical forms may represent remnants of formerly functional genes whose promoters remain active. Conversely, other forms may represent emerging NLRs in the process of acquiring function. Conducting a promoter profiling study would be crucial to further investigate and address this issue.

Previous studies have highlighted the presence of a basal level of expression for most NLRs, ensuring that the plant is prepared to counteract pathogen invasions that surpass the initial line of defence provided by PAMP-mediated triggered immunity (Jones et al., 2016). However, our investigation uncovers significant differential expression patterns of numerous NLRs under drought conditions in different tissues, including phellem, inner bark, and xylem. Particularly in the xylem, 97 canonical and non-canonical NLRs exhibited differential expression, indicating the critical role of this tissue in plant survival and adaptation to abiotic stresses.

The extensive literature review aiming to identify genes regulated by abiotic stress, together with the orthologue search within cork oak NLR-ome, opened way for a functional analysis of these genes. Out of the total 918 canonical and non-canonical NLRs, 27 were identified as orthologues to genes present in the list of abiotic stress-regulated genes identified in the literature (Supplementary Table S11). Among these, 7 displayed differential expression under drought conditions. From these genes, LOC112031821 (TN) is orthologue of the ACQOS locus, which includes AT5G46490, AT5G46500, AT5G46510, and AT5G46520. This locus has been previously identified as to be upregulated in response to osmotic stress in root tissues. However, our study demonstrated different results. We observed downregulation of this gene in phellem and inner bark under drought stress conditions. It is worth noting the differences in our assay compared to the study by Ariga et al. (2017), since we focused on stem tissues and induced drought stress, while their study focused on root tissues and osmotic stress. These differences may be sufficient for the variation on gene expression patterns observed. Furthermore, LOC111996925 (N) and LOC112006457 (TNL) are orthologous to AT5G45490 and AT5G36930, respectively. Intriguingly, all these genes, except LOC111996925, displayed downregulation in inner bark tissues. This pattern aligns with the findings of Yang et al. (2021), who reported that NLR genes, along with other genes induced by biotic stresses, are often repressed by abscisic acid, high temperatures, and drought. These results suggest that the transcriptional regulation of NLR genes may play a pivotal role in mediating the crosstalk between abiotic and biotic stress responses.

Another gene, LOC111996439 (CNL), displayed differential expression under water stress conditions, specifically showing upregulation in the xylem tissue. This gene is an orthologue to *Picea glauca* PG_003080_T.1 and *Arabidopsis* AT3G14470, encoding a putative disease resistance RPP13-like protein. Moreover, PG_003080_T.1 has been reported to be upregulated in response to drought, aligning with our findings and underscoring its potential role in abiotic stress response (Van Ghelder et al., 2019). Additionally, LOC112040104 (NL) emerged as another gene with differential expression patterns under water stress conditions, upregulated in both inner bark and xylem tissues. This gene is orthologue to *Arachis hypogaea* 0MH239.1 and WIN0ZV.1. Both have been previously reported to be upregulated in response to drought conditions (Rizwan et al., 2023). Moreover, it is orthologous to Arabidopsis RPM1, a CNL protein that confers resistance to *Pseudomonas syringae* that induces hypersensitive response (Boyes et al., 1998). These findings suggest the potential role for LOC112040104 (NL) in drought stress responses, possibly involving mechanisms similar to those observed in Arabidopsis RPM1-mediated resistance against bacterial pathogens.

Regarding the remaining gene from the seven with differential expression under drought conditions orthologues to abiotic stress-related genes identified in the literature, LOC112022539 (RNL), it shares orthology with *Arachis hypogaea* 3L0H24.1, which has been reported to exhibit upregulation in drought conditions (Rizwan et al., 2023). Similarly, *Arabidopsis* ADR1, an LOC112022539 orthologue, has been linked to enhanced drought tolerance when overexpressed in the plant (Chini et al., 2004). Our findings align with these observations, as LOC112022539 also shows upregulation in xylem tissues during drought conditions. This consistency in upregulation across different plant species, including the orthologue of this gene in *Abies alba*, reinforces the potential significance of LOC112022539 in mediating drought responses at xylem level in cork oak (Behringer et al., 2015), thus playing an important role in edapho-climatic conditions favoring prolonged drought episodes.

### Four NLR candidates show concurrent expression patterns across drought and *P. cinnamomi* datasets

The analysis of the *P*. *cinnamomi* transcriptomic assay highlighted specific NLRs that may respond to this stimulus in root tissues (roots from germinating acorns and thin white roots from 2-month old plants). When analyzing the genes with higher mean expression values in this assay, LOC112022539 (RNL), the orthologue of Arabidopsis ADR1, was also highly expressed, suggesting that it can have a basal constitutive expression in roots. Two other genes, LOC112003710 (CN) and LOC112001962 (NL), orthologues to RPP13 and AT1G50180, respectively, displayed the same pattern, being highly expressed in both root and stem tissues, likely indicating constitutive expression. The highest expression difference between infected and non-infected conditions was observed for the cork oak RPM1 orthologue (LOC112017093, CN), in thin white roots. In *Arabidopsis*, this gene is required for hypersensitive response to *Pseudomonas syringae* infection (Boyes et al., 1998). Although, these are different biological systems, further research may validate the role of cork oak RPM1 upon *P. cinnamomi* infection.

Among the seven drought-responsive NLRs, four also showed concordant expression patterns in *P. cinnamomi* infected roots: LOC112021701 (N), LOC111996925 (N), LOC111996439 (CNL) and LOC112022539 (RNL, ADR1-L1 orthologue). These comparisons were not statistically tested and therefore do not establish differential expression in response to *P. cinnamomi* infection. Nevertheless, these responses across two distinct stress contexts, together with their orthology to abiotic stress-responsive NLRs described in other species, make them strong candidates for further investigation of a putative crosstalk between biotic and abiotic stress responses in cork oak. However, these expression patterns alone do not demonstrate a shared regulatory or functional mechanism.

### Genetic diversity and evolutionary dynamics of NLRs

The evolution of NLRs is shaped by the constant interplay between plant hosts and pathogens, leading to distinct evolutionary strategies (Stam et al., 2016). Yet, the evolutionary dynamics between pathogen effectors and host NLRs remains a subject of ongoing debate. The co-evolutionary Red Queen ‘Arms Race’ model states that the genomes of both pathogen and plant evolve to overcome defences or avoid infection, respectively, suggesting that positive selection acts on NLRs (Bakker et al., 2006). The alternative model – ‘Trench Warfare’ – predicts that there is long-term accumulation of genetic variation in the plant host that allows it to keep pace with parasite effector variations, suggesting balancing selection acting on NLRs (Stahl et al., 1999; Tellier et al., 2014).

Selection signatures can be elucidated through population genetic summary statistics calculated on NLR regions. The fact that we obtained a lower mean observed heterozygosity (H_O_) than mean expected under Hardy-Weinberg Equilibrium (H_E_, assuming a population that is randomly mating, with no selection, migration, or genetic drift) may indicate a deviation from neutrality. Although this pattern is commonly associated with purifying selection or inbreeding, it can also indicate the action of recent positive selection. In such cases, a selective sweep may drive a beneficial allele to high frequency or fixation, reducing genetic diversity and observed heterozygosity in the surrounding genomic region (Watterson, 1978; Payseur et al., 2002). Low mean nucleotide diversity (π), as observed, is also an indicator of positive selection (Noll et al., 2022) – when a beneficial allele rapidly increases in frequency, linked variation in the surrounding region is reduced, leading to a local decrease in genetic diversity.

In this study, the population-genetic analyses should be interpreted carefully. Because variants were called from RNA-seq rather than genomic DNA, the dataset is enriched for transcribed regions with expression in the sampled tissues, while low-frequency alleles and/or alleles present in genes with low expression may be underrepresented. Also, the limited sample sizes (nine Portuguese and eleven Spanish individuals) further reduce the precision of allele frequency estimates. Haplotype-specific expression, alternative splicing, uneven coverage across exons and possible RNA-editing events may also affect genotype calls and allele-frequency estimates. In addition, random read downsampling to approximate similar coverage across samples may introduce further uncertainty into estimates of heterozygosity, nucleotide diversity and Tajima’s D. For these reasons, the results describe patterns in the expressed fraction of the NLR repertoire and should be regarded as exploratory rather than as a complete representation of population-wide genomic variation.

Both mean observed heterozygosity and nucleotide diversity are relatively low, which may reflect the contribution of recent positive selection–consistent with the Red Queen ‘Arms Race’ model. However, this interpretation appears to contrast with the positive mean Tajima’s D observed in NLRs.

Tajima’s D evaluates the deviation between nucleotide diversity (π) and the number of segregating sites (Watterson’s θ) (Tajima 1989). When π exceeds θ, Tajima’s D becomes positive, often indicating balancing selection. Conversely, when π is lower than θ, Tajima’s D turns negative, which may suggest either purifying or recent positive selection (Carlson et al., 2005). However, population subdivision, demographic history and sampling structure may indicate similar selection patterns, so Tajima’s D test is not sufficient to validate any type of selection alone.

Per-gene Tajima’s D values were predominantly positive, with 91.41% of loci showing positive values and only 8.59% showing negative values. Although a small number of loci with particularly high positive values may have increased the overall mean, the positive trend was widespread across the analysed NLRs and was not restricted to a few outliers. Positive Tajima’s D values may be compatible with the maintenance of intermediate-frequency variants, as expected under balancing selection, but may also result from demographic processes or population structure. In contrast, the smaller group of loci with negative values may reflect recent positive selection or population expansion. The low nucleotide diversity and heterozygosity observed in some loci may also be consistent with selective sweeps, although these statistics alone do not demonstrate selection. Given that the estimates were obtained from RNA-seq-derived variants and are restricted to expressed NLR loci, these patterns should be interpreted as exploratory. Overall, the results suggest heterogeneous evolutionary histories among NLR loci, with a strong predominance of positive Tajima’s D values, rather than providing direct evidence that the Arms Race and Trench Warfare models are acting simultaneously.

Principal Component Analysis based on NLRs revealed a clear separation of the two Iberian populations, the Spanish (Population 2) and the Portuguese (Population 1), although with signs of admixture between them. This suggests that both genetic differentiation exists, and gene flow and/or shared ancestry may have contributed to the observed pattern. Therefore separation between Portuguese and Spanish individuals in the PCA indicates population structure, which may affect the allele-frequency spectrum, particularly when populations are analysed together, therefore affecting Tajima’s D values. Furthermore, the wider spread of the Portuguese population in the PCA space matches its higher nucleotide diversity. This is supported by a larger π obtained for the Portuguese population, suggesting higher pairwise genetic differences among individuals.

## Conclusion

In this study, we used InterNLR to identify and classify the NLR repertoire of cork oak, comprising 374 canonical NLRs and 544 non-canonical NLR proteins. Comparison with DRAGO3 showed a high level of classification agreement, supporting the consistency of the InterNLR as a canonical and non-canonical NLR proteins annotation tool. Phylogenetic analyses recovered the main NLR lineages and identified an RNL orthogroup with evidence of gene duplication, although the low number of RNLs and possible uncertainties in proteome annotation prevent broader conclusions regarding RNL expansion.

NLR expression varied among cork oak stem tissues and classes, with the main statistical differences involving the non-canonical N class. Seven NLRs were differentially expressed under drought and showed orthology to genes previously associated with abiotic stress in other plant species. Four of these–LOC112021701, LOC111996925, LOC111996439 and LOC112022539 – also showed similar expression patterns in roots infected with *P*. *cinnamomi*. These genes therefore represent candidates for further investigation on the crosstalk between biotic and abiotic stress responses, although their expression patterns do not necessarily demonstrate a shared molecular function.

Population-genetic analyses of RNA-seq-derived variants revealed differentiation between the Portuguese and Spanish populations and a strong predominance of positive Tajima’s D values among expressed NLR loci. However, because these estimates are restricted to the expressed fraction of the NLR repertoire and may be affected by demographic structure and RNA-seq-related biases, they should be interpreted as exploratory rather than as direct evidence for specific co-evolutionary models. Overall, this study provides a first integrated view of NLR diversity, expression and evolutionary variation in cork oak and identifies a set of candidate genes for future functional studies.

## Methods

### InterNLR benchmarking

InterNLR is a software tool developed in this study for the identification and classification of canonical and non-canonical NLRs from protein sequences. This Bash “wrapper” firstly uses Interproscan 5.51–85.0 (Zdobnov and Apweiler, 2001) analysis against ‘gene3d’, ‘pfam’ and ‘COILS’ databases, with default parameters and thresholds. After this, NLR domains are called by the Python script domain_caller.py, which creates a list of all domain matches and their respective protein coordinates. This list is then used as input for the Python script nlr_caller.py to obtain the final output, consisting of several plain text files containing the protein IDs according to each NLR type. The order of the domains is taken into consideration, and only proteins respecting the N-terminal/termini > NB-ARC > LRR order were classified as NLRs. Proteins that respect this order but lack at least one of the domains (except NB-ARC) were also considered. In the cases where both Coiled Coil and RPW8 were identified as N-terminal domains, RPW8 prevails for the classification. InterNLR is available on GitHub – https://github.com/lmgoncalves94/internlr.

Benchmarking was performed using the predicted protein sequences from *Quercus robur* (GCA_932294415.1, Plomion et al., 2018) as input for both InterNLR and DRAGO3 (Calle García et al., 2022), both with default parameters. Both software ran on the same computational conditions on Ubuntu 24.04.4 LTS (GNU/Linux 6.8.0–124-generic x86_64). Runtime, CPU thread usage and RAM peaks were measured with time -v command. The resulting protein classifications were compared between InterNLR and DRAGO3 only for canonical NLRs. Metrics such as number of protein sequences detected by InterNLR, DRAGO3 or both tools, and exact classification agreements and discordant classifications were measured. Exact agreement rate (EAR, %) was calculated according to the following formula: EAR = ([common hits with the same NLR class]/[common hits]) × 100.

To complement the DRAGO3 concordance analysis, binary NLR detection was evaluated using an *Arabidopsis* curated reference set obtained from PRGdb (https://prgdb.org/prgdb4/plants/?id=151, accessed 19-jul-2026). The dataset comprised 61 unique proteins: 19 proteins classified by PRGdb as NLRs (CNL, TNL, RNL, NL, TN, T or N) and 42 proteins classified as non-NLRs (RLK, RLP, LECRK, LYK, LYP or Others). PRGdb annotations were treated as the reference classifications. Any InterNLR classification other than “No hit” was considered a positive NLR detection. Reference NLRs detected or not detected by InterNLR were classified as true positives (TP) or false negatives (FN), respectively, whereas reference non-NLRs reported as “No hit” or assigned an NLR class were classified as true negatives (TN) or false positives (FP), respectively. Sensitivity was calculated as TP/(TP + FN) × 100, the false-negative rate as FN/(TP + FN) × 100, specificity as TN/(TN + FP) × 100, the false-positive rate as FP/(TN + FP) × 100, precision as TP/(TP + FP) × 100, and accuracy as (TP + TN)/(TP + TN + FP + FN) × 100. NLR architecture agreement was assessed separately: a detected reference NLR was considered an exact match only when the InterNLR and PRGdb class labels were identical (Supplementary Table S4).

### Phylogenetic analysis of NLR amino acid sequences

InterNLR v1.0 was run with predicted protein sequences from *Quercus suber* (assembly GCA_002906115.5, Usié et al., 2023), *Quercus lobata* (GCA_001633185.5, Sork et al., 2016) and *Quercus robur* (GCA_932294415.1, Plomion et al., 2018) as inputs, with default parameters. In case of genes with alternative splicing isoforms, the longest isoform was chosen as representative before classification.

The coordinates of the NB-ARC domain for each NLR were retrieved according to ‘gene3d’ and ‘pfam’ databases (from Interproscan 5.51–85.0) and Bedtools 2.18 (Quinlan and Hall, 2010) were used to obtain the peptide sequences corresponding to the NB-ARC domain of each NLR, both with default parameters. NB-ARC sequences were aligned using the MAFFT Version 7 GUI with default parameters (Katoh and Toh, 2008). The resulting alignment file was trimmed in TrimAl 1.3 (Capella-Gutiérrez et al., 2009) with a gap threshold of 0.9 and a minimum percentage of the positions in the original alignment to conserve of 0.6. The trimmed alignments were used as input for RAxML 8.0.0 (Stamatakis, 2014) to construct a maximum-likelihood phylogenetic tree, with bootstrap analysis (1000 replications), using default parameters. The consensus trees were edited and plotted using FigTree 1.3.0 (http://tree.bio.ed.ac.uk/software/figtree/). OrthoFinder v3.0.1b1 (Emms and Kelly, 2019) was used to identify orthogroups for the RNL clade using the full-length amino acid sequence, using default parameters. A phylogenetic tree based solely on the RNL clade from the three *Quercus* species was also inferred based on this data to support the OrthoFinder results.

### Gene expression analysis

The gene expression analysis started with an already existing dataset of RNA-seq raw read counts of three tissues of cork oak. The experimental design consisted on 1-year old plants that were maintained in two contrasting irrigation conditions for 6 months (March to August). Tissue-specific sampling was conducted by collecting 10 cm segments above the root collar, specifically targeting the xylem, inner bark, and phellem tissues. Raw read counts were retrieved from ArrayExpress (accession E-MTAB-13376, PRJEB66289). TPM values were calculated from raw read counts in the R environment, and genes with TPM ≥1 were considered expressed. Differential expression analysis between the three tissues (independently of growth conditions) and between WW and WD was conducted using DESeq2 1.50.2, as described in Barros et al. (2024).

The publicly available transcriptomic dataset consisting of RNA-seq data of roots on different developmental stages - roots from germinated acorns (days old) and thin white roots (2 months old) - both infected and non-infected with *Phytophthora cinnamomi* zoospores was retrieved from CorkOakDB (www.corkoakdb.org). Genes with TPM values below 1 were excluded, and a descriptive expression ratio between infected and non-infected conditions was calculated as log2((TPM_infected_+1)/(TPM_non-infected_+1)). Because this comparison was based on TPM values rather than a replicate-level statistical model, no formal differential-expression analysis was performed and no P values or FDR-adjusted P values were calculated. These ratios were therefore used only to describe relative expression patterns between conditions.

### Statistical analysis of gene expression per NLR class by tissue

For each tissue, mean TPM values from drought stress assay were transformed in log_2_(TPM+1) to reduce skewness and allow the inclusion of genes with zero expression. The statistical assumptions required for parametric comparisons among NLR classes were assessed separately for each tissue. Sample sizes for each NLR class were also recorded, and classes with <3 genes were not considered for statistical analysis. Normality of the model residuals was evaluated using the Shapiro-Wilk test, whereas homogeneity of variance among NLR classes was assessed using the Levene’s test. A significance threshold of p < 0.05 was used to identify violations of these assumptions. Because the assumptions of normality and homogeneity of variance were not fully met, differences in gene-level expression among NLR classes were assessed separately for each tissue using the Kruskal–Wallis test followed by Dunn’s post-hoc test (with Bonferroni correction). Differences were considered statistically significant at an adjusted p < 0.05.

### Variants call

Raw RNA-seq sequencing reads were downloaded and converted to FASTQ files using sra-toolkit (Leinonen et al., 2011) for each SRA from each of the two bioprojects–PRJEB33874 and PRJNA650215. Trimmomatic (Bolger et al., 2014) was used to remove adapters and low-quality (Q < 15) and small (read length <36 bp) reads. STAR v2.7.11b (Dobin et al., 2013) was run using modified parameters (twopassMode = Basic, alignIntronMax = 100,000) to map the sequencing reads to the reference genome and obtain BAM files. SAMtools 1.22.1 (Danecek et al., 2021) was used to remove low-quality mapped reads (q < 20). Mapped reads were arbitrary removed to normalize the coverage for each BAM file (mean target coverage around 20 X). After normalization, FeatureCounts (Liao et al., 2014) was ran to obtain the raw read counts per site. Only NLRs (canonical and non-canonical) with an average of at least 10 mean raw counts per gene were selected for future analysis, corresponding to constitutively expressed NLRs. Those genes were filtered with BEDtools 2.18, with default parameters, and BAM files with only those NLR regions were obtained. After filtering, Samtools 1.22.1 was run to sort and index each resulting BAM file, with default parameters.

To call variants within NLR regions from RNA-seq, BCFtools 1.22 (Li, 2011) was employed. Given that the analysis focused on constitutively expressed regions, we treated these as representative of the genomic sequence. Variants were filtered using quality thresholds (QUAL ≥20) and depth (DP ≥ 10) to ensure reliability.

### Genetic diversity and structure

To obtain the population genetics statistics, variability in all constitutive NLRs was analysed with VCFtools (Danecek et al., 2011), using--het option to obtain the observed and expected homozygosity. These were further used to obtain the weighted mean observed (H_O_) and expected heterozygosity (H_E_) under Hardy-Weinberg Equilibrium using an in-house custom python script that calculates these values based on the observed and expected homozygosity output obtained with VCFtools. Nucleotide diversity (π) and Tajima’s D were calculated in 1000 bp sliding windows with --window-pi 1000 and --TajimaD 1000 options, respectively. An in-house Python script was used to summarize VCFtools v0.1.17 window-based Tajima’s D outputs at gene level. For each expressed NLR gene, the script retrieved all valid 1000 bp windows located within the gene coordinates and calculated the arithmetic mean of their Tajima’s D values, excluding -nan values. The same procedure was done for nucleotide diversity (π). A second in-house Python script was used to merge the per-gene nucleotide diversity and Tajima’s D outputs into a single table containing all NLR genes analysed. For each NLR gene, the script retrieved the corresponding per-gene π and Tajima’s D values from the processed VCFtools outputs. Genes without valid estimates in either file were retained in the final table and reported as missing values.

ANGSD 0.940-stable (Korneliussen et al., 2014) was used to compute genotype likelihoods and allele frequencies for PCA analysis. The tool was run with the parameters: doCounts 1, -GL 1, -doMajorMinor 1, -doMaf 1, -skipTriallelic 1, -doGeno 32, and -doPost 1. ngsCovar (part of NGS Tools; Fumagalli et al., 2014) was used to compute the covariance matrix for principal component analysis (PCA) based on genotype posterior probabilities. The input genotype likelihood file (.geno), generated by ANGSD, included 20 individuals. Parameters included -call 0 to use genotype posterior probabilities directly, and -norm 0 to avoid normalization of the covariance matrix. The covariance matrix produced by ngsCovar was used as input to generate the principal component plots using the plotPCA.R script from the NGS Tools package (Fumagalli et al., 2014). The first two principal components (PC1 and PC2) were plotted, and individuals were grouped according to a population metadata file (.clst).

## Data Availability

The RNA-seq data analyzed in this study are publicly available under the following accession numbers: PRJEB66289, PRJEB33874, and PRJNA650215. All secondary data generated or analyzed during this study are included in this article and its Supplementary Material files. Custom Python scripts used in this study are available at https://github.com/lmgoncalves94/Goncalves_et_al_2026_NLRs/blob/main/python_scripts.ipynb. InterNLR is available on GitHub (https://github.com/lmgoncalves94/internlr).

## Supplementary material

Supplementary Figure S1 Maximum Likelihood Phylogeny of Q. suber, Q. lobata and Q. robur canonical NLRs based on the NB-ARC domain. Colors indicate NLR sub-groups: Red – TNLs; Green – CNLs; Blue – RNLs. The NB-ARC domain of *Physcomitrium patens* PRG gene was used as outgroup (purple). It is possible to distinct the polarization of each of CNL, TNL and RNL clades. The phylogenetic tree scale represents substitutions per site. Branch support values result from bootstrap analysis (1000 replications).

Supplementary Figure S2 Maximum Likelihood Phylogeny based on the NB-ARC domains of the 918 NLRs and overview of gene expression patterns across cork oak stem tissues. **(a)** NLR domain architecture at each phylogenetic position. Colours encode domain composition: black indicates NL proteins; red indicates the presence of a TIR domain; green indicates the presence of a CC domain; blue indicates the presence of an RPW8 domain; and mixed colours indicate combinations of these N-terminal domains, such as yellow for TCNL and magenta for RTNL. Lighter shades indicate truncated proteins lacking the LRR domain: TN, light red; CN, light green; and RN, light blue. N proteins, containing only the NB-ARC domain, are shown in grey. The *Physcomitrium patens* PRG outgroup is shown in purple. **(b)** Major NLR classes: RNL-like, blue; TNL-like, red; and CNL-like, green. **(c)** NLRs of interest, including orthologues of abiotic stress-induced NLRs identified in other plant species, shown in grey, and genes that are both orthologous to abiotic stress-induced NLRs and differentially expressed under drought stress, shown in red. **(d)** Differential expression patterns of NLRs under water-deficit conditions, expressed as log2 fold change, in d1 phellem, d2 inner bark, and d3 xylem. Negative log2 fold-change values are represented in blue and positive values in red, with colour intensity reflecting the magnitude of the change. **(e)** Overall mean expression, expressed as TPM, in the three stem tissues under well-watered (WW) and water deficit (WD) conditions: phellem (e1), well-watered - e1a, water-deficit - e1b; inner bark (e2), well-watered - e2a, water-deficit - e2b; and xylem (e3), well-watered - e3a, water-deficit - e3b. Differential expression between WD and WW conditions was tested separately for each tissue using the DESeq2 Wald test. Genes with |log_2_FC| > 1 and Benjamini-Hochberg-adjusted P < 0.05 were considered differentially expressed. The analysis included three biological replicates per condition. The phylogenetic tree scale represents amino acid substitutions per site. Branch support values result from bootstrap analysis (1000 replications). n = 918.

Supplementary Figure S3 Per-gene Tajima’s D values distribution in expressed NLR loci in Portuguese and Spanish *Quercus suber* populations.

## References

[B1] Arias-Baldrich C. Silva M. C. Bergeretti F. Chaves I. Miguel C. Saibo N. J. M. (2020). CorkOakDB-the cork oak genome database portal. Database 2020, 1–11. 10.1093/database/baaa114 33382885 PMC7774466

[B2] Ariga H. Katori T. Tsuchimatsu T. Hirase T. Tajima Y. Parker J. E. (2017). NLR locus-mediated trade-off between abiotic and biotic stress adaptation in *Arabidopsis* . Nat. Plants 3 (May), 17072. 10.1038/nplants.2017.72 28548656

[B3] Bakker E. G. Toomajian C. Kreitman M. Bergelson J. (2006). A genome-wide survey of R gene polymorphisms in *Arabidopsis* . Plant Cell. 18 (8), 1803–1818. 10.1105/tpc.106.042614 16798885 PMC1533970

[B4] Barros P. M. Sapeta H. Lucas D. A. Oliveira M. M. (2024). The impact of drought on phellem development: identification of novel gene regulators and evidence of photosynthetic activity in cork oak stems. Environ. Exp. Bot. 224 (February), 105785. 10.1016/j.envexpbot.2024.105785

[B63] Behringer D. Zimmermann H. Ziegenhagen B. Liepelt S. (2015). Differential gene expression reveals candidate genes for drought stress response in *Abies alba* (Pinaceae). PLoS ONE 10 (4), e0124564. 10.1371/journal.pone.0124564 25924061 PMC4414588

[B5] Besson C. K. Lobo-do-Vale R. Rodrigues M. L. Almeida P. Herd A. Grant O. M. (2014). Cork oak physiological responses to manipulated water availability in a mediterranean woodland. Agric. For. Meteorology 184, 230–242. 10.1016/j.agrformet.2013.10.004

[B6] Bittner-Eddy P. D. Crute I. R. Holub E. B. Beynon J. L. (2000). RPP13 is a simple locus in *Arabidopsis thaliana* for alleles that specify downy mildew resistance to different avirulence determinants in *Peronospora parasitica* . Plant J. 21 (2), 177–188. 10.1046/j.1365-313X.2000.00664.x 10743658

[B7] Bolger A. M. Lohse M. Usadel B. (2014). Trimmomatic: a flexible trimmer for illumina sequence data. Bioinformatics 30 (15), 2114–2120. 10.1093/bioinformatics/btu170 24695404 PMC4103590

[B62] Boyes D. C. Nam J. Dangl J. L. (1998). The Arabidopsis thaliana RPM1 disease resistance gene product is a peripheral plasma membrane protein that is degraded coincident with the hypersensitive response. Proc. Natl. Acad. Sci. USA 95 (26), 15849–15854. 10.1073/pnas.95.26.15849 9861059 PMC28133

[B8] Calle García J. Guadagno A. Paytuvi-Gallart A. Saera-Vila A. Amoroso C. G. D'Esposito D. (2022). PRGdb 4.0: an updated database dedicated to genes involved in plant disease resistance process. Nucleic Acids Research 50 (D1), D1483–D1490. 10.1093/nar/gkab1087 34850118 PMC8729912

[B59] Camilo-Alves C. S. P. Vaz M. Da Clara M. I. E. Ribeiro N. M. D. A. (2017). Chronic cork oak decline and water status: new insights. N. For. 48, 753–772. 10.1007/s11056-017-9595-3

[B9] Capella-Gutiérrez S. Silla-Martínez J. M. Gabaldón T. (2009). trimAl: a tool for automated alignment trimming in large-scale phylogenetic analyses. Bioinformatics 25 (15), 1972–1973. 10.1093/bioinformatics/btp348 19505945 PMC2712344

[B10] Carlson C. S. Thomas D. J. Eberle M. A. Swanson J. E. Livingston R. J. Rieder M. J. (2005). Genomic regions exhibiting positive selection identified from dense genotype data. Genome Res. 15 (11), 1553–1565. 10.1101/gr.4326505 16251465 PMC1310643

[B11] Castel B. Ngou P. M. Cevik V. Redkar A. Kim D. S. Yang Y. (2019). Diverse NLR immune receptors activate defence *via* the RPW8-NLR NRG1. New Phytol. 222 (2), 966–980. 10.1111/nph.15659 30582759

[B12] Cesari S. (2018). Multiple strategies for pathogen perception by plant immune receptors. New Phytol. 219 (1), 17–24. 10.1111/nph.14877 29131341

[B13] Chaves M. M. Maroco J. P. Pereira J. S. (2003). Understanding plant responses to drought - from genes to the whole plant. Funct. Plant Biol. 30 (3), 239–264. 10.1071/FP02076 32689007

[B14] Chini A. Grant J. J. Seki M. Shinozaki K. Loake G. J. (2004). Drought tolerance established by enhanced expression of the CC-NBS-LRR gene, ADR1, requires salicylic acid, EDS1 and ABI1. Plant J. 38 (5), 810–822. 10.1111/j.1365-313X.2004.02086.x 15144382

[B15] Cook B. I. Mankin J. S. Anchukaitis K. J. (2018). Climate change and drought: from past to future. Curr. Clim. Change Rep. 4 (2), 164–179. 10.1007/s40641-018-0093-2

[B16] Danecek P. Auton A. Abecasis G. Albers C. A. Banks E. DePristo M. A. (2011). The variant call format and VCFtools. Bioinformatics 27 (15), 2156–2158. 10.1093/bioinformatics/btr330 21653522 PMC3137218

[B17] Danecek P. Bonfield J. K. Liddle J. Marshall J. Ohan V. Pollard M. O. (2021). Twelve years of SAMtools and BCFtools. GigaScience 10 (2), 1–4. 10.1093/gigascience/giab008 33590861 PMC7931819

[B18] Dobin A. Davis C. A. Schlesinger F. Drenkow J. Zaleski C. Jha S. (2013). STAR: ultrafast universal RNA-seq aligner. Bioinformatics 29 (1), 15–21. 10.1093/bioinformatics/bts635 23104886 PMC3530905

[B19] Emms D. M. Kelly S. (2019). OrthoFinder: phylogenetic orthology inference for comparative genomics. Genome Biol. 20 (1), 1–14. 10.1186/s13059-019-1832-y 31727128 PMC6857279

[B20] Fernández-Piñán S. Boher P. Soler M. Figueras M. Serra O. (2021). Transcriptomic analysis of cork during seasonal growth highlights regulatory and developmental processes from phellogen to phellem formation. Sci. Rep. 11 (1), 1–14. 10.1038/s41598-021-90938-5 34103550 PMC8187341

[B21] Fumagalli M. Vieira F. G. Linderoth T. Nielsen R. (2014). NgsTools: methods for population genetics analyses from next-generation sequencing data. Bioinformatics 30 (10), 1486–1487. 10.1093/bioinformatics/btu041 24458950 PMC4016704

[B22] Hidalgo P. J. Marín J. M. Quijada J. Moreira J. M. (2008). A spatial distribution model of cork oak (*Quercus suber*) in southwestern Spain: a suitable tool for reforestation. For. Ecol. Manag. 255 (1), 25–34. 10.1016/j.foreco.2007.07.012

[B23] Jacob F. Vernaldi S. Maekawa T. (2013). Evolution and conservation of plant NLR functions. Front. Immunol. 4 (SEP), 1–16. 10.3389/fimmu.2013.00297 24093022 PMC3782705

[B24] Jones J. D. G. Vance R. E. Dangl J. L. (2016). Intracellular innate immune surveillance devices in plants and animals. Science 354 (6316), aaf6395. 10.1126/science.aaf6395 27934708

[B25] Korneliussen T. S. Albrechtsen A. Nielsen R. (2014). Angsd: analysis of next generation sequencing data. BMC Bioinforma. 15 (1), 1–13. 10.1186/s12859-014-0356-4 25420514 PMC4248462

[B66] Katoh K. Toh H. (2008). Recent developments in the MAFFT multiple sequence alignment program. Brief. Bioinform. 9 (4), 286–298. 10.1093/bib/bbn013 18372315

[B26] Leinonen R. Sugawara H. Shumway M. International Nucleotide Sequence Database Collaboration (2011). The sequence read archive. Nucleic Acids Res. 39 (Suppl. 1), 2010–2012. 10.1093/nar/gkq1019 21062823 PMC3013647

[B27] Li H. (2011). A statistical framework for SNP calling, mutation discovery, association mapping and population genetical parameter estimation from sequencing data. Bioinformatics 27 (21), 2987–2993. 10.1093/bioinformatics/btr509 21903627 PMC3198575

[B28] Li X. Zhang Y. Yin L. Lu J. (2017). Overexpression of pathogen-induced grapevine TIR-NB-LRR gene VaRGA1 enhances disease resistance and drought and salt tolerance in *Nicotiana benthamiana* . Protoplasma 254 (2), 957–969. 10.1007/s00709-016-1005-8 27468994

[B29] Liao Y. Smyth G. K. Shi W. (2014). FeatureCounts: an efficient general purpose program for assigning sequence reads to genomic features. Bioinformatics 30 (7), 923–930. 10.1093/bioinformatics/btt656 24227677

[B30] Lopes S. T. Sobral D. Costa B. Perdiguero P. Chaves I. Costa A. (2020). Phellem *versus* xylem: Genome-wide transcriptomic analysis reveals novel regulators of cork formation in cork oak. Tree Physiol. 40 (2), 129–141. 10.1093/treephys/tpz118 31860724

[B60] McDowell N. Pockman W. T. Allen C. D. Breshears D. D. Cobb N. Kolb T., (2008). Mechanisms of plant survival and mortality during drought: why do some plants survive while others succumb to drought?. New Phytologist 178, 719–739. 10.1111/j.1469-8137.2008.02436.x 18422905

[B32] Monteiro F. Nishimura M. T. (2018). Structural, functional, and genomic diversity of plant NLR proteins: an evolved resource for rational engineering of plant immunity. Annu. Rev. Phytopathology 56, 243–267. 10.1146/annurev-phyto-080417-045817 29949721

[B31] Moreira C. Huelva A. C. Maia I. Quartin V. Matos M. C. Cravador A. (2006). Studies on the association of the *Quercus suber* decline disease with *Phytophthora cinnamomi* in Portugal. Bol. Inf. CIDEU 1, 31–38. Available online at: https://sapientia.ualg.pt/handle/10400.1/1216 (Accessed January 2, 2025).

[B33] Nishimura M. T. Anderson R. G. Cherkis K. A. Law T. F. Liu Q. L. Machius M. (2017). TIR-only protein RBA1 recognizes a pathogen effector to regulate cell death in *Arabidopsis* . Proc. Natl. Acad. Sci. U. S. A. 114 (10), E2053–E2062. 10.1073/pnas.1620973114 28137883 PMC5347586

[B34] Noll D. Leon F. Brandt D. Pistorius P. Le Bohec C. Bonadonna F. (2022). Positive selection over the mitochondrial genome and its role in the diversification of gentoo penguins in response to adaptation in isolation. Sci. Rep. 12 (1), 1–13. 10.1038/s41598-022-07562-0 35260629 PMC8904570

[B35] Payseur B. A. Cutter A. D. Nachman M. W. (2002). Searching for evidence of positive selection in the human genome using patterns of microsatellite variability. Mol. Biol. Evol. 19 (7), 1143–1153. 10.1093/oxfordjournals.molbev.a004172 12082133

[B36] Pina-Martins F. Baptista J. Pappas G. Paulo O. S. (2019). New insights into adaptation and population structure of cork oak using genotyping by sequencing. Glob. Change Biol. 25 (1), 337–350. 10.1111/gcb.14497 30358018

[B37] Plomion C. Aury J. M. Amselem J. Leroy T. Murat F. Duplessis S. (2018). Oak genome reveals facets of long lifespan. Nat. Plants 4 (7), 440–452. 10.1038/s41477-018-0172-3 29915331 PMC6086335

[B38] Qian H. Xu Z. Cong K. Zhu X. Zhang L. Wang J. (2021). Transcriptomic responses to drought stress in *Polygonatum kingianum* tuber. BMC Plant Biol. 21 (1), 1–20. 10.1186/s12870-021-03297-8 34781887 PMC8591914

[B39] Quinlan A. R. Hall I. M. (2010). BEDTools: a flexible suite of utilities for comparing genomic features. Bioinformatics 26 (6), 841–842. 10.1093/bioinformatics/btq033 20110278 PMC2832824

[B40] Rizwan M. Haider S. Z. Bakar A. Rani S. Danial M. Sharma V. (2023). Evolution of NLR genes in genus *Arachis* reveals asymmetric expansion of NLRome in wild and domesticated tetraploid species. Sci. Rep. 13 (1), 1–10. 10.1038/s41598-023-36302-1 37291184 PMC10250334

[B41] Saile S. C. Jacob P. Castel B. Jubic L. M. Salas-Gonzáles I. Bäcker M. (2020). Two unequally redundant “helper” immune receptor families mediate *Arabidopsis thaliana* intracellular “sensor” immune receptor functions. PLoS Biol. 18 (9), 1–28. 10.1371/journal.pbio.3000783 32925907 PMC7514072

[B42] Saile S. C. Ackermann F. M. Sunil S. Keicher J. Bayless A. Bonardi V. (2021). *Arabidopsis* ADR1 helper NLR immune receptors localize and function at the plasma membrane in a phospholipid dependent manner. New Phytol. 232 (6), 2440–2456. 10.1111/nph.17788 34628646

[B61] Shao Z. Q. Xue J. Y. Wu P. Zhang Y. M. Wu Y. Hang Y. Y. (2016). Large-scale analyses of angiosperm nucleotide-binding site-leucine-rich repeat genes reveal three anciently diverged classes with distinct evolutionary patterns. J. Instrum. 170 (4), 2095–2109. 10.1104/pp.15.01487 26839128 PMC4825152

[B43] Sork V. L. Fitz-Gibbon S. T. Puiu D. Crepeau M. Gugger P. F. Sherman R. (2016). First draft assembly and annotation of the genome of a California endemic oak *Quercus lobata* Née (Fagaceae). G3 Genes, Genomes, Genet. 6 (11), 3485–3495. 10.1534/g3.116.030411 27621377 PMC5100847

[B44] Stahl E. A. Dwyer G. Mauricio R. Kreitman M. Bergelson J. (1999). Dynamics of disease resistance polymorphism at the Rpm1 locus of *Arabidopsis* . Nature 400 (6745), 667–671. 10.1038/23260 10458161

[B45] Stam R. Scheikl D. Tellier A. (2016). Pooled enrichment sequencing identifies diversity and evolutionary pressures at NLR resistance genes within a wild tomato population. Genome Biol. Evol. 8 (5), 1501–1515. 10.1093/gbe/evw094 27189991 PMC4898808

[B46] Stamatakis A. (2014). RAxML version 8: a tool for phylogenetic analysis and post-analysis of large phylogenies. Bioinformatics 30 (9), 1312–1313. 10.1093/bioinformatics/btu033 24451623 PMC3998144

[B47] Sun X. Lapin D. Feehan J. M. Stolze S. C. Kramer K. Dongus J. A. (2021). Pathogen effector recognition-dependent association of NRG1 with EDS1 and SAG101 in TNL receptor immunity. Nat. Commun. 12, 3335. 10.1038/s41467-021-23614-x 34099661 PMC8185089

[B64] Tajima F. (1989). Statistical method for testing the neutral mutation hypothesis by DNA polymorphism. Genetics 123, 585–595. 2513255 10.1093/genetics/123.3.585 PMC1203831

[B48] Tellier A. Moreno-Gámez S. Wolfgang S. (2014). Speed of adaptation and genomic signatures in arms race and trench warfare models of host-parasite coevolution. Evolution 68 (8), 11–24. 10.1111/evo.12427 24749791

[B49] Tenhunen J. Geyer R. Banza J. Besson C. Carreiras J. Dinh N. (2009). Assessing ecology, vulnerability and ecosystem services of Mediterranean Oak woodlands. Sci. Pract. Ecol. Restor. (June 2014), 36–54.

[B50] Travis J. M. J. (2003). Climate change and habitat destruction: a deadly anthropogenic cocktail. Proc. R. Soc. B Biol. Sci. 270 (1514), 467–473. 10.1098/rspb.2002.2246 12641900 PMC1691268

[B51] Usié A. Serra O. Barros P. M. Barbosa P. Leão C. Capote T. (2023). An improved reference genome and first organelle genomes of *Quercus suber* . Tree Genet. Genomes 19 (6), 54. 10.1007/s11295-023-01624-8

[B52] Van Ghelder C. Parent G. J. Rigault P. Prunier J. Giguère I. Caron S. (2019). The large repertoire of conifer NLR resistance genes includes drought responsive and highly diversified RNLs. Sci. Rep. 9 (1), 1–13. 10.1038/s41598-019-47950-7 31406137 PMC6691002

[B53] Vannini A. Valentini R. Luisi N. (1996). Impact of drought and *Hypoxylon mediterraneum* on oak decline in the mediterranean region. Ann. For. Sci. 53 (2-3), 753–760. 10.1051/forest:19960251

[B54] Watterson G. A. (1978). The homozygosity test of neutrality. Genetics 88 (2), 405–417. 10.1093/genetics/88.2.405 17248803 PMC1213809

[B55] Wu C. H. Abd-El-Haliem A. Bozkurt T. O. Belhaj K. Terauchi R. Vossen J. H. (2017). NLR network mediates immunity to diverse plant pathogens. Proc. Natl. Acad. Sci. U. S. A. 114 (30), 8113–8118. 10.1073/pnas.1702041114 28698366 PMC5544293

[B56] Yang L. Wang Z. Hua J. (2021). A meta-analysis reveals opposite effects of biotic and abiotic stresses on transcript levels of *arabidopsis* intracellular immune receptor genes. Front. Plant Sci. 12 (March), 1–17. 10.3389/fpls.2021.625729 33747005 PMC7969532

[B57] Yang X. Liu C. Niu X. Wang L. Li L. Yuan Q. (2022). Research on lncRNA related to drought resistance of shanlan upland rice. BMC Genomics 23 (1), 1–15. 10.1186/s12864-022-08546-0 35490237 PMC9055766

[B65] Zdobnov E. M. Apweiler R. (2001). InterProScan - An integration platform for the signature-recognition methods in InterPro. Bioinformatics 17 (9), 847–848. 10.1093/bioinformatics/17.9.847 11590104

[B58] Zhao T. Rui L. Li J. Nishimura M. T. Vogel J. P. Liu N. (2015). A truncated NLR protein, TIR-NBS2, is required for activated defence responses in the exo70B1 mutant. PLoS Genet. 11 (1), 1–28. 10.1371/journal.pgen.1004945 PMC4305288 25617755

