# Supplementary material for: Genome-wide analysis of cork oak NLRs reveals RNL-associated duplications and immune receptor candidates associated with biotic and abiotic stress response
Source: Front Genet. 2026 Oct 7;17:1894037. doi: 10.3389/fgene.2026.1894037 (PMC13683065; doi:10.3389/fgene.2026.1894037)
Supplement: Supplementary file 1 [file DataSheet1.pdf]

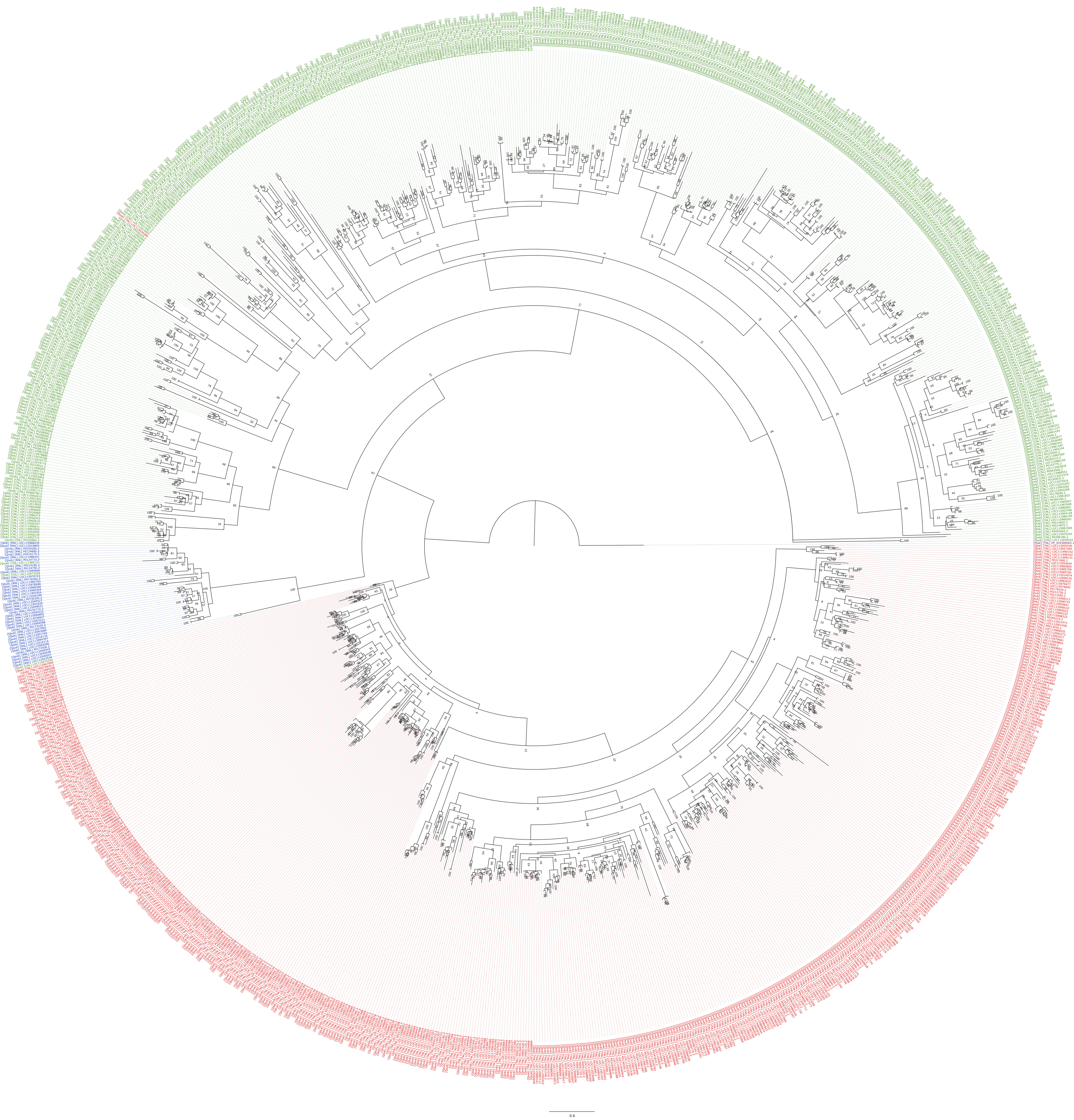
0.6
