# Supplementary material for: Genome-wide analysis of cork oak NLRs reveals RNL-associated duplications and immune receptor candidates associated with biotic and abiotic stress response
Source: Front Genet. 2026 Oct 7;17:1894037. doi: 10.3389/fgene.2026.1894037 (PMC13683065; doi:10.3389/fgene.2026.1894037)
Supplement: Supplementary file 2 [file DataSheet2.pdf]

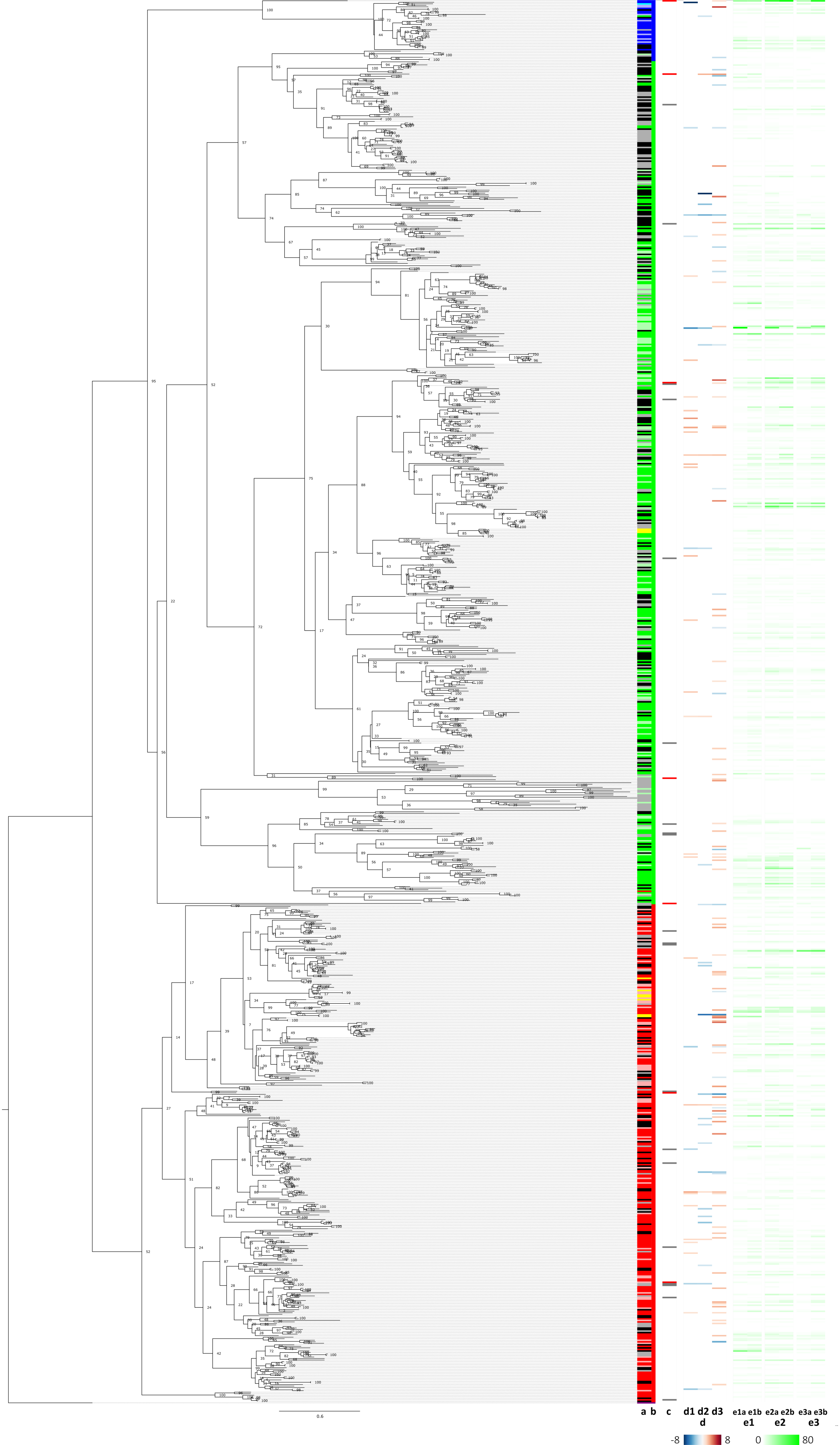

0.6
a b
c
d1 d2 d3
d
e1a e1b
e1
e2a e2b
e2
e3a e3b
e3
-8
8
0
80
