# Supplementary material for: Genome-wide analysis of cork oak NLRs reveals RNL-associated duplications and immune receptor candidates associated with biotic and abiotic stress response
Source: Front Genet. 2026 Oct 7;17:1894037. doi: 10.3389/fgene.2026.1894037 (PMC13683065; doi:10.3389/fgene.2026.1894037)
Supplement: Supplementary file 3 [file DataSheet3.pdf]

Genome-wide analysis of cork oak NLRs reveals RNL-associated duplications and immune receptor candidates associated with biotic and abiotic stress response

Gonçalves, L.M.; Oliveira M.M.; Barros, P.M.

Instituto de Tecnologia Química e Biológica António Xavier, Av. da República, 2780-157 Oeiras, Portugal

Supplementary Tables

Supplementary Table 1: InterNLR and DRAGO3 benchmark summary using the predicted proteome of *Quercus robur*.

Metric	Value
Canonical NLRs detected by InterNLR	358
Canonical NLRs detected by DRAGO3	410
Canonical NLRs detected by both	343
InterNLR-only canonical NLRs	15
DRAGO3-only canonical NLRs	67
Exact classification agreements	339
Discordant classifications	4
Exact agreement rate (%)	339/343 = 98.8%
InterNLR canonical NLRs also detected by DRAGO3 (%)	343/358 = 95.8%
DRAGO3 canonical NLRs also detected by InterNLR (%)	343/410 = 83.7%

Supplementary Table 2: Canonical NLR classifications produced by DRAGO3 and InterNLR for *Quercus robur*.

Gene name	DRAGO3	InterNLR	Detected by both	Exact agreement	Status
Qrob_P0011060.2	CNL	CNL	True	True	Exact agreement
Qrob_P0011310.2	CNL		False	False	DRAGO3 only
Qrob_P0011450.2	CNL	CNL	True	True	Exact agreement
Qrob_P0013430.2	TNL	TNL	True	True	Exact agreement
Qrob_P0015710.2	TNL	TNL	True	True	Exact agreement
Qrob_P0015810.2	TNL		False	False	DRAGO3 only
Qrob_P0016140.2	TNL	TNL	True	True	Exact agreement
Qrob_P0016160.2	TNL	TNL	True	True	Exact agreement
Qrob_P0016280.2	TNL		False	False	DRAGO3 only
Qrob_P0016290.2	TNL	TNL	True	True	Exact agreement
Qrob_P0017620.2	CNL	CNL	True	True	Exact agreement
Qrob_P0021340.2	CNL	CNL	True	True	Exact agreement
Qrob_P0022080.2	CNL	CNL	True	True	Exact agreement
Qrob_P0022300.2	CNL		False	False	DRAGO3 only
Qrob_P0030910.2	TNL	TNL	True	True	Exact agreement
Qrob_P0030970.2	TNL	TNL	True	True	Exact agreement
Qrob_P0031050.2	TNL	TNL	True	True	Exact agreement
Qrob_P0031330.2	TNL	TNL	True	True	Exact agreement
Qrob_P0031350.2	TNL	TNL	True	True	Exact agreement
Qrob_P0032000.2	CNL		False	False	DRAGO3 only
Qrob_P0032060.2	CNL		False	False	DRAGO3 only
Qrob_P0032160.2	CNL		False	False	DRAGO3 only
Qrob_P0036630.2	CNL		False	False	DRAGO3 only
Qrob_P0036640.2	CNL		False	False	DRAGO3 only
Qrob_P0037450.2	TNL	TNL	True	True	Exact agreement
Qrob_P0038510.2	CNL	CNL	True	True	Exact agreement
Qrob_P0038520.2	CNL	CNL	True	True	Exact agreement
Qrob_P0038530.2	CNL	CNL	True	True	Exact agreement
Qrob_P0042350.2	CNL		False	False	DRAGO3 only
Qrob_P0042680.2	CNL	CNL	True	True	Exact agreement
Qrob_P0043190.2	TNL	TNL	True	True	Exact agreement
Qrob_P0043270.2	TNL	TNL	True	True	Exact agreement
Qrob_P0043290.2	TNL	TNL	True	True	Exact agreement
Qrob_P0043300.2	TNL	TNL	True	True	Exact agreement
Qrob_P0043320.2		TNL	False	False	InterNLR only
Qrob_P0046090.2	CNL		False	False	DRAGO3 only
Qrob_P0046630.2	CNL		False	False	DRAGO3 only
Qrob_P0048920.2	TNL	TNL	True	True	Exact agreement
Qrob_P0051120.2	TNL	TNL	True	True	Exact agreement
Qrob_P0051180.2	TNL	TNL	True	True	Exact agreement
Qrob_P0051220.2	TNL	TNL	True	True	Exact agreement
Qrob_P0051240.2	TNL	TNL	True	True	Exact agreement
Qrob_P0051320.2	TNL	TNL	True	True	Exact agreement
Qrob_P0051410.2	TNL	TNL	True	True	Exact agreement
Qrob_P0051420.2	TNL	TNL	True	True	Exact agreement
Qrob_P0051500.2	TNL	TNL	True	True	Exact agreement
Qrob_P0051540.2	TNL	TNL	True	True	Exact agreement
Qrob_P0051580.2	TNL		False	False	DRAGO3 only
Qrob_P0051640.2	TNL	TNL	True	True	Exact agreement
Qrob_P0051670.2	TNL	TNL	True	True	Exact agreement
Qrob_P0051720.2	TNL	TNL	True	True	Exact agreement
Qrob_P0051820.2	TNL	TNL	True	True	Exact agreement
Qrob_P0051840.2	TNL	TNL	True	True	Exact agreement
Qrob_P0054800.2	TNL	TNL	True	True	Exact agreement
Qrob_P0054820.2	TNL	TNL	True	True	Exact agreement
Qrob_P0054840.2	TNL	TNL	True	True	Exact agreement
Qrob_P0062530.2	CNL	CNL	True	True	Exact agreement
Qrob_P0062550.2	CNL	CNL	True	True	Exact agreement
Qrob_P0063390.2	TNL	TNL	True	True	Exact agreement
Qrob_P0063920.2	TNL	TNL	True	True	Exact agreement
Qrob_P0063930.2	TNL	TNL	True	True	Exact agreement
Qrob_P0063940.2	TNL	TNL	True	True	Exact agreement
Qrob_P0068140.2	TNL		False	False	DRAGO3 only
Qrob_P0069290.2	CNL	CNL	True	True	Exact agreement
Qrob_P0069310.2	CNL	CNL	True	True	Exact agreement
Qrob_P0069320.2	CNL		False	False	DRAGO3 only
Qrob_P0069340.2	CNL	CNL	True	True	Exact agreement
Qrob_P0308130.2	CNL	CNL	True	True	Exact agreement
Qrob_P0308140.2	TNL	TNL	True	True	Exact agreement
Qrob_P0308170.2	CNL	CNL	True	True	Exact agreement
Qrob_P0308320.2	CNL	CNL	True	True	Exact agreement
Qrob_P0313210.2	CNL		False	False	DRAGO3 only
Qrob_P0314870.2	CNL	CNL	True	True	Exact agreement
Qrob_P0314920.2	CNL		False	False	DRAGO3 only
Qrob_P0317400.2	TNL	TNL	True	True	Exact agreement
Qrob_P0318580.2	TNL	TNL	True	True	Exact agreement
Qrob_P0318590.2	TNL	TNL	True	True	Exact agreement
Qrob_P0322260.2	CNL	CNL	True	True	Exact agreement
Qrob_P0322330.2	CNL	CNL	True	True	Exact agreement
Qrob_P0327510.2	TNL	TNL	True	True	Exact agreement
Qrob_P0327830.2	TNL	TNL	True	True	Exact agreement
Qrob_P0331560.2	TNL	TNL	True	True	Exact agreement
Qrob_P0333530.2	CNL		False	False	DRAGO3 only
Qrob_P0335200.2	CNL		False	False	DRAGO3 only
Qrob_P0335220.2	TNL	TNL	True	True	Exact agreement
Qrob_P0340040.2	TNL	TNL	True	True	Exact agreement
Qrob_P0341210.2	CNL	CNL	True	True	Exact agreement
Qrob_P0341240.2	CNL	CNL	True	True	Exact agreement
Qrob_P0341250.2	CNL		False	False	DRAGO3 only
Qrob_P0341450.2	CNL	CNL	True	True	Exact agreement
Qrob_P0341470.2	CNL	CNL	True	True	Exact agreement
Qrob_P0341660.2	TNL	TNL	True	True	Exact agreement
Qrob_P0341700.2	TNL	TNL	True	True	Exact agreement
Qrob_P0341710.2	TNL		False	False	DRAGO3 only
Qrob_P0341760.2	TNL	TNL	True	True	Exact agreement
Qrob_P0341810.2	TNL	TNL	True	True	Exact agreement
Qrob_P0341840.2	TNL	TNL	True	True	Exact agreement
Qrob_P0344210.2		TNL	False	False	InterNLR only
Qrob_P0344240.2	TNL	TNL	True	True	Exact agreement
Qrob_P0344330.2	CNL		False	False	DRAGO3 only
Qrob_P0347580.2	TNL	TNL	True	True	Exact agreement
Qrob_P0353030.2	TNL	TNL	True	True	Exact agreement
Qrob_P0360180.2	CNL	CNL	True	True	Exact agreement
Qrob_P0371490.2	CNL	CNL	True	True	Exact agreement
Qrob_P0373130.2	CNL	CNL	True	True	Exact agreement
Qrob_P0373150.2	CNL	CNL	True	True	Exact agreement
Qrob_P0373600.2	TNL	TNL	True	True	Exact agreement
Qrob_P0373660.2	TNL	TNL	True	True	Exact agreement
Qrob_P0373670.2	TNL	TNL	True	True	Exact agreement
Qrob_P0373700.2	TNL	TNL	True	True	Exact agreement
Qrob_P0373710.2	TNL	TNL	True	True	Exact agreement
Qrob_P0380230.2	TNL	TNL	True	True	Exact agreement
Qrob_P0380400.2	TNL		False	False	DRAGO3 only
Qrob_P0380470.2	TNL	TNL	True	True	Exact agreement
Qrob_P0380500.2	TNL	TNL	True	True	Exact agreement
Qrob_P0380530.2	TNL	TNL	True	True	Exact agreement
Qrob_P0380580.2	TNL	TNL	True	True	Exact agreement
Qrob_P0380610.2	TNL	TNL	True	True	Exact agreement
Qrob_P0380650.2	TNL	TNL	True	True	Exact agreement
Qrob_P0380680.2	TNL	TNL	True	True	Exact agreement
Qrob_P0380710.2	TNL	TNL	True	True	Exact agreement
Qrob_P0382760.2	TNL	TNL	True	True	Exact agreement
Qrob_P0391100.2		CNL	False	False	InterNLR only
Qrob_P0391110.2	CNL	CNL	True	True	Exact agreement
Qrob_P0391940.2	CNL		False	False	DRAGO3 only
Qrob_P0392110.2	CNL	CNL	True	True	Exact agreement
Qrob_P0392210.2	CNL		False	False	DRAGO3 only
Qrob_P0396740.2	TNL	TNL	True	True	Exact agreement
Qrob_P0396770.2	TNL	TNL	True	True	Exact agreement
Qrob_P0401620.2	CNL	CNL	True	True	Exact agreement
Qrob_P0401640.2	CNL	CNL	True	True	Exact agreement
Qrob_P0409300.2	CNL		False	False	DRAGO3 only
Qrob_P0409380.2	CNL	CNL	True	True	Exact agreement
Qrob_P0409400.2	CNL	CNL	True	True	Exact agreement

Qrob_P0071030.2	CNL	CNL	True	True	Exact agreement	Qrob_P0409430.2	CNL	CNL	True	True	Exact agreement
Qrob_P0075820.2	TNL	TNL	True	True	Exact agreement	Qrob_P0412700.2	CNL		False	False	DRAGO3 only
Qrob_P0076940.2	CNL	CNL	True	True	Exact agreement	Qrob_P0414540.2	CNL	CNL	True	True	Exact agreement
Qrob_P0076950.2		CNL	False	False	InterNLR only	Qrob_P0415190.2	TNL		False	False	DRAGO3 only
Qrob_P0076970.2	CNL	CNL	True	True	Exact agreement	Qrob_P0416950.2	TNL	TNL	True	True	Exact agreement
Qrob_P0087130.2	CNL		False	False	DRAGO3 only	Qrob_P0417170.2	TNL	TNL	True	True	Exact agreement
Qrob_P0087140.2	CNL		False	False	DRAGO3 only	Qrob_P0417220.2	TNL	TNL	True	True	Exact agreement
Qrob_P0087200.2	CNL		False	False	DRAGO3 only	Qrob_P0422210.2	CNL		False	False	DRAGO3 only
Qrob_P0087210.2	CNL		False	False	DRAGO3 only	Qrob_P0431010.2	TNL	TNL	True	True	Exact agreement
Qrob_P0087220.2	CNL	CNL	True	True	Exact agreement	Qrob_P0432650.2	CNL	CNL	True	True	Exact agreement
Qrob_P0088080.2	TNL	TNL	True	True	Exact agreement	Qrob_P0440440.2	TNL	TNL	True	True	Exact agreement
Qrob_P0088090.2	TNL	TNL	True	True	Exact agreement	Qrob_P0440460.2	TNL	TNL	True	True	Exact agreement
Qrob_P0088890.2	CNL	CNL	True	True	Exact agreement	Qrob_P0449620.2	CNL		False	False	DRAGO3 only
Qrob_P0092630.2	TNL	TNL	True	True	Exact agreement	Qrob_P0452570.2	TNL	TNL	True	True	Exact agreement
Qrob_P0095510.2	CNL	CNL	True	True	Exact agreement	Qrob_P0454540.2	CNL	CNL	True	True	Exact agreement
Qrob_P0103560.2		CNL	False	False	InterNLR only	Qrob_P0454580.2	CNL	CNL	True	True	Exact agreement
Qrob_P0103650.2		CNL	False	False	InterNLR only	Qrob_P0461250.2	TNL	TNL	True	True	Exact agreement
Qrob_P0103770.2	CNL	CNL	True	True	Exact agreement	Qrob_P0468160.2	CNL	RNL	True	False	Discordant
Qrob_P0104010.2	CNL	CNL	True	True	Exact agreement	Qrob_P0470200.2	CNL	CNL	True	True	Exact agreement
Qrob_P0104910.2	TNL	TNL	True	True	Exact agreement	Qrob_P0470210.2	CNL	CNL	True	True	Exact agreement
Qrob_P0108840.2	CNL	CNL	True	True	Exact agreement	Qrob_P0480100.2	CNL	CNL	True	True	Exact agreement
Qrob_P0124680.2		RNL	False	False	InterNLR only	Qrob_P0480400.2	CNL		False	False	DRAGO3 only
Qrob_P0124710.2	CNL	RNL	True	False	Discordant	Qrob_P0480670.2	CNL		False	False	DRAGO3 only
Qrob_P0126890.2	CNL	CNL	True	True	Exact agreement	Qrob_P0480850.2	CNL		False	False	DRAGO3 only
Qrob_P0131710.2	TNL	TNL	True	True	Exact agreement	Qrob_P0482900.2	TNL	TNL	True	True	Exact agreement
Qrob_P0132080.2	TNL		False	False	DRAGO3 only	Qrob_P0486880.2	CNL	CNL	True	True	Exact agreement
Qrob_P0133770.2	TNL	TNL	True	True	Exact agreement	Qrob_P0486950.2	CNL	CNL	True	True	Exact agreement
Qrob_P0133800.2	TNL	TNL	True	True	Exact agreement	Qrob_P0486990.2	CNL	CNL	True	True	Exact agreement
Qrob_P0136540.2	TNL	TNL	True	True	Exact agreement	Qrob_P0489780.2	CNL	CNL	True	True	Exact agreement
Qrob_P0137790.2	CNL		False	False	DRAGO3 only	Qrob_P0489790.2	CNL	CNL	True	True	Exact agreement
Qrob_P0139160.2	CNL		False	False	DRAGO3 only	Qrob_P0492650.2	CNL	CNL	True	True	Exact agreement
Qrob_P0139190.2	CNL		False	False	DRAGO3 only	Qrob_P0497880.2	CNL		False	False	DRAGO3 only
Qrob_P0139210.2	CNL	CNL	True	True	Exact agreement	Qrob_P0498500.2	TNL	TNL	True	True	Exact agreement
Qrob_P0143690.2	CNL	CNL	True	True	Exact agreement	Qrob_P0498620.2	TNL	TNL	True	True	Exact agreement
Qrob_P0145350.2	CNL	CNL	True	True	Exact agreement	Qrob_P0498640.2	TNL	TNL	True	True	Exact agreement
Qrob_P0145420.2	CNL	CNL	True	True	Exact agreement	Qrob_P0498670.2	TNL	TNL	True	True	Exact agreement
Qrob_P0145440.2	CNL	CNL	True	True	Exact agreement	Qrob_P0499470.2	CNL	CNL	True	True	Exact agreement
Qrob_P0146420.2	TNL	TNL	True	True	Exact agreement	Qrob_P0499500.2		CNL	False	False	InterNLR only
Qrob_P0146430.2	TNL	TNL	True	True	Exact agreement	Qrob_P0501480.2	CNL		False	False	DRAGO3 only
Qrob_P0146480.2	TNL	TNL	True	True	Exact agreement	Qrob_P0507750.2	TNL	TNL	True	True	Exact agreement
Qrob_P0155250.2	CNL	CNL	True	True	Exact agreement	Qrob_P0514850.2	CNL	CNL	True	True	Exact agreement
Qrob_P0156450.2	CNL	CNL	True	True	Exact agreement	Qrob_P0514870.2	CNL		False	False	DRAGO3 only
Qrob_P0156470.2	CNL	CNL	True	True	Exact agreement	Qrob_P0519170.2		RNL	False	False	InterNLR only
Qrob_P0156490.2	CNL	CNL	True	True	Exact agreement	Qrob_P0519180.2	CNL	RNL	True	False	Discordant
Qrob_P0156970.2	TNL	TNL	True	True	Exact agreement	Qrob_P0522980.2	CNL	CNL	True	True	Exact agreement
Qrob_P0156990.2	TNL	TNL	True	True	Exact agreement	Qrob_P0523150.2	TNL	TNL	True	True	Exact agreement
Qrob_P0157020.2	TNL	TNL	True	True	Exact agreement	Qrob_P0523180.2	TNL	TNL	True	True	Exact agreement
Qrob_P0157680.2	TNL	TNL	True	True	Exact agreement	Qrob_P0523260.2	TNL	TNL	True	True	Exact agreement
Qrob_P0165870.2	CNL	CNL	True	True	Exact agreement	Qrob_P0523300.2	TNL	TNL	True	True	Exact agreement
Qrob_P0170390.2	TNL	TNL	True	True	Exact agreement	Qrob_P0524130.2	CNL	CNL	True	True	Exact agreement
Qrob_P0170410.2	TNL	TNL	True	True	Exact agreement	Qrob_P0534830.2	TNL	TNL	True	True	Exact agreement
Qrob_P0170420.2	TNL	TNL	True	True	Exact agreement	Qrob_P0534840.2	TNL	TNL	True	True	Exact agreement
Qrob_P0170930.2	CNL		False	False	DRAGO3 only	Qrob_P0534870.2	TNL	TNL	True	True	Exact agreement
Qrob_P0172500.2		RNL	False	False	InterNLR only	Qrob_P0536440.2	TNL	TNL	True	True	Exact agreement
Qrob_P0175660.2		CNL	False	False	InterNLR only	Qrob_P0536460.2	TNL	TNL	True	True	Exact agreement
Qrob_P0178650.2	CNL	CNL	True	True	Exact agreement	Qrob_P0537250.2		CNL	False	False	InterNLR only
Qrob_P0180410.2	CNL	CNL	True	True	Exact agreement	Qrob_P0539070.2	CNL		False	False	DRAGO3 only
Qrob_P0180450.2	CNL		False	False	DRAGO3 only	Qrob_P0539260.2		RNL	False	False	InterNLR only
Qrob_P0180470.2	CNL	CNL	True	True	Exact agreement	Qrob_P0539290.2	CNL		False	False	DRAGO3 only
Qrob_P0180480.2	CNL	CNL	True	True	Exact agreement	Qrob_P0549690.2	CNL	CNL	True	True	Exact agreement
Qrob_P0180490.2	CNL	CNL	True	True	Exact agreement	Qrob_P0549720.2	CNL	CNL	True	True	Exact agreement
Qrob_P0180850.2	CNL	CNL	True	True	Exact agreement	Qrob_P0549770.2	CNL	CNL	True	True	Exact agreement
Qrob_P0180980.2	TNL	TNL	True	True	Exact agreement	Qrob_P0549790.2	CNL	CNL	True	True	Exact agreement
Qrob_P0181170.2	CNL	CNL	True	True	Exact agreement	Qrob_P0549800.2	CNL	CNL	True	True	Exact agreement
Qrob_P0182480.2	CNL	CNL	True	True	Exact agreement	Qrob_P0549820.2	CNL	CNL	True	True	Exact agreement
Qrob_P0182490.2	CNL	CNL	True	True	Exact agreement	Qrob_P0554600.2	TNL	TNL	True	True	Exact agreement
Qrob_P0182530.2	CNL	CNL	True	True	Exact agreement	Qrob_P0554640.2	TNL		False	False	DRAGO3 only
Qrob_P0182590.2	CNL	CNL	True	True	Exact agreement	Qrob_P0556200.2	CNL	CNL	True	True	Exact agreement
Qrob_P0182640.2	CNL	CNL	True	True	Exact agreement	Qrob_P0556320.2	CNL	CNL	True	True	Exact agreement
Qrob_P0182860.2	CNL	CNL	True	True	Exact agreement	Qrob_P0556830.2	TNL	TNL	True	True	Exact agreement
Qrob_P0187820.2	CNL	CNL	True	True	Exact agreement	Qrob_P0563500.2	CNL	CNL	True	True	Exact agreement
Qrob_P0188550.2	TNL	TNL	True	True	Exact agreement	Qrob_P0567330.2	TNL		False	False	DRAGO3 only
Qrob_P0188570.2	TNL	TNL	True	True	Exact agreement	Qrob_P0567390.2	TNL	TNL	True	True	Exact agreement

Qrob_P0190190.2	TNL	TNL	True	True	Exact agreement	Qrob_P0567400.2	TNL	TNL	True	True	Exact agreement
Qrob_P0190410.2	TNL	TNL	True	True	Exact agreement	Qrob_P0568290.2	TNL	TNL	True	True	Exact agreement
Qrob_P0192710.2		RNL	False	False	InterNLR only	Qrob_P0568400.2	TNL	TNL	True	True	Exact agreement
Qrob_P0195530.2	CNL	CNL	True	True	Exact agreement	Qrob_P0568610.2	TNL	TNL	True	True	Exact agreement
Qrob_P0199250.2	CNL	CNL	True	True	Exact agreement	Qrob_P0568910.2	CNL	CNL	True	True	Exact agreement
Qrob_P0201790.2	TNL	TNL	True	True	Exact agreement	Qrob_P0571900.2	CNL	CNL	True	True	Exact agreement
Qrob_P0201900.2	CNL	CNL	True	True	Exact agreement	Qrob_P0571950.2	CNL	CNL	True	True	Exact agreement
Qrob_P0207690.2	CNL	CNL	True	True	Exact agreement	Qrob_P0572010.2	CNL	CNL	True	True	Exact agreement
Qrob_P0207740.2	CNL	CNL	True	True	Exact agreement	Qrob_P0573640.2	CNL	CNL	True	True	Exact agreement
Qrob_P0207790.2	CNL	CNL	True	True	Exact agreement	Qrob_P0574900.2	TNL	TNL	True	True	Exact agreement
Qrob_P0207820.2	CNL	CNL	True	True	Exact agreement	Qrob_P0575810.2	CNL		False	False	DRAGO3 only
Qrob_P0208330.2	CNL	CNL	True	True	Exact agreement	Qrob_P0577430.2	CNL	CNL	True	True	Exact agreement
Qrob_P0208380.2	CNL	CNL	True	True	Exact agreement	Qrob_P0578710.2	CNL	CNL	True	True	Exact agreement
Qrob_P0208400.2	CNL	CNL	True	True	Exact agreement	Qrob_P0578730.2	CNL	CNL	True	True	Exact agreement
Qrob_P0208450.2	CNL	CNL	True	True	Exact agreement	Qrob_P0584810.2	CNL		False	False	DRAGO3 only
Qrob_P0208500.2	CNL	CNL	True	True	Exact agreement	Qrob_P0584830.2	CNL		False	False	DRAGO3 only
Qrob_P0208590.2	CNL	CNL	True	True	Exact agreement	Qrob_P0586420.2	CNL	CNL	True	True	Exact agreement
Qrob_P0211850.2	CNL	CNL	True	True	Exact agreement	Qrob_P0586440.2	CNL		False	False	DRAGO3 only
Qrob_P0212590.2	TNL	TNL	True	True	Exact agreement	Qrob_P0596260.2	CNL	CNL	True	True	Exact agreement
Qrob_P0212660.2	TNL	TNL	True	True	Exact agreement	Qrob_P0596280.2	CNL	CNL	True	True	Exact agreement
Qrob_P0212750.2	TNL	TNL	True	True	Exact agreement	Qrob_P0601410.2	TNL	TNL	True	True	Exact agreement
Qrob_P0213140.2	CNL	CNL	True	True	Exact agreement	Qrob_P0601440.2	TNL	TNL	True	True	Exact agreement
Qrob_P0216990.2	TNL	TNL	True	True	Exact agreement	Qrob_P0603630.2	CNL	CNL	True	True	Exact agreement
Qrob_P0217010.2	TNL	TNL	True	True	Exact agreement	Qrob_P0613830.2		RNL	False	False	InterNLR only
Qrob_P0217090.2	TNL	TNL	True	True	Exact agreement	Qrob_P0614400.2	TNL	TNL	True	True	Exact agreement
Qrob_P0217110.2	TNL		False	False	DRAGO3 only	Qrob_P0619960.2	CNL	CNL	True	True	Exact agreement
Qrob_P0217190.2	TNL	TNL	True	True	Exact agreement	Qrob_P0622080.2	CNL	CNL	True	True	Exact agreement
Qrob_P0218950.2	CNL	CNL	True	True	Exact agreement	Qrob_P0622100.2	CNL	CNL	True	True	Exact agreement
Qrob_P0219910.2	CNL	CNL	True	True	Exact agreement	Qrob_P0627470.2	CNL	CNL	True	True	Exact agreement
Qrob_P0220960.2	CNL	CNL	True	True	Exact agreement	Qrob_P0634660.2	TNL		False	False	DRAGO3 only
Qrob_P0221260.2	TNL	TNL	True	True	Exact agreement	Qrob_P0638690.2	TNL	TNL	True	True	Exact agreement
Qrob_P0221380.2	TNL	TNL	True	True	Exact agreement	Qrob_P0640560.2	TNL	TNL	True	True	Exact agreement
Qrob_P0222430.2	TNL	TNL	True	True	Exact agreement	Qrob_P0643680.2	CNL	CNL	True	True	Exact agreement
Qrob_P0225720.2	CNL	CNL	True	True	Exact agreement	Qrob_P0648660.2	CNL	CNL	True	True	Exact agreement
Qrob_P0234130.2	CNL	CNL	True	True	Exact agreement	Qrob_P0648680.2	CNL	CNL	True	True	Exact agreement
Qrob_P0234190.2	CNL	CNL	True	True	Exact agreement	Qrob_P0654790.2	CNL	CNL	True	True	Exact agreement
Qrob_P0234200.2	CNL	CNL	True	True	Exact agreement	Qrob_P0654810.2	CNL	CNL	True	True	Exact agreement
Qrob_P0234220.2	CNL	CNL	True	True	Exact agreement	Qrob_P0654840.2	CNL	CNL	True	True	Exact agreement
Qrob_P0234240.2	CNL	CNL	True	True	Exact agreement	Qrob_P0655830.2	TNL	TNL	True	True	Exact agreement
Qrob_P0234480.2	CNL	CNL	True	True	Exact agreement	Qrob_P0659890.2	CNL		False	False	DRAGO3 only
Qrob_P0245610.2	CNL	CNL	True	True	Exact agreement	Qrob_P0669060.2	CNL	CNL	True	True	Exact agreement
Qrob_P0246930.2	CNL	CNL	True	True	Exact agreement	Qrob_P0669120.2	CNL	CNL	True	True	Exact agreement
Qrob_P0247090.2	CNL		False	False	DRAGO3 only	Qrob_P0676030.2	TNL	TNL	True	True	Exact agreement
Qrob_P0247940.2	TNL	TNL	True	True	Exact agreement	Qrob_P0676070.2	TNL	TNL	True	True	Exact agreement
Qrob_P0250290.2	TNL	TNL	True	True	Exact agreement	Qrob_P0682370.2	CNL	CNL	True	True	Exact agreement
Qrob_P0250310.2	CNL	CNL	True	True	Exact agreement	Qrob_P0682420.2	CNL	CNL	True	True	Exact agreement
Qrob_P0252290.2	CNL	CNL	True	True	Exact agreement	Qrob_P0683520.2	TNL	TNL	True	True	Exact agreement
Qrob_P0252360.2	CNL	CNL	True	True	Exact agreement	Qrob_P0683560.2	TNL	TNL	True	True	Exact agreement
Qrob_P0254460.2	TNL	TNL	True	True	Exact agreement	Qrob_P0685920.2	CNL	CNL	True	True	Exact agreement
Qrob_P0254480.2	TNL	TNL	True	True	Exact agreement	Qrob_P0688180.2	CNL	CNL	True	True	Exact agreement
Qrob_P0257320.2	TNL	TNL	True	True	Exact agreement	Qrob_P0688560.2	TNL	TNL	True	True	Exact agreement
Qrob_P0260250.2	TNL	TNL	True	True	Exact agreement	Qrob_P0694720.2	CNL		False	False	DRAGO3 only
Qrob_P0272920.2	CNL	CNL	True	True	Exact agreement	Qrob_P0696070.2	TNL		False	False	DRAGO3 only
Qrob_P0275970.2	CNL	CNL	True	True	Exact agreement	Qrob_P0699910.2	TNL	TNL	True	True	Exact agreement
Qrob_P0276250.2	CNL	CNL	True	True	Exact agreement	Qrob_P0702180.2	CNL		False	False	DRAGO3 only
Qrob_P0276760.2	TNL	TNL	True	True	Exact agreement	Qrob_P0709640.2	CNL	CNL	True	True	Exact agreement
Qrob_P0283030.2	CNL	CNL	True	True	Exact agreement	Qrob_P0709650.2	CNL	CNL	True	True	Exact agreement
Qrob_P0285070.2	CNL	CNL	True	True	Exact agreement	Qrob_P0711090.2	TNL	TNL	True	True	Exact agreement
Qrob_P0285160.2	CNL	CNL	True	True	Exact agreement	Qrob_P0711100.2	TNL		False	False	DRAGO3 only
Qrob_P0287480.2	TNL		False	False	DRAGO3 only	Qrob_P0711960.2	TNL	TNL	True	True	Exact agreement
Qrob_P0290060.2	TNL	TNL	True	True	Exact agreement	Qrob_P0711980.2	TNL	TNL	True	True	Exact agreement
Qrob_P0290820.2	CNL	CNL	True	True	Exact agreement	Qrob_P0715920.2	CNL	CNL	True	True	Exact agreement
Qrob_P0290840.2	CNL		False	False	DRAGO3 only	Qrob_P0717120.2	CNL	CNL	True	True	Exact agreement
Qrob_P0290880.2	CNL	CNL	True	True	Exact agreement	Qrob_P0718360.2	CNL	RNL	True	False	Discordant
Qrob_P0291020.2	TNL	TNL	True	True	Exact agreement	Qrob_P0719640.2	CNL	CNL	True	True	Exact agreement
Qrob_P0291040.2	TNL	TNL	True	True	Exact agreement	Qrob_P0719660.2	CNL	CNL	True	True	Exact agreement
Qrob_P0293680.2	CNL	CNL	True	True	Exact agreement	Qrob_P0724940.2	CNL	CNL	True	True	Exact agreement
Qrob_P0294130.2	CNL	CNL	True	True	Exact agreement	Qrob_P0728310.2	CNL		False	False	DRAGO3 only
Qrob_P0294300.2	TNL	TNL	True	True	Exact agreement	Qrob_P0731070.2	CNL		False	False	DRAGO3 only
Qrob_P0294320.2	TNL	TNL	True	True	Exact agreement	Qrob_P0741070.2	TNL	TNL	True	True	Exact agreement
Qrob_P0296180.2	CNL	CNL	True	True	Exact agreement	Qrob_P0746810.2	CNL		False	False	DRAGO3 only
Qrob_P0302010.2	CNL	CNL	True	True	Exact agreement	Qrob_P0759640.2	CNL	CNL	True	True	Exact agreement

Supplementary Table 3: Runtime and resource-usage metrics recorded with /usr/bin/time -v for InterNLR and DRAGO3 using the *Quercus robur* predicted proteome.

Metric	InterNLR	DRAGO3
User time (seconds)	22418.31	0.36
System time (seconds)	437.32	0.29
Percent of CPU this job got	434%	0%
Elapsed (wall clock) time (h:mm:ss)	01:27:36	00:06:34
Average shared text size (kbytes)	0	0
Average unshared data size (kbytes)	0	0
Average stack size (kbytes)	0	0
Average total size (kbytes)	0	0
Maximum resident set size (kbytes)	4981552	24576
Average resident set size (kbytes)	0	0
Major (requiring I/O) page faults	2205	0
Minor (reclaiming a frame) page faults	23442406	11042
Voluntary context switches	4806762	6783
Involuntary context switches	211540	4
Swaps	0	0
File system inputs	0	0
File system outputs	39200312	22392
Socket messages sent	0	0
Socket messages received	0	0
Signals delivered	0	0
Page size (bytes)	4096	4096
Exit status	0	0

Supplementary Table 4: Protein-level evaluation of InterNLR binary NLR detection and architecture agreement using a PRGdb-derived reference set.

PRGDB ID	Gene name	PRGDB classification	InterNLR classification	Binary outcome	Exact architecture agreement
PRGDB135718	FLS2	RLK	No hit	True Negative	NA
PRGDB135719	EFR	RLK	No hit	True Negative	NA
PRGDB135720	PEPR1	RLK	No hit	True Negative	NA
PRGDB135721	ER	RLK	No hit	True Negative	NA
PRGDB1484	HRT	CNL	CNL	True Positive	Yes
PRGDB1489	RCY1	CNL	CNL	True Positive	Yes
PRGDB1491	RPM1	CNL	NL	True Positive	No
PRGDB1492	RPP13	CNL	CNL	True Positive	Yes
PRGDB1493	RPP5	TNL	No hit	False Negative	NA
PRGDB1494	RPP8	CNL	CNL	True Positive	Yes
PRGDB1495	Rps2	CNL	NL	True Positive	No
PRGDB1496	Rps4	TNL	No hit	False Negative	NA
PRGDB1497	RPS5	CNL	CNL	True Positive	Yes
PRGDB150959	RPP1	TNL	TNL	True Positive	Yes
PRGDB150960	RPP4	TNL	TNL	True Positive	Yes
PRGDB150961	RPW8.1	Others	No hit	True Negative	NA
PRGDB150962	RPW8.2	Others	No hit	True Negative	NA
PRGDB150963	RRS1	TNL	TNL	True Positive	Yes
PRGDB150964	RTM1	Others	No hit	True Negative	NA
PRGDB150965	RTM2	Others	No hit	True Negative	NA
PRGDB161441	SSI4	TNL	TNL	True Positive	Yes
PRGDB161447	RPP27	RLP	No hit	True Negative	NA
PRGDB161466	RLM3	TN	N	True Positive	No
PRGDB161467	RAC1	TNL	TNL	True Positive	Yes
PRGDB170016	BAK1	RLK	No hit	True Negative	NA
PRGDB170017	CHS3/DAR4	NL	No hit	False Negative	NA
PRGDB170018	RPP2A	T	TNL	True Positive	No
PRGDB170020	RGA2	N	CNL	True Positive	No
PRGDB170031	PBS1	Others	No hit	True Negative	NA
PRGDB170032	NDR1	Others	No hit	True Negative	NA
PRGDB170038	ADR1	RNL	RNL	True Positive	Yes
PRGDB170041	PSY1R	RLK	No hit	True Negative	NA
PRGDB170048	LECRK55	LECRK	No hit	True Negative	NA
PRGDB170051	LECRK18	LECRK	No hit	True Negative	NA
PRGDB170052	LECRK19	LECRK	No hit	True Negative	NA
PRGDB170053	LECRK42	LECRK	No hit	True Negative	NA
PRGDB170054	LECRK57	LECRK	No hit	True Negative	NA
PRGDB170055	LECRK62	LECRK	No hit	True Negative	NA
PRGDB170056	LECRK13	LECRK	No hit	True Negative	NA
PRGDB170057	LECRK54	LECRK	No hit	True Negative	NA
PRGDB170058	LECRK17	LECRK	No hit	True Negative	NA
PRGDB170059	LECRK82	LECRK	No hit	True Negative	NA
PRGDB170060	LECRK91	LECRK	No hit	True Negative	NA
PRGDB170061	LECRK92	LECRK	No hit	True Negative	NA
PRGDB170062	LORE (SD129)	LECRK	No hit	True Negative	NA
PRGDB170063	LYK5	LYK	No hit	True Negative	NA
PRGDB170064	LYK4	LYK	No hit	True Negative	NA
PRGDB170067	AtCERK1	LYK	No hit	True Negative	NA
PRGDB170070	LYK3	LYK	No hit	True Negative	NA
PRGDB170077	LYM1	LYP	No hit	True Negative	NA
PRGDB170078	LYM3	LYP	No hit	True Negative	NA
PRGDB170081	LYM2	LYP	No hit	True Negative	NA
PRGDB170083	PEPR2	RLK	No hit	True Negative	NA
PRGDB170084	IOS1	RLK	No hit	True Negative	NA
PRGDB170085	PSKR1	RLK	No hit	True Negative	NA
PRGDB170086	SOBIR1	RLK	No hit	True Negative	NA
PRGDB170087	FRK1 (SIRK)	RLK	No hit	True Negative	NA
PRGDB170088	RLP23	RLP	No hit	True Negative	NA
PRGDB170089	RLP42	RLP	No hit	True Negative	NA
PRGDB170090	RLP1	RLP	No hit	True Negative	NA
PRGDB170091	RLP30	RLP	No hit	True Negative	NA

Supplementary Table 5: Binary NLR detection and architecture agreement of InterNLR using PRGdb classifications as reference. Proteins classified by PRGDB as CNL, TNL, RNL, NL, TN, T or N were considered NLRs, whereas RLK, RLP, LECRK, LYK, LYP and "Others" were considered non-NLRs. Any InterNLR classification other than "No hit" was considered a positive NLR detection. Exact architecture agreement was calculated separately by comparing the PRGDB and InterNLR classifications among detected NLRs.

Metric	Evaluation	Calculation	Result
True positive rate/sensitivity	Binary detection	16/19	84.21%
False negative rate	Binary detection	3/19	15.79%
True negative rate/specificity	Binary detection	42/42	100.00%
False positive rate	Binary detection	0/42	0.00%
Precision	Binary detection	16/(16+0)	100.00%
Accuracy	Binary detection	(16+42)/61	95.08%
Exact agreement among detected NLRs	Architecture classification	11/16	68.75%
Discordance among detected NLRs	Architecture classification	5/16	31.25%

Supplementary Table 6: Orthogroups identified through Orthofinder analysis for RNL-clade
*The orthogroup shown in bold corresponds to the orthogroup in which gene duplication was hypothesized in *Quercus suber*.

Orthogroup*	Gene name
**OG0000000**	[RNL]Qlob_XP_030922766.1, [RNL]Qlob_XP_030923397.1, [RNL]Qlob_XP_030973501.1, [RNL]Qlob_XP_030974396.1, [RNL]Qlob_XP_030975078.1, [RNL]Qrob_P0192710.2, [RNL]Qsub_LOC111991820, [RNL]Qsub_LOC112001764, [RNL]Qsub_LOC112016116, [RNL]Qsub_LOC112016135, [RNL]Qsub_LOC112036647, [RNL]Qsub_LOC112040527, [RNL]Qsub_LOC112040545, [RNL]Qsub_LOC112040547, [RNL]Qsub_LOC112040549
OG0000001	[CNL]Qsub_LOC111989151, [RNL]Qlob_XP_030967875.1, [RNL]Qrob_P0124710.2, [RNL]Qrob_P0519180.2, [RNL]Qrob_P0539290.2, [RNL]Qsub_LOC111988241, [RNL]Qsub_LOC112019893
OG0000002	[RNL]Qlob_XP_030974654.1, [RNL]Qlob_XP_030974658.1, [RNL]Qrob_P0172500.2, [RNL]Qrob_P0172530.2, [RNL]Qsub_LOC112003221, [RNL]Qsub_LOC112027002
OG0000003	[CNL]Qlob_XP_030949129.1, [RNL]Qlob_XP_030954044.1, [RNL]Qlob_XP_030956790.1, [RNL]Qrob_P0718360.2, [RNL]Qsub_LOC111985783
OG0000004	[RNL]Qlob_XP_030935137.1, [RNL]Qsub_LOC111991854
OG0000005	[CNL]Qlob_XP_030950362.1, [RNL]Qsub_LOC112022539
OG0000006	[RNL]Qrob_P0124680.2, [RNL]Qrob_P0519170.2
OG0000007	[RNL]Qrob_P0124750.2, [RNL]Qsub_LOC111999566
OG0000008	[RNL]Qrob_P0730350.2, [RNL] Qsub_LOC112026189
OG0000009	[RNL]Qlob_XP_030967699.1
OG0000010	[CNL]Qrob_P0220960.2

Supplementary Table 7: Domain-architecture nomenclature of canonical and non-canonical NLRs used in this study. TIR, Toll/interleukin-1 receptor-like domain; CC, coiled-coil domain; RPW8, Resistance to Powdery Mildew 8-like domain; NB-ARC, nucleotide-binding NB-ARC domain; LRR, leucine-rich repeat domain. These labels describe the detected domain architectures and do not necessarily represent established phylogenetic or functional subclasses.

Class	Detected domain architecture	Definition	Category
N	NB-ARC	NB-ARC domain without a detected N-terminal signalling domain or LRR domain	Non-canonical
NL	NB-ARC–LRR	NB-ARC and LRR domains without a detected N-terminal signalling domain	Non-canonical
CN	CC–NB-ARC	CC and NB-ARC domains without a detected LRR domain	Non-canonical
CNL	CC–NB-ARC–LRR	CC, NB-ARC and LRR domains	Canonical
TN	TIR–NB-ARC	TIR and NB-ARC domains without a detected LRR domain	Non-canonical
TNL	TIR–NB-ARC–LRR	TIR, NB-ARC and LRR domains	Canonical
RN	RPW8–NB-ARC	RPW8-like and NB-ARC domains without a detected LRR domain	Non-canonical
RNL	RPW8–NB-ARC–LRR	RPW8-like, NB-ARC and LRR domains	Canonical
TCNL	TIR–CC–NB-ARC–LRR	TIR, CC, NB-ARC and LRR domains	Non-canonical
TRNL	TIR–RPW8–NB-ARC–LRR	TIR, RPW8-like, NB-ARC and LRR domains	Non-canonical

Supplementary Table 8: Dunn's post-hoc pairwise comparisons of NLR expression levels among domain-architecture classes in cork oak stem tissues. Bonferroni-adjusted P values are reported for comparisons performed separately within each tissue. Adjusted P values below 0.05 were considered statistically significant.

Class 1	Class 2	P-adjusted with Bonferroni	Tissue	Pair	Significant at 0.05
CNL	CN	0.22902	Phellem	CN vs CNL	FALSE
N	CN	0.00012	Phellem	CN vs N	TRUE
NL	CN	1.00000	Phellem	CN vs NL	FALSE
RN	CN	1.00000	Phellem	CN vs RN	FALSE
RNL	CN	1.00000	Phellem	CN vs RNL	FALSE
TCNL	CN	0.23306	Phellem	CN vs TCNL	FALSE
TN	CN	1.00000	Phellem	CN vs TN	FALSE
TNL	CN	1.00000	Phellem	CN vs TNL	FALSE
N	CNL	0.00000	Phellem	CNL vs N	TRUE
NL	CNL	0.00268	Phellem	CNL vs NL	TRUE
RN	CNL	0.41158	Phellem	CNL vs RN	FALSE
RNL	CNL	1.00000	Phellem	CNL vs RNL	FALSE
TCNL	CNL	1.00000	Phellem	CNL vs TCNL	FALSE
TN	CNL	0.07448	Phellem	CNL vs TN	FALSE
TNL	CNL	1.00000	Phellem	CNL vs TNL	FALSE
NL	N	0.00000	Phellem	N vs NL	TRUE
RN	N	1.00000	Phellem	N vs RN	FALSE
RNL	N	0.13883	Phellem	N vs RNL	FALSE
TCNL	N	0.00011	Phellem	N vs TCNL	TRUE
TN	N	0.03490	Phellem	N vs TN	TRUE
TNL	N	0.00000	Phellem	N vs TNL	TRUE
RN	NL	1.00000	Phellem	NL vs RN	FALSE
RNL	NL	1.00000	Phellem	NL vs RNL	FALSE
TCNL	NL	0.11965	Phellem	NL vs TCNL	FALSE
TN	NL	1.00000	Phellem	NL vs TN	FALSE
TNL	NL	1.00000	Phellem	NL vs TNL	FALSE
RNL	RN	1.00000	Phellem	RN vs RNL	FALSE
TCNL	RN	0.06634	Phellem	RN vs TCNL	FALSE
TN	RN	1.00000	Phellem	RN vs TN	FALSE
TNL	RN	1.00000	Phellem	RN vs TNL	FALSE
TCNL	RNL	1.00000	Phellem	RNL vs TCNL	FALSE
TN	RNL	1.00000	Phellem	RNL vs TN	FALSE
TNL	RNL	1.00000	Phellem	RNL vs TNL	FALSE
TN	TCNL	0.10465	Phellem	TCNL vs TN	FALSE
TNL	TCNL	0.69216	Phellem	TCNL vs TNL	FALSE
TNL	TN	1.00000	Phellem	TN vs TNL	FALSE
CNL	CN	0.30657	Inner bark	CN vs CNL	FALSE
N	CN	0.00019	Inner bark	CN vs N	TRUE
NL	CN	1.00000	Inner bark	CN vs NL	FALSE
RN	CN	1.00000	Inner bark	CN vs RN	FALSE
RNL	CN	1.00000	Inner bark	CN vs RNL	FALSE
TCNL	CN	1.00000	Inner bark	CN vs TCNL	FALSE
TN	CN	1.00000	Inner bark	CN vs TN	FALSE
TNL	CN	1.00000	Inner bark	CN vs TNL	FALSE
N	CNL	0.00000	Inner bark	CNL vs N	TRUE
NL	CNL	0.00274	Inner bark	CNL vs NL	TRUE
RN	CNL	1.00000	Inner bark	CNL vs RN	FALSE
RNL	CNL	1.00000	Inner bark	CNL vs RNL	FALSE
TCNL	CNL	1.00000	Inner bark	CNL vs TCNL	FALSE
TN	CNL	0.10528	Inner bark	CNL vs TN	FALSE

TNL	CNL	1.00000	Inner bark	CNL vs TNL	FALSE
NL	N	0.00001	Inner bark	N vs NL	TRUE
RN	N	1.00000	Inner bark	N vs RN	FALSE
RNL	N	0.13454	Inner bark	N vs RNL	FALSE
TCNL	N	0.00614	Inner bark	N vs TCNL	TRUE
TN	N	0.04282	Inner bark	N vs TN	TRUE
TNL	N	0.00000	Inner bark	N vs TNL	TRUE
RN	NL	1.00000	Inner bark	NL vs RN	FALSE
RNL	NL	1.00000	Inner bark	NL vs RNL	FALSE
TCNL	NL	1.00000	Inner bark	NL vs TCNL	FALSE
TN	NL	1.00000	Inner bark	NL vs TN	FALSE
TNL	NL	0.14647	Inner bark	NL vs TNL	FALSE
RNL	RN	1.00000	Inner bark	RN vs RNL	FALSE
TCNL	RN	1.00000	Inner bark	RN vs TCNL	FALSE
TN	RN	1.00000	Inner bark	RN vs TN	FALSE
TNL	RN	1.00000	Inner bark	RN vs TNL	FALSE
TCNL	RNL	1.00000	Inner bark	RNL vs TCNL	FALSE
TN	RNL	1.00000	Inner bark	RNL vs TN	FALSE
TNL	RNL	1.00000	Inner bark	RNL vs TNL	FALSE
TN	TCNL	1.00000	Inner bark	TCNL vs TN	FALSE
TNL	TCNL	1.00000	Inner bark	TCNL vs TNL	FALSE
TNL	TN	0.76570	Inner bark	TN vs TNL	FALSE
CNL	CN	0.11375	Xylem	CN vs CNL	FALSE
N	CN	0.00021	Xylem	CN vs N	TRUE
NL	CN	1.00000	Xylem	CN vs NL	FALSE
RN	CN	1.00000	Xylem	CN vs RN	FALSE
RNL	CN	1.00000	Xylem	CN vs RNL	FALSE
TCNL	CN	1.00000	Xylem	CN vs TCNL	FALSE
TN	CN	1.00000	Xylem	CN vs TN	FALSE
TNL	CN	1.00000	Xylem	CN vs TNL	FALSE
N	CNL	0.00000	Xylem	CNL vs N	TRUE
NL	CNL	0.00197	Xylem	CNL vs NL	TRUE
RN	CNL	1.00000	Xylem	CNL vs RN	FALSE
RNL	CNL	1.00000	Xylem	CNL vs RNL	FALSE
TCNL	CNL	1.00000	Xylem	CNL vs TCNL	FALSE
TN	CNL	0.03541	Xylem	CNL vs TN	TRUE
TNL	CNL	1.00000	Xylem	CNL vs TNL	FALSE
NL	N	0.00000	Xylem	N vs NL	TRUE
RN	N	1.00000	Xylem	N vs RN	FALSE
RNL	N	0.01769	Xylem	N vs RNL	TRUE
TCNL	N	0.05090	Xylem	N vs TCNL	FALSE
TN	N	0.05246	Xylem	N vs TN	FALSE
TNL	N	0.00000	Xylem	N vs TNL	TRUE
RN	NL	1.00000	Xylem	NL vs RN	FALSE
RNL	NL	1.00000	Xylem	NL vs RNL	FALSE
TCNL	NL	1.00000	Xylem	NL vs TCNL	FALSE
TN	NL	1.00000	Xylem	NL vs TN	FALSE
TNL	NL	0.73497	Xylem	NL vs TNL	FALSE
RNL	RN	1.00000	Xylem	RN vs RNL	FALSE
TCNL	RN	1.00000	Xylem	RN vs TCNL	FALSE
TN	RN	1.00000	Xylem	RN vs TN	FALSE
TNL	RN	1.00000	Xylem	RN vs TNL	FALSE
TCNL	RNL	1.00000	Xylem	RNL vs TCNL	FALSE
TN	RNL	1.00000	Xylem	RNL vs TN	FALSE
TNL	RNL	1.00000	Xylem	RNL vs TNL	FALSE
TN	TCNL	1.00000	Xylem	TCNL vs TN	FALSE
TNL	TCNL	1.00000	Xylem	TCNL vs TNL	FALSE
TNL	TN	1.00000	Xylem	TN vs TNL	FALSE

Supplementary Table 9: Number of expressed genes and percentage distribution in the phellem, inner bark and xylem of *Quercus suber* stems. Genes with TPM ≥ 1 were considered expressed.

NLR class	Total number of genes	Number of expressed genes			Percentage of expressed genes		
		Phellem	Inner bark	Xylem	Phellem	Inner bark	Xylem
N	145	34	44	29	**23.448**	**30.345**	**20.000**
NL	231	140	147	126	**60.606**	**63.636**	**54.545**
TN	61	33	37	29	**54.098**	**60.656**	**47.541**
TNL	163	120	127	113	**73.620**	**77.914**	**69.325**
CN	93	48	51	42	**51.613**	**54.839**	**45.161**
CNL	193	154	161	152	**79.793**	**83.42**	**78.756**
RN	7	1	3	2	**14.286**	**42.857**	**28.571**
RNL	18	12	12	11	**66.667**	**66.667**	**61.111**
TCNL	10	9	9	9	**90.000**	**90.000**	**90.000**
TRNL	1	1	1	1	**100.00**	**100.000**	**100.000**

Supplementary Table 10: Differentially expressed canonical and non-canonical NLR-encoding genes in cork oak stem tissues under water-deficit versus well-watered conditions.

Tissue	Gene ID	NLR class	log2FC	P-value	P-adj
Phellem	LOC112014974	TNL	-2.675739206	2.97824E-05	0.001241166
Phellem	LOC112031720	TNL	1.08001288	7.36757E-06	0.000445171
Phellem	LOC112031821	TN	-2.245753378	3.29533E-05	0.001317757
Phellem	LOC112011647	N	1.54570159	0.000120801	0.003384747
Phellem	LOC112034941	TNL	1.321198127	0.003069674	0.032840191
Phellem	LOC111990833	TNL	1.493636079	0.005773674	0.049373815
Phellem	LOC112032324	TNL	1.614586962	0.003512317	0.035927111
Phellem	LOC112038677	TNL	1.552308768	0.002169695	0.026092304
Phellem	LOC112038681	NL	2.854740698	6.85725E-17	1.88293E-13
Phellem	LOC112026127	TNL	-2.198062258	0.001921275	0.023770063
Phellem	LOC111986695	TNL	2.026405657	5.11036E-06	0.000326337
Phellem	LOC111992483	TN	-2.467830461	0.000237842	0.005382592
Phellem	LOC112008762	TNL	1.70815355	0.000398155	0.007747711
Phellem	LOC112039719	CNL	1.670872276	4.56215E-05	0.001689499
Phellem	LOC112036195	NL	1.585502531	0.000341534	0.006901331
Phellem	LOC112031789	NL	1.106479474	0.000799317	0.012802026
Phellem	LOC112006719	NL	1.985482546	5.02009E-06	0.000322238
Phellem	LOC112040649	CNL	1.831786109	0.000604965	0.010447597
Phellem	LOC112002193	CN	2.294129692	0.002647245	0.029605873
Phellem	LOC112026502	CNL	-2.049216295	2.48126E-07	3.03561E-05
Phellem	LOC111999715	CN	1.917347361	0.00445626	0.042001356
Phellem	LOC112018257	NL	2.391362779	3.51973E-13	4.51163E-10
Phellem	LOC112004272	CNL	1.893574138	5.53274E-07	5.44743E-05
Phellem	LOC112035883	NL	2.041857079	0.005696515	0.048966249
Phellem	LOC111997496	CN	2.896847413	0.000986529	0.014884066
Phellem	LOC112035885	NL	3.278682808	3.04282E-07	3.53719E-05
Phellem	LOC112035881	NL	1.802154948	0.001254617	0.017691733
Phellem	LOC111988292	CNL	1.321137147	1.87989E-05	0.000883228
Phellem	LOC112036460	CNL	2.04436271	0.00402453	0.039187632
Phellem	LOC112003710	CN	-4.540781781	4.12911E-08	7.44837E-06
Phellem	LOC111983056	NL	1.286251488	7.3041E-07	6.84402E-05
Phellem	LOC112003579	N	-1.463580693	2.39488E-07	2.95923E-05
Phellem	LOC112012352	NL	-2.469945477	0.005772339	0.049373815
Phellem	LOC112039214	CNL	-1.790753585	1.87931E-06	0.000148381
Phellem	LOC111991854	RNL	-7.427313837	4.5031E-06	0.000295186
Inner bark	LOC112014974	TNL	-1.354199468	0.00410901	0.034237042
Inner bark	LOC112031821	TN	-2.196064093	3.87871E-05	0.000947394
Inner bark	LOC112027139	TNL	-1.501781618	1.8411E-06	7.88546E-05
Inner bark	LOC111990833	TNL	1.443403746	0.006326004	0.047374099
Inner bark	LOC112038928	TNL	-2.918397833	0.002475882	0.023688139
Inner bark	LOC112015684	TNL	-2.125187246	0.003123227	0.028026258
Inner bark	LOC112041155	TNL	-2.353253545	0.004656301	0.037580394
Inner bark	LOC112038681	NL	1.357750484	2.10089E-06	8.81484E-05
Inner bark	LOC112024496	TNL	-3.032490217	0.00147479	0.016027481
Inner bark	LOC112030788	NL	-1.733571474	0.000175645	0.003109387
Inner bark	LOC111986846	NL	-1.710728084	0.003274536	0.028984485
Inner bark	LOC112040795	NL	-2.214053186	0.002948302	0.026935821
Inner bark	LOC111986695	TNL	1.812221891	2.63565E-05	0.000697958
Inner bark	LOC112006457	TNL	-2.88400666	0.002970378	0.027055919
Inner bark	LOC111992337	TNL	-5.523128586	6.64622E-05	0.001469124
Inner bark	LOC112040781	NL	-2.393486692	6.15234E-06	0.000210586
Inner bark	LOC111983244	TN	-1.987790127	0.000766991	0.009611896
Inner bark	LOC112021701	N	-1.568054609	3.30435E-05	0.000831207
Inner bark	LOC112015678	CNL	-1.754542366	0.003911364	0.032956539

Inner bark	LOC112031789	NL	1.263125435	9.79259E-05	0.001970719
Inner bark	LOC112026502	CNL	-1.831246105	2.84081E-07	1.5992E-05
Inner bark	LOC112004272	CNL	1.939595464	1.96095E-07	1.15935E-05
Inner bark	LOC111993234	CNL	-2.051849016	0.000298356	0.004675509
Inner bark	LOC112003710	CN	-3.077035907	0.000178943	0.003151073
Inner bark	LOC112012352	NL	-3.721681523	1.1082E-12	3.03805E-10
Inner bark	LOC112023646	NL	-3.149218611	0.005476399	0.042572583
Inner bark	LOC112004538	NL	-7.855014546	0.004312845	0.035527779
Inner bark	LOC112040104	NL	2.604903763	0.000944265	0.011311501
Inner bark	LOC111989151	CNL	-1.550047368	4.8994E-05	0.001147666
Xylem	LOC111986609	NL	1.603922963	2.32562E-06	2.40071E-05
Xylem	LOC111990144	TN	-3.721703463	1.2522E-07	1.83763E-06
Xylem	LOC112004216	TNL	2.082462456	0.000502917	0.002549453
Xylem	LOC112003242	TNL	1.4704636	0.013610762	0.040201118
Xylem	LOC112006528	TNL	1.342834612	0.016645659	0.047275887
Xylem	LOC112031720	TNL	1.332336903	3.64384E-08	6.10097E-07
Xylem	LOC112030150	TNL	2.434967922	6.55039E-07	7.87353E-06
Xylem	LOC112033266	TNL	1.51871881	4.04671E-05	0.000295964
Xylem	LOC112033265	TNL	2.658488879	1.00179E-10	3.27045E-09
Xylem	LOC112021030	TNL	2.294549812	0.003771129	0.013870503
Xylem	LOC112031990	TNL	2.116308803	1.83827E-05	0.000149734
Xylem	LOC112032222	TNL	2.036695077	0.000386549	0.002040501
Xylem	LOC112008769	NL	1.995264076	0.000347635	0.001866009
Xylem	LOC111997369	TNL	1.452050375	0.002219511	0.008911873
Xylem	LOC112032324	TNL	1.929508553	0.000498523	0.002531854
Xylem	LOC112038677	TNL	1.303706912	0.009260277	0.029290826
Xylem	LOC112038681	NL	2.221753821	1.38996E-13	9.18453E-12
Xylem	LOC112040860	TNL	-1.666782066	0.006101399	0.02077199
Xylem	LOC112024496	TNL	-2.507917845	0.008604985	0.027556693
Xylem	LOC112023290	TNL	3.501331834	0.000682222	0.003311029
Xylem	LOC112005301	TNL	3.050938132	0.000282397	0.001565121
Xylem	LOC112028745	NL	1.931179319	1.57032E-15	1.61026E-13
Xylem	LOC112039222	TN	-1.405480336	0.001304289	0.005682808
Xylem	LOC111987990	TNL	3.515615739	4.04649E-08	6.68902E-07
Xylem	LOC111988622	TNL	-1.06797737	0.012620267	0.037772152
Xylem	LOC111986695	TNL	1.395621377	0.001625921	0.006855723
Xylem	LOC111986398	TNL	2.888519934	0.003031263	0.011564247
Xylem	LOC112006457	TNL	-4.394618306	4.6062E-07	5.80485E-06
Xylem	LOC112004446	TN	3.012742862	0.001920974	0.007922737
Xylem	LOC111986853	NL	-1.175417772	0.001805817	0.007504144
Xylem	LOC111997558	TNL	1.303730264	0.017320316	0.048890572
Xylem	LOC111992483	TN	-1.115738533	0.008063903	0.026212348
Xylem	LOC112002316	NL	2.179131792	0.01320236	0.039186877
Xylem	LOC111987079	TNL	4.207688055	0.000453744	0.002339601
Xylem	LOC111987203	TNL	2.095836102	0.011133687	0.034035972
Xylem	LOC112002846	TCNL	3.657929986	1.25347E-07	1.8384E-06
Xylem	LOC112002841	TCNL	2.971640507	0.000886873	0.004112832
Xylem	LOC111992337	TNL	-4.900814529	0.000419157	0.002184445
Xylem	LOC111989159	TNL	2.685143422	0.000238399	0.001353448
Xylem	LOC111988632	TCNL	-1.213164776	0.013276285	0.039346335
Xylem	LOC112011157	TNL	2.240319424	0.001431939	0.006155418
Xylem	LOC112033116	NL	1.599169115	0.001209026	0.005341199
Xylem	LOC112040780	NL	1.541888587	1.22142E-06	1.36091E-05
Xylem	LOC112009874	N	2.301637631	0.016141386	0.046179911
Xylem	LOC112028520	TNL	1.212188692	0.011551619	0.035087901
Xylem	LOC111986336	TN	2.710377295	0.009541455	0.030018839
Xylem	LOC111988294	NL	2.041025088	0.010968672	0.03363971
Xylem	LOC111992652	TNL	1.738798076	0.006954062	0.023173644

Xylem	LOC112021701	N	-2.162218513	1.37885E-09	3.42812E-08
Xylem	LOC112029450	CNL	1.535370512	2.21051E-07	3.02986E-06
Xylem	LOC112034360	CNL	2.12227611	7.17723E-07	8.56037E-06
Xylem	LOC112039719	CNL	1.47380571	0.000277937	0.001543519
Xylem	LOC112036195	NL	1.087012629	0.007442975	0.024548011
Xylem	LOC112026545	CNL	-2.845909292	0.008536949	0.027384944
Xylem	LOC111996605	CNL	2.279190199	0.016120332	0.046135698
Xylem	LOC112027436	CNL	2.028745155	0.002055778	0.008378041
Xylem	LOC111999847	NL	1.215007873	0.014830714	0.043134217
Xylem	LOC112031836	N	3.17228498	0.007094945	0.023547862
Xylem	LOC111996925	N	1.769797476	0.002354986	0.009343195
Xylem	LOC112029424	CNL	1.794340206	0.001263789	0.00554349
Xylem	LOC112035985	NL	1.606188328	0.008935687	0.028442507
Xylem	LOC112001107	NL	1.688977697	6.09713E-12	2.69813E-10
Xylem	LOC112037238	CNL	-2.044594181	0.001511523	0.006440389
Xylem	LOC112020051	CNL	1.076012581	0.000431026	0.002238275
Xylem	LOC111993842	CNL	1.320025665	0.00647824	0.021838321
Xylem	LOC111990290	NL	-1.135133407	0.000738579	0.003533196
Xylem	LOC112026750	CNL	2.152977832	0.011131305	0.034032902
Xylem	LOC112020850	CNL	2.679711515	0.000469375	0.002407485
Xylem	LOC111988810	CNL	-1.632772185	3.30948E-08	5.59802E-07
Xylem	LOC111992317	CNL	3.564503727	0.000285743	0.001580827
Xylem	LOC112015127	CNL	-1.214848923	0.014743783	0.042896527
Xylem	LOC112004272	CNL	2.114703986	2.41522E-08	4.25427E-07
Xylem	LOC111997496	CN	2.684008615	0.00011976	0.000759074
Xylem	LOC111988580	NL	1.49745703	3.95132E-07	5.07531E-06
Xylem	LOC111988292	CNL	1.019822624	0.006133233	0.020857382
Xylem	LOC111996439	CNL	1.192290625	0.001381614	0.005960862
Xylem	LOC112017201	CN	4.49124366	7.59848E-06	6.89865E-05
Xylem	LOC111993234	CNL	-1.201212528	0.016529538	0.04701283
Xylem	LOC112003915	CN	1.969384544	4.81767E-07	6.04054E-06
Xylem	LOC111983050	CNL	1.341723708	0.011140587	0.034052855
Xylem	LOC112016233	NL	-1.774193517	1.80246E-06	1.91553E-05
Xylem	LOC111990254	N	1.641787649	0.001615786	0.006816354
Xylem	LOC112032511	CNL	-2.17862247	1.93924E-05	0.000156821
Xylem	LOC112002030	NL	1.532599568	0.000421123	0.002192377
Xylem	LOC111998395	NL	2.264684781	0.009402847	0.029673421
Xylem	LOC112012352	NL	-2.944110668	2.69532E-12	1.30607E-10
Xylem	LOC112009952	CNL	3.902147497	0.013046842	0.038809301
Xylem	LOC112006815	N	3.140732296	8.01569E-05	0.000536108
Xylem	LOC112039214	CNL	-1.526599712	5.42044E-05	0.000380447
Xylem	LOC112004707	CNL	-2.007241528	0.017769472	0.049894764
Xylem	LOC112017091	CNL	-2.961742475	0.000710718	0.003421111
Xylem	LOC112040104	NL	3.159824531	9.85856E-06	8.64873E-05
Xylem	LOC112027121	NL	-2.071331778	0.005960593	0.020357121
Xylem	LOC111992140	NL	-1.812220677	0.008862138	0.028248422
Xylem	LOC112019899	RN	4.877028256	1.0278E-09	2.62668E-08
Xylem	LOC112022539	RNL	1.20583523	1.23147E-09	3.10543E-08

Supplementary Table 11: NLRs identified as related to abiotic stress response across several plant species, and with orthologues in *Quercus suber*

* U stands for Uncharacterized
** Orthologues in bold were identified using Orthofinder
*** Only *Arabidopsis* orthologue was referred in the original reference.

Stress	Gene Regulation	Species	Gene name	NLR Class*	Original reference	Blastp against *Arabidopsis* proteome			Orthologue in *Quercus suber***
						Best hit	E-value	Gene Description	
Drought	Up regulation	*Arachis hypogaea*	0MH239.1	CNL	Rizwan et al., 2023	RPM1	4.00E-139	RPM1	**LOC112040104**
Drought	Up regulation	*Arachis hypogaea*	WIN0ZV.1	CNL	Rizwan et al., 2023	RPM1	4.00E-139	RPM1	**LOC112040104**
Drought	Up regulation	*Arachis hypogaea*	UJJ09G5.1	CNL	Rizwan et al., 2023	AT3G14470	1.00E-92	NB-ARC domain-containing disease resistance protein	**LOC112040068**
Drought	Up regulation	*Arachis hypogaea*	3L0H24.1	RNL	Rizwan et al., 2023	ADR1-L1	0	ADR1-like 1	**LOC112022539**
Drought	Up regulation	*Polygonatum kingianum*	c179288.graph_c0	NL	Qian et al., 2021	RPS2***	-	NB-ARC domain-containing disease resistance protein	LOC111992786
Drought	Down regulation	*Oryza sativa*	Os12g0270300	CNL	Yang et al., 2022	RPM1	1.23E-74	NB-ARC domain-containing disease resistance protein	LOC111987192
Drought and osmotic	-	*Nicotiana benthamiana*	VaRGA1	TNL	Li et al., 2017	AT3G14470	0	NB-ARC domain-containing disease resistance protein	**LOC111993947**
Drought	Up regulation	*Arabidopsis thaliana*	ADR1	RNL	Chini et al., 2004	-	-	Disease resistance protein (CC-NBS-LRR class) family	LOC112022539
Osmotic	Up regulation	*Arabidopsis thaliana*	AT5G46490	TNL	Ariga et al., 2017	-	-	Disease resistance protein (TIR-NBS-LRR class) family	**LOC112035762, LOC112035764, LOC112031821**
Osmotic	Up regulation	*Arabidopsis thaliana*	AT5G46500	U	Ariga et al., 2017	-	-	Protein VARIATION IN COMPOUND TRIGGERED ROOT growth protein	**LOC112035762, LOC112035764, LOC112031821**
Osmotic	Up regulation	*Arabidopsis thaliana*	AT5G46510	TNL	Ariga et al., 2017	-	-	Disease resistance protein (TIR-NBS-LRR class) family	**LOC112035762, LOC112035764, LOC112031821**
Osmotic	Up regulation	*Arabidopsis thaliana*	AT5G46520	TNL	Ariga et al., 2017	-	-	Disease resistance protein (TIR-NBS-LRR class) family	**LOC112035762, LOC112035764, LOC112031821**

Drought	Down regulation	*Arabidopsis thaliana*	AT5G63020	CNL	Yang et al., 2021	-	-	Disease resistance protein (CC-NBS-LRR class) family	**LOC111992786**
Drought	Down regulation	*Arabidopsis thaliana*	AT1G50180	CNL	Yang et al., 2021	-	-	NB-ARC domain-containing disease resistance protein	**LOC111990551**
Drought	Down regulation	*Arabidopsis thaliana*	AT1G63360	CNL	Yang et al., 2021	-	-	Disease resistance protein (CC-NBS-LRR class) family	LOC111992786
Drought	Down regulation	*Arabidopsis thaliana*	AT1G53350	CNL	Yang et al., 2021	-	-	Disease resistance protein (CC-NBS-LRR class) family	LOC111990551
Drought	Down regulation	*Arabidopsis thaliana*	AT5G45490	N	Yang et al., 2021	-	-	Probable disease resistance protein	**LOC111996925**
Drought	Down regulation	*Arabidopsis thaliana*	AT5G36930	TNL	Yang et al., 2021	-	-	Disease resistance protein (TIR-NBS-LRR class) family	**LOC112006457**
Drought	Down regulation	*Arabidopsis thaliana*	AT2G17050	TNL	Yang et al., 2021	-	-	Disease resistance protein (TIR-NBS-LRR class)	LOC112034944
Drought	Down regulation	*Arabidopsis thaliana*	AT5G47250	CNL	Yang et al., 2021	-	-	LRR and NB-ARC domains-containing disease resistance protein	LOC111992786
Drought	Down regulation	*Arabidopsis thaliana*	AT5G46450	TNL	Yang et al., 2021	-	-	Disease resistance protein (TIR-NBS-LRR class) family	**LOC112035762, LOC112035764, LOC112031821**
Drought	Down regulation	*Arabidopsis thaliana*	AT1G15890	CNL	Yang et al., 2021	-	-	Disease resistance protein (CC-NBS-LRR class) family	**LOC111992775**
Drought	Down regulation	*Arabidopsis thaliana*	AT1G17600	TNL	Yang et al., 2021	-	-	Disease resistance protein (TIR-NBS-LRR class) family	LOC111995402
Drought	Down regulation	*Arabidopsis thaliana*	AT1G63350	CNL	Yang et al., 2021	-	-	Disease resistance protein (CC-NBS-LRR class) family	LOC111992786
Drought	Down regulation	*Arabidopsis thaliana*	AT5G46260	TNL	Yang et al., 2021	-	-	Disease resistance protein (TIR-NBS-LRR class) family	**LOC112035762, LOC112035764, LOC112031821**
Drought	Down regulation	*Arabidopsis thaliana*	LAZ5	TNL	Yang et al., 2021	-	-	Disease resistance protein (TIR-NBS-LRR class) family	LOC112035366
Drought	Down regulation	*Arabidopsis thaliana*	AT1G66090	TNL	Yang et al., 2021	-	-	Disease resistance protein (TIR-NBS class)	**LOC111987799**

Drought	Up regulation	*Picea glauca*	PG_011798_T.1	RNL	Ghelder et al., 2019	ADR1-L1	1.15E-177	ADR1-like 1	LOC112022539
Drought	Up regulation	*Picea glauca*	PG_018623_T.1	TNL	Ghelder et al., 2019	AT1G69550	3.98E-114	Disease resistance protein (TIR-NBS-LRR class)	**LOC111996063, LOC111996235**
Drought	Up regulation	*Picea glauca*	PG_020032_T.1	RNL	Ghelder et al., 2019	ADR1-L1	9.08E-174	ADR1-like 1	LOC112022539
Drought	Up regulation	*Picea glauca*	PG_021812_T.1	TNL	Ghelder et al., 2019	AT1G27170	7.24E-166	Transmembrane receptors / ATP binding protein	LOC111990393
Drought	Up regulation	*Picea glauca*	PG_000147_T.1	RNL	Ghelder et al., 2019	ADR1-L1	8.03E-159	ADR1-like 1	LOC112022539
Drought	Up regulation	*Picea glauca*	PG_007447_T.1	RNL	Ghelder et al., 2019	ADR1-L1	9.92E-163	ADR1-like 1	LOC112022539
Drought	Up regulation	*Picea glauca*	PG_013399_T.1	RNL	Ghelder et al., 2019	ADR1-L1	1.31E-163	ADR1-like 1	LOC112022539
Drought	Up regulation	*Picea glauca*	PG_002086_T.1	CNL	Ghelder et al., 2019	AT4G27220	1.62E-52	NB-ARC domain-containing disease resistance protein	**LOC112030634**
Drought	Up regulation	*Picea glauca*	PG_005099_T.1	CNL	Ghelder et al., 2019	AT4G27220	6.24E-73	NB-ARC domain-containing disease resistance protein	**LOC112030634**
Drought	Up regulation	*Picea glauca*	PG_005233_T.1	CNL	Ghelder et al., 2019	AT4G27220	3.15E-56	NB-ARC domain-containing disease resistance protein	**LOC112030634**
Drought	Up regulation	*Picea glauca*	PG_013121_T.1	CNL	Ghelder et al., 2019	AT4G27220	3.04E-75	NB-ARC domain-containing disease resistance protein	**LOC112030634**
Drought	Up regulation	*Picea glauca*	PG_033031_T.1	CNL	Ghelder et al., 2019	SUMM2	5.57E-62	LRR and NB-ARC domains-containing disease resistance protein	**LOC112030634**
Drought	Up regulation	*Picea glauca*	PG_027423_T.1	TNL	Ghelder et al., 2019	AT1G27170	4.63E-82	Transmembrane receptors / ATP binding protein	LOC112006452
Drought	Up regulation	*Picea glauca*	PG_003080_T.1	CNL	Ghelder et al., 2019	RPS5	1.05E-31	Disease resistance protein (CC-NBS-LRR class) family	**LOC111996439, LOC112035352, LOC111988289**
Drought	Up regulation	*Picea glauca*	PG_026136_T.1	TNL	Ghelder et al., 2019	AT1G27170	9.04E-71	Transmembrane receptors / ATP binding protein	**LOC111988305**

Drought	Up regulation	*Picea glauca*	PG_033929_T.1	TNL	Ghelder et al., 2019	AT1G27170	1.43E-49	Transmembrane receptors / ATP binding protein	**LOC111996063, LOC111996235**
Drought	Up regulation	*Picea glauca*	PG_000173_T.1	RNL	Ghelder et al., 2019	ADR1-L1	3.34E-148	ADR1-like 1	LOC112022539
Drought	Up regulation	*Picea glauca*	PG_010581_T.1	RNL	Ghelder et al., 2019	ADR1-L1	3.79E-176	ADR1-like 1	LOC112022539
Drought	Up regulation	*Picea glauca*	PG_027346_T.1	TNL	Ghelder et al., 2019	AT1G27170	2.35E-53	Transmembrane receptors / ATP binding protein	**LOC111996063, LOC111996235**
Drought	Up regulation	*Picea glauca*	PG_029408_T.1	CNL	Ghelder et al., 2019	AT4G27220	2.39E-40	NB-ARC domain-containing disease resistance protein	**LOC112030634**
Drought	Up regulation	*Picea glauca*	PG_030995_T.1	CNL	Ghelder et al., 2019	AT5G63020	1.49E-52	Disease resistance protein (CC-NBS-LRR class) family	**LOC112030634**
Drought	Up regulation	*Picea glauca*	PG_010841_T.1	U	Ghelder et al., 2019	CW9	7.35E-12	Disease resistance protein (CC-NBS-LRR class) family	**LOC112021701**
Drought	Up regulation	*Picea glauca*	PG_012802_T.1	RNL	Ghelder et al., 2019	ADR1-L1	6.28E-151	ADR1-like 1	LOC112022539
Drought	Up regulation	*Picea glauca*	PG_019732_T.1	TNL	Ghelder et al., 2019	AT1G27170	7.36E-100	Transmembrane receptors / ATP binding protein	**LOC111988305**

Supplementary Table 12: Per-gene Tajima's D values for expressed NLR loci in Portuguese and Spanish *Quercus suber* populations.

Gene name	NLR class	Tajima's D	Category	Gene name	NLR class	Tajima's D	Category
LOC112007450	NL	0.177856212	Positive	LOC112020971	CNL	1.125222891	Positive
LOC112030540	TNL	0.734943115	Positive	LOC112038176	CNL	0.893620496	Positive
LOC112007497	TNL	1.14453377	Positive	LOC111996025	CNL	0.677213933	Positive
LOC111996906	TNL	1.070587619	Positive	LOC112005491	CNL	-0.839792816	Negative
LOC112007498	TNL	0.929860118	Positive	LOC111991665	CNL	1.826507895	Positive
LOC112004971	TN	0.775855714	Positive	LOC112034352	CN	1.367949467	Positive
LOC112008376	TNL	0.598496982	Positive	LOC112031788	CNL	1.170450714	Positive
LOC112014974	TNL	1.534690717	Positive	LOC111991778	CNL	0.775332171	Positive
LOC112006513	TNL	1.499453659	Positive	LOC111990949	CNL	0.672261142	Positive
LOC112008733	TNL	1.867923333	Positive	LOC112031789	NL	1.042013463	Positive
LOC111986609	NL	0.59613661	Positive	LOC112031794	CNL	1.782089778	Positive
LOC112006510	TNL	1.033866689	Positive	LOC112011752	NL	1.43428187	Positive
LOC112031973	TNL	1.154318182	Positive	LOC112000410	CNL	1.401263167	Positive
LOC112006502	TNL	-0.407529736	Negative	LOC112016666	CNL	1.06015813	Positive
LOC112006512	TNL	1.496608997	Positive	LOC112027065	CNL	0.497886693	Positive
LOC111994714	TNL	1.757236803	Positive	LOC112031787	NL	1.327331778	Positive
LOC112018423	NL	0.738201725	Positive	LOC111996290	CN	0.694149758	Positive
LOC112016323	NL	1.478463705	Positive	LOC112002367	CNL	0.368180448	Positive
LOC112034040	TN	1.171073575	Positive	LOC112037238	CNL	1.192891557	Positive
LOC111991951	TNL	0.760724063	Positive	LOC112006719	NL	0.865055995	Positive
LOC112040885	TNL	0.498610329	Positive	LOC112006714	CNL	1.200621093	Positive
LOC112013990	TNL	0.607871583	Positive	LOC112024831	CN	0.73063705	Positive
LOC112005273	TNL	0.301548454	Positive	LOC111996211	N	1.460555444	Positive
LOC112006518	TN	0.576249282	Positive	LOC112037803	NL	1.476238395	Positive
LOC112006515	N	1.307973794	Positive	LOC112037818	NL	0.449394578	Positive
LOC112023495	N	1.649293818	Positive	LOC112020051	CNL	0.560006925	Positive
LOC112006519	N	1.14637487	Positive	LOC111993158	CNL	1.647129692	Positive
LOC112022566	NL	0.981831628	Positive	LOC111996212	CN	0.815578263	Positive
LOC111989528	TNL	0.351989226	Positive	LOC112000177	CNL	1.596614257	Positive
LOC112022445	NL	1.325559811	Positive	LOC111987503	CN	1.208632724	Positive
LOC112033014	TNL	1.133200121	Positive	LOC112032718	CNL	0.591300494	Positive
LOC112022446	NL	1.25858447	Positive	LOC111997659	NL	0.177125427	Positive
LOC111990141	TN	1.1830601	Positive	LOC111993842	CNL	-0.002619421	Negative
LOC111990142	TNL	-0.111463981	Negative	LOC112022968	NL	1.145440194	Positive
LOC111990144	TN	1.488260742	Positive	LOC112004617	NL	0.931118064	Positive
LOC111990162	TNL	0.497038324	Positive	LOC112009071	NL	1.548287681	Positive
LOC111990131	TNL	1.092247254	Positive	LOC112031022	NL	1.488138585	Positive
LOC112009137	TNL	0.057858383	Positive	LOC112002120	CNL	-0.035066057	Negative
LOC112004216	TNL	-0.179892894	Negative	LOC112023557	CN	0.47738586	Positive
LOC112032715	NL	0.306284393	Positive	LOC112009434	NL	1.626131313	Positive
LOC111986217	N	0.811432695	Positive	LOC111994758	CNL	0.824978253	Positive
LOC111997784	N	1.595522333	Positive	LOC111983981	CNL	0.525615857	Positive
LOC112031742	N	1.268029333	Positive	LOC112033129	NL	0.50062208	Positive
LOC112003242	TNL	0.229128468	Positive	LOC112041073	CN	0.9838285	Positive
LOC112006494	TN	1.465318165	Positive	LOC112003766	CN	0.035334397	Positive
LOC112006528	TNL	1.138583748	Positive	LOC112003645	CNL	1.561786619	Positive
LOC112007176	TNL	1.445678242	Positive	LOC112023828	CNL	0.318372118	Positive
LOC112031720	TNL	0.644811932	Positive	LOC111992492	CNL	0.923396958	Positive
LOC111997489	NL	1.200940469	Positive	LOC111989297	N	1.2899538	Positive
LOC112030150	TNL	0.63557895	Positive	LOC111990290	NL	1.019676548	Positive
LOC112007711	TNL	1.300335047	Positive	LOC111990280	CN	1.748620933	Positive
LOC112033266	TNL	-0.234370179	Negative	LOC112020000	CNL	0.787806387	Positive
LOC112033265	TNL	0.356139297	Positive	LOC112040762	CNL	1.331658	Positive

LOC112033264	TN	1.320884958	Positive	LOC112040649	CNL	1.48127406	Positive
LOC111987799	TN	1.825528184	Positive	LOC112040625	CN	1.005432438	Positive
LOC112025397	TNL	1.312883901	Positive	LOC111986541	CNL	1.36386	Positive
LOC111997330	TN	1.246853817	Positive	LOC112026750	CNL	1.20050248	Positive
LOC112007686	TNL	1.016629971	Positive	LOC112020004	CNL	1.672846344	Positive
LOC112035764	N	0.0911184	Positive	LOC111989316	CNL	1.185788138	Positive
LOC112031821	TN	0.601939492	Positive	LOC111984254	CNL	1.611399	Positive
LOC112035763	TNL	0.725180239	Positive	LOC112020850	CNL	-0.58030399	Negative
LOC112023826	TN	0.439608816	Positive	LOC112020853	NL	0.694831385	Positive
LOC112019791	TNL	0.276886034	Positive	LOC112007546	N	1.666037286	Positive
LOC112019808	TNL	1.738140321	Positive	LOC112025702	NL	0.220709268	Positive
LOC112031990	TNL	0.640621657	Positive	LOC112025680	NL	0.839217335	Positive
LOC112031967	TNL	1.904554135	Positive	LOC112011025	N	1.141715798	Positive
LOC112027139	TNL	1.04256837	Positive	LOC111998514	NL	0.804042418	Positive
LOC112019793	TNL	-0.129161915	Negative	LOC111988264	NL	1.458138541	Positive
LOC112035042	TNL	1.308220863	Positive	LOC111988810	CNL	0.044580214	Positive
LOC112020805	TNL	0.038619788	Positive	LOC111985625	CNL	-0.110378878	Negative
LOC112034914	TNL	0.056581148	Positive	LOC112017844	CN	1.232730647	Positive
LOC112034945	TNL	1.247211181	Positive	LOC112003728	CN	0.89765026	Positive
LOC112011647	N	-0.130891807	Negative	LOC112023395	CNL	1.287441176	Positive
LOC111990523	TNL	1.665971571	Positive	LOC112013952	CNL	1.034336759	Positive
LOC112002069	TN	1.444426628	Positive	LOC112000475	CNL	0.61243769	Positive
LOC112034944	TNL	0.473729286	Positive	LOC111994439	CN	0.647817423	Positive
LOC112040007	TNL	0.050069322	Positive	LOC112001658	CNL	0.748242603	Positive
LOC112030241	TNL	0.476918292	Positive	LOC112003746	CNL	1.385508463	Positive
LOC111984669	TNL	1.007803625	Positive	LOC112001656	CNL	1.431747979	Positive
LOC112034941	TNL	0.310819632	Positive	LOC112013947	CNL	0.786342372	Positive
LOC112030238	TNL	0.434489038	Positive	LOC112040800	CNL	1.5357125	Positive
LOC111990833	TNL	0.286932733	Positive	LOC111994429	CN	0.27141025	Positive
LOC112032222	TNL	0.256887608	Positive	LOC112040069	CN	0.47991797	Positive
LOC112008770	TNL	1.524224205	Positive	LOC112002193	CN	0.719963786	Positive
LOC112024774	NL	0.643078333	Positive	LOC112002191	NL	0.679686887	Positive
LOC112008769	NL	1.28368463	Positive	LOC112034908	N	0.518940972	Positive
LOC112003443	TNL	0.734970296	Positive	LOC112002190	CNL	0.672733577	Positive
LOC112002933	TNL	0.52766936	Positive	LOC112026502	CNL	1.016826237	Positive
LOC111990225	TNL	1.614551353	Positive	LOC111999684	CNL	1.025287682	Positive
LOC111997369	TNL	1.129733248	Positive	LOC112015038	NL	0.369397155	Positive
LOC111990556	NL	0.758362566	Positive	LOC112024582	CNL	1.264914557	Positive
LOC112033727	TNL	0.796371358	Positive	LOC112004120	CNL	0.732453167	Positive
LOC112038928	TNL	0.875984551	Positive	LOC112027814	CNL	0.902588647	Positive
LOC112029593	TNL	0.894006141	Positive	LOC112017945	TCNL	0.811710598	Positive
LOC111991853	TNL	2.04144	Positive	LOC112017956	TCNL	0.662807679	Positive
LOC112015684	TNL	1.428862939	Positive	LOC112017955	TCNL	1.030054946	Positive
LOC112002068	N	1.480479767	Positive	LOC112004138	N	1.000369146	Positive
LOC112032329	NL	1.480854892	Positive	LOC112014093	N	1.2059892	Positive
LOC112041155	TNL	1.586254996	Positive	LOC112004121	CNL	0.574788154	Positive
LOC112013985	TNL	1.400280315	Positive	LOC112038191	CNL	1.04361891	Positive
LOC112032324	TNL	1.618372879	Positive	LOC112014092	NL	1.373836464	Positive
LOC112032332	TNL	1.560455214	Positive	LOC112038182	NL	1.731851444	Positive
LOC111991557	TNL	0.531783505	Positive	LOC112016711	NL	1.400043648	Positive
LOC111991688	TNL	1.20079824	Positive	LOC111999754	CN	0.565913476	Positive
LOC112010617	TNL	1.432694685	Positive	LOC112036463	NL	0.426919696	Positive
LOC111991686	TNL	1.549296	Positive	LOC112019954	CNL	0.873976632	Positive
LOC112038677	TNL	1.185812576	Positive	LOC112036467	CNL	0.721880472	Positive
LOC112038681	NL	1.480804749	Positive	LOC112031603	CN	0.049889	Positive
LOC111987670	NL	1.3716166	Positive	LOC111992317	CNL	0.768668253	Positive
LOC112017266	TNL	1.62977092	Positive	LOC112004157	CNL	0.204311475	Positive
LOC112006731	TNL	1.459924063	Positive	LOC112030558	CNL	1.095774369	Positive

LOC112040863	TNL	0.690281511	Positive	LOC112030560	CNL	0.680933125	Positive
LOC111993423	TN	1.424807889	Positive	LOC112004158	CNL	1.322467286	Positive
LOC112040861	TNL	1.369023909	Positive	LOC112015127	CNL	0.782459547	Positive
LOC111994594	TNL	1.212340074	Positive	LOC111993297	CNL	1.119448263	Positive
LOC111993417	TN	0.25396474	Positive	LOC112036114	CNL	1.236820926	Positive
LOC112028667	TNL	1.208337102	Positive	LOC112012304	CNL	-0.217374837	Negative
LOC111992801	TNL	1.656060136	Positive	LOC112019753	CN	0.691627938	Positive
LOC112040860	TNL	0.42979916	Positive	LOC112015473	CNL	0.681820765	Positive
LOC112024496	TNL	1.040832632	Positive	LOC112035784	CNL	0.592019621	Positive
LOC111993428	NL	1.35006866	Positive	LOC112015472	CNL	0.886396117	Positive
LOC111993422	TNL	1.006238308	Positive	LOC112023805	CN	1.467077067	Positive
LOC112035366	NL	1.434583167	Positive	LOC112038431	CNL	1.223621451	Positive
LOC112016813	TNL	1.596451119	Positive	LOC111997194	CNL	0.811641744	Positive
LOC112000484	NL	0.37336043	Positive	LOC112015126	CNL	1.118650055	Positive
LOC112030847	TNL	1.17209617	Positive	LOC111999911	CNL	0.178824648	Positive
LOC111991547	TNL	1.387345953	Positive	LOC111999919	CNL	0.403388907	Positive
LOC112025098	TNL	1.029626488	Positive	LOC111999715	CN	0.898603178	Positive
LOC112035359	TNL	1.611702516	Positive	LOC112034131	CNL	0.347762733	Positive
LOC111995402	TNL	1.857996	Positive	LOC112018257	NL	-0.063824056	Negative
LOC112026127	TNL	-0.129157353	Negative	LOC112038916	CNL	1.208354471	Positive
LOC112034532	N	0.938044294	Positive	LOC112017966	NL	0.601934601	Positive
LOC112024295	TNL	1.397023667	Positive	LOC112025912	NL	1.184096761	Positive
LOC112030783	TNL	1.518941226	Positive	LOC112025911	N	1.572545519	Positive
LOC112030788	NL	1.67081619	Positive	LOC112004272	CNL	0.6807879	Positive
LOC112004966	TNL	1.458812468	Positive	LOC112038921	NL	1.232615702	Positive
LOC112030785	TNL	1.568880287	Positive	LOC111994765	N	1.224702818	Positive
LOC112030777	TN	1.446576619	Positive	LOC112026293	CNL	1.127577873	Positive
LOC112004967	NL	1.007937962	Positive	LOC112028650	CN	0.760311233	Positive
LOC112012553	NL	0.98466498	Positive	LOC111990648	CNL	0.840909389	Positive
LOC111986846	NL	1.187593385	Positive	LOC112028655	CN	1.0663815	Positive
LOC112030782	NL	0.530228968	Positive	LOC112035883	NL	0.832934971	Positive
LOC112005301	TNL	1.303007469	Positive	LOC111988568	N	0.395191512	Positive
LOC112040795	NL	1.433507667	Positive	LOC112006792	CN	-0.64293729	Negative
LOC112028745	NL	0.184063535	Positive	LOC111988580	NL	0.846901616	Positive
LOC112029340	TNL	1.04785	Positive	LOC112006516	CNL	-0.335776	Negative
LOC112039222	TN	-0.030624241	Negative	LOC111997494	NL	1.028787907	Positive
LOC112039221	TNL	0.304037018	Positive	LOC112009533	NL	1.609068053	Positive
LOC111987990	TNL	1.202782563	Positive	LOC112035885	NL	0.350919123	Positive
LOC111988624	NL	-0.230261211	Negative	LOC112037830	N	0.772185238	Positive
LOC111988622	TNL	0.211019455	Positive	LOC112006262	N	0.957635711	Positive
LOC112004424	TNL	0.483567077	Positive	LOC111989889	CNL	0.945756543	Positive
LOC111986695	TNL	1.295800431	Positive	LOC112038999	CNL	1.291579251	Positive
LOC111994595	TN	1.34973825	Positive	LOC112035881	NL	0.285421094	Positive
LOC112035375	TN	2.073553875	Positive	LOC112028082	NL	0.949651909	Positive
LOC111986398	TNL	1.2676212	Positive	LOC112017197	CN	1.586663789	Positive
LOC112006457	TNL	0.426011606	Positive	LOC111986156	NL	1.839980333	Positive
LOC112006452	TNL	0.980243592	Positive	LOC111988289	NL	1.222681167	Positive
LOC111986000	TNL	1.345200276	Positive	LOC112017196	N	1.994627467	Positive
LOC111993966	NL	0.74623	Positive	LOC111988292	CNL	-0.132123167	Negative
LOC112000378	N	1.544075881	Positive	LOC111988288	NL	1.697382167	Positive
LOC112038686	NL	1.485153333	Positive	LOC111986158	NL	1.083498581	Positive
LOC111986853	NL	1.6317788	Positive	LOC112012781	NL	1.265899257	Positive
LOC112002317	N	0.442694909	Positive	LOC111996427	CNL	1.159933141	Positive
LOC111986997	NL	1.144012738	Positive	LOC111986155	CNL	1.680934724	Positive
LOC112009430	TN	1.587836265	Positive	LOC112035352	CNL	1.252703341	Positive
LOC112004710	TN	0.910434817	Positive	LOC111996439	CNL	0.547790834	Positive
LOC111986990	TN	1.060361877	Positive	LOC112010357	CN	0.501863542	Positive
LOC112039062	TNL	1.10543228	Positive	LOC111988332	CNL	0.454984695	Positive

LOC112020092	TNL	1.3329974	Positive	LOC111997534	CNL	-0.170732667	Negative
LOC112039068	TN	0.540732963	Positive	LOC112000199	CNL	0.30245	Positive
LOC112031142	NL	0.987627351	Positive	LOC112036460	CNL	0.520595934	Positive
LOC112039065	N	0.773580292	Positive	LOC112041050	N	1.278468769	Positive
LOC111997558	TNL	0.760669266	Positive	LOC112002873	CNL	1.618175087	Positive
LOC112020086	TNL	0.2392975	Positive	LOC112000221	CNL	1.143219063	Positive
LOC111992483	TN	0.980607261	Positive	LOC112011137	CNL	1.790734725	Positive
LOC112002316	NL	0.622996056	Positive	LOC112002877	CNL	0.913516461	Positive
LOC112024913	NL	1.588563269	Positive	LOC112031112	CN	-0.45581665	Negative
LOC112024915	TNL	1.063999508	Positive	LOC111993234	CNL	0.92878925	Positive
LOC111992628	TNL	0.623503017	Positive	LOC112008190	CN	-0.154950167	Negative
LOC112026695	TNL	1.140549858	Positive	LOC112002878	CN	1.180015947	Positive
LOC111989814	TNL	1.294114619	Positive	LOC112023379	CNL	1.308029643	Positive
LOC112026690	NL	0.902539277	Positive	LOC112033293	CNL	0.12745734	Positive
LOC112032185	TNL	1.257377098	Positive	LOC112003711	CN	1.256887	Positive
LOC112012327	TN	1.596082548	Positive	LOC112003710	CN	0.939191593	Positive
LOC112012952	TNL	0.833592383	Positive	LOC112003915	CN	0.820002773	Positive
LOC111992630	NL	1.359397398	Positive	LOC112032108	CN	1.424996095	Positive
LOC112032853	TN	-0.047108147	Negative	LOC112015148	CN	1.524550545	Positive
LOC111989793	TN	0.828306109	Positive	LOC112015032	CNL	1.300080709	Positive
LOC112027080	NL	0.319134566	Positive	LOC112025887	CN	0.952096539	Positive
LOC112002846	TCNL	0.481559834	Positive	LOC112015149	CNL	1.036288209	Positive
LOC112002841	TCNL	1.181822025	Positive	LOC112003706	CNL	1.638266486	Positive
LOC111992337	TNL	1.68620289	Positive	LOC112025763	CN	1.357849714	Positive
LOC112023043	TNL	0.148852971	Positive	LOC112003713	CN	1.543199167	Positive
LOC112015885	TNL	-0.170159845	Negative	LOC112003941	N	1.524761125	Positive
LOC111989159	TNL	1.395438288	Positive	LOC111986159	CNL	1.16923051	Positive
LOC112035550	TN	1.183930866	Positive	LOC112021740	CN	0.658446062	Positive
LOC112024520	TNL	1.600033333	Positive	LOC112004128	CNL	0.819331319	Positive
LOC112004425	TN	1.14991214	Positive	LOC112004427	CNL	1.220856584	Positive
LOC111988623	TCNL	0.257989	Positive	LOC112004418	N	0.506119932	Positive
LOC112020992	TCNL	1.175209998	Positive	LOC112033368	CN	1.409144429	Positive
LOC111988632	TCNL	0.40135591	Positive	LOC111988536	CN	1.716633667	Positive
LOC112011165	TN	0.040566275	Positive	LOC112012839	CN	1.270466685	Positive
LOC112011157	TNL	1.476234608	Positive	LOC111983045	CN	1.611407882	Positive
LOC111988628	TN	-0.158855709	Negative	LOC112018510	CNL	1.618271937	Positive
LOC112009942	TN	-0.1859011	Negative	LOC111983050	CNL	1.273050111	Positive
LOC112004338	NL	0.233386339	Positive	LOC111983056	NL	0.240859538	Positive
LOC112004336	NL	0.127784183	Positive	LOC111988543	CNL	1.077546123	Positive
LOC111998804	TNL	0.735833461	Positive	LOC112016233	NL	0.151975545	Positive
LOC111992059	TCNL	-0.187685959	Negative	LOC112019991	CNL	-0.592844667	Negative
LOC112040808	TNL	0.625516317	Positive	LOC111990254	N	1.604191	Positive
LOC112033116	NL	0.407718542	Positive	LOC112032521	NL	0.642533044	Positive
LOC112040780	NL	0.620108026	Positive	LOC112017526	CNL	0.262261133	Positive
LOC112009874	N	1.406059	Positive	LOC111990263	CN	0.451686245	Positive
LOC112032478	TN	1.554254857	Positive	LOC112013887	CN	-0.317570727	Negative
LOC112040805	TNL	0.765649741	Positive	LOC112013901	CN	-0.528224231	Negative
LOC112001729	NL	1.550207047	Positive	LOC112032512	CNL	1.183947455	Positive
LOC112040781	NL	1.439767223	Positive	LOC112030753	NL	1.148996316	Positive
LOC112009868	TNL	1.041890547	Positive	LOC112032511	CNL	-0.026203125	Negative
LOC111983244	TN	1.190925222	Positive	LOC112030752	CNL	1.962619304	Positive
LOC112009867	NL	1.571830176	Positive	LOC112032514	CNL	1.056746263	Positive
LOC112008762	TNL	1.252288151	Positive	LOC112003579	N	0.121439987	Positive
LOC112003588	TN	2.130815	Positive	LOC112002030	NL	-0.849392	Negative
LOC112028520	TNL	1.028760171	Positive	LOC112010906	CNL	2.2241675	Positive
LOC112019395	TNL	0.878482915	Positive	LOC112001962	NL	-0.772059669	Negative
LOC112012965	TNL	0.241866988	Positive	LOC111996953	CNL	0.956203047	Positive
LOC112029886	TNL	0.052601524	Positive	LOC112026542	NL	0.721648656	Positive

LOC111996235	N	1.4051592	Positive	LOC111990551	NL	1.20537122	Positive
LOC112017723	NL	0.974503386	Positive	LOC111998395	NL	0.888296436	Positive
LOC112032177	TNL	0.795826162	Positive	LOC112017527	NL	0.519895974	Positive
LOC111988301	TNL	0.990925589	Positive	LOC112013888	NL	-0.319167805	Negative
LOC111988305	TN	0.391465244	Positive	LOC112011798	N	-0.108427029	Negative
LOC111986336	TN	0.664860748	Positive	LOC112012352	NL	1.308165725	Positive
LOC111988300	TNL	1.619053238	Positive	LOC112030882	NL	0.369420495	Positive
LOC111988294	NL	1.327974581	Positive	LOC111985659	CNL	2.005416667	Positive
LOC111992652	TNL	1.295639252	Positive	LOC112015797	CNL	1.404544333	Positive
LOC112022726	TN	0.361038557	Positive	LOC112023646	NL	1.10587025	Positive
LOC112022723	NL	1.556682161	Positive	LOC112002294	CNL	0.424740047	Positive
LOC112022741	TNL	0.408063178	Positive	LOC112003530	CNL	0.723767869	Positive
LOC112036504	NL	1.346891239	Positive	LOC112028360	CNL	0.503848619	Positive
LOC112036521	TN	1.231197441	Positive	LOC112032592	NL	1.742981643	Positive
LOC111985982	NL	0.3663389	Positive	LOC112034761	NL	1.532140994	Positive
LOC112021701	N	-0.183065507	Negative	LOC112029972	NL	0.748052118	Positive
LOC111992484	CNL	1.244453067	Positive	LOC112036375	N	-0.098323	Negative
LOC111986557	NL	1.582362103	Positive	LOC112006815	N	0.558021167	Positive
LOC112006919	CNL	0.195760713	Positive	LOC112036388	NL	0.667523971	Positive
LOC111992494	CNL	0.199813798	Positive	LOC112029975	N	0.811235	Positive
LOC112034381	CNL	0.878795222	Positive	LOC112036382	NL	1.056733495	Positive
LOC112034384	TNL	1.133953646	Positive	LOC112029973	NL	1.112181596	Positive
LOC112030620	CNL	1.013861335	Positive	LOC112006810	N	1.504840233	Positive
LOC112027383	NL	1.191557406	Positive	LOC112037989	N	1.29176569	Positive
LOC112027363	CNL	0.256264998	Positive	LOC112006807	N	-0.284572744	Negative
LOC112032248	NL	0.194862857	Positive	LOC112029832	N	1.027613387	Positive
LOC112006682	CNL	-0.089873808	Negative	LOC112036377	NL	1.147990037	Positive
LOC112032250	CNL	1.784615636	Positive	LOC112039214	CNL	0.755745198	Positive
LOC111984752	CNL	1.220641098	Positive	LOC112036408	CNL	1.169872195	Positive
LOC111986578	CNL	1.447773064	Positive	LOC112029957	N	1.136780909	Positive
LOC112038005	CNL	0.518450422	Positive	LOC112036381	N	0.842243113	Positive
LOC111997425	CNL	1.445712647	Positive	LOC112038437	NL	1.371196273	Positive
LOC112028507	CNL	1.227509585	Positive	LOC111984021	NL	1.081376656	Positive
LOC112037979	CNL	1.009885723	Positive	LOC112016456	N	-0.149316333	Negative
LOC111987136	CNL	0.446427088	Positive	LOC111991885	NL	1.055327222	Positive
LOC112037987	NL	1.366363593	Positive	LOC111991882	N	0.460801627	Positive
LOC112029450	CNL	0.770783005	Positive	LOC111984607	NL	0.789118825	Positive
LOC111998942	CNL	-0.033654204	Negative	LOC111987185	NL	0.852247763	Positive
LOC112015676	CN	1.102029425	Positive	LOC112036529	N	1.081608692	Positive
LOC112015678	CNL	1.362484968	Positive	LOC111987189	NL	1.012755686	Positive
LOC112041194	CNL	0.758353359	Positive	LOC111999746	NL	1.646378563	Positive
LOC112034360	CNL	-0.186344271	Negative	LOC112007111	NL	1.318626806	Positive
LOC111996945	CNL	1.75129922	Positive	LOC111992130	NL	0.557643581	Positive
LOC112013955	CNL	0.824718189	Positive	LOC112029496	NL	0.922312492	Positive
LOC112026545	CNL	0.574541345	Positive	LOC112004707	CNL	1.036475042	Positive
LOC112034450	NL	1.267251412	Positive	LOC111996003	NL	1.506283109	Positive
LOC111996605	CNL	1.253259272	Positive	LOC112001973	N	1.389794243	Positive
LOC112031856	NL	0.5696858	Positive	LOC111987187	NL	1.483211308	Positive
LOC111996373	CN	0.892464752	Positive	LOC112017093	CN	1.484081653	Positive
LOC112026960	CNL	0.254855914	Positive	LOC112017091	CNL	1.725085139	Positive
LOC111996375	CNL	1.256239063	Positive	LOC112040104	NL	0.102553594	Positive
LOC112012356	N	0.462557364	Positive	LOC111992374	NL	1.091369357	Positive
LOC111992775	N	1.189689273	Positive	LOC111992372	NL	1.253002045	Positive
LOC111992786	CNL	1.945316261	Positive	LOC112027121	NL	1.016476454	Positive
LOC112032085	CNL	0.6866772	Positive	LOC111984239	NL	0.981221761	Positive
LOC112030634	CNL	0.4978265	Positive	LOC112022408	N	-0.009194	Negative
LOC111999847	NL	-0.227487323	Negative	LOC111992368	NL	0.955560746	Positive
LOC112002329	CNL	1.463636343	Positive	LOC112028692	N	-0.175511	Negative

LOC111985987	CNL	0.29679743	Positive	LOC111992370	NL	1.81267272	Positive
LOC112024017	CNL	1.70556466	Positive	LOC111992140	NL	0.857531173	Positive
LOC111995435	NL	0.052565307	Positive	LOC111985159	NL	1.006000836	Positive
LOC111996925	N	1.36366075	Positive	LOC112026189	RNL	-0.559113667	Negative
LOC112029424	CNL	-0.15598	Negative	LOC112036679	NL	0.4566778	Positive
LOC111984311	CNL	1.190000356	Positive	LOC111991848	NL	1.3307715	Positive
LOC112002129	NL	0.708029095	Positive	LOC111991843	NL	-0.144603929	Negative
LOC112022969	CNL	0.980422066	Positive	LOC112040527	RNL	1.10213201	Positive
LOC112022972	NL	1.175164933	Positive	LOC112016116	RNL	-0.37520955	Negative
LOC112006865	N	0.634941358	Positive	LOC112001765	RN	0.515822547	Positive
LOC112035985	NL	1.145674471	Positive	LOC112016135	RNL	-0.202248682	Negative
LOC112007233	CN	0.338003429	Positive	LOC112001764	RNL	0.4218568	Positive
LOC112019090	CNL	1.723296929	Positive	LOC112027002	RNL	1.19817325	Positive
LOC112022674	CN	0.410985631	Positive	LOC112003221	RNL	2.247115	Positive
LOC112011339	NL	1.591607781	Positive	LOC112040545	RNL	-0.114818382	Negative
LOC112001109	NL	1.02328497	Positive	LOC111989151	CNL	-0.119442126	Negative
LOC112001107	NL	0.408758077	Positive	LOC112019893	RNL	0.768283768	Positive
LOC112001111	CNL	1.6581451	Positive	LOC111988241	RNL	1.504597785	Positive
LOC112001106	CN	1.515853786	Positive	LOC112006709	NL	-0.076277455	Negative
LOC111993947	CNL	1.160565985	Positive	LOC111985783	RNL	0.441357985	Positive
LOC112010457	CNL	1.38415148	Positive	LOC112013988	RN	1.154717783	Positive
LOC112020968	CNL	1.161274592	Positive	LOC112013981	TRNL	0.400414631	Positive
LOC112020969	CNL	1.684037133	Positive	LOC111991854	RNL	1.56451405	Positive
